# Molecular evolution and functional characterisation of an ancient phenylalanine ammonia-lyase gene (NnPAL1) from Nelumbo nucifera: novel insight into the evolution of the PAL family in angiosperms

**DOI:** 10.1186/1471-2148-14-100

**Published:** 2014-05-09

**Authors:** Zhihua Wu, Songtao Gui, Shuzhen Wang, Yi Ding

**Affiliations:** 1 State Key Laboratory of Hybrid Rice, Department of Genetics, College of Life Sciences, Wuhan University, Wuhan, Hubei Province 430072, People’s Republic of China; 2 College of Life Sciences, Huanggang Normal University, Huanggang, Hubei Province 438000, People’s Republic of China

**Keywords:** *Nelumbo nucifera*, Phenylalanine ammonia-lyase gene, Evolution, Expression, Bioinformatics analysis

## Abstract

**Background:**

Phenylalanine ammonia-lyase (PAL; E.C.4.3.1.5) is a key enzyme of the phenylpropanoid pathway in plant development, and it catalyses the deamination of phenylalanine to trans-cinnamic acid, leading to the production of secondary metabolites. This enzyme has been identified in many organisms, ranging from prokaryotes to higher plants. Because *Nelumbo nucifera* is a basal dicot rich in many secondary metabolites, it is a suitable candidate for research on the phenylpropanoid pathway.

**Results:**

Three PAL members, *NnPAL1*, *NnPAL2* and *NnPAL3*, have been identified in *N. nucifera* using genome-wide analysis*. NnPAL1* contains two introns; however, both *NnPAL2* and *NnPAL3* have only one intron. Molecular and evolutionary analysis of *NnPAL1* confirms that it is an ancient PAL member of the angiosperms and may have a different origin. However, PAL clusters, except *NnPAL1*, are monophyletic after the split between dicots and monocots. These observations suggest that duplication events remain an important occurrence in the evolution of the PAL gene family. Molecular assays demonstrate that the mRNA of the *NnPAL1* gene is 2343 bp in size and encodes a 717 amino acid polypeptide. The optimal pH and temperature of the recombinant NnPAL1 protein are 9.0 and 55°C, respectively. The NnPAL1 protein retains both PAL and weak TAL catalytic activities with K_m_ values of 1.07 mM for L-phenylalanine and 3.43 mM for L-tyrosine, respectively. Cis-elements response to environmental stress are identified and confirmed using real-time PCR for treatments with abscisic acid (ABA), indoleacetic acid (IAA), ultraviolet light, *Neurospora crassa* (fungi) and drought.

**Conclusions:**

We conclude that the angiosperm PAL genes are not derived from a single gene in an ancestral angiosperm genome; therefore, there may be another ancestral duplication and vertical inheritance from the gymnosperms. The different evolutionary histories for PAL genes in angiosperms suggest different mechanisms of functional regulation. The expression patterns of *NnPAL1* in response to stress may be necessary for the survival of *N. nucifera* since the Cretaceous Period. The discovery and characterisation of the ancient *NnPAL1* help to elucidate PAL evolution in angiosperms.

## Background

The phenylpropanoid pathway is an important branch of the plant secondary metabolism pathways that produces many essential secondary metabolites. In this pathway, secondary metabolic products, such as lignin, flavonoids and coumarins, play important roles in plant growth, development, mechanical support, and disease resistance [1,2]. Phenylalanine ammonia-lyase (PAL; E.C.4.3.1.5) is the first and key enzyme between primary and secondary metabolism, and it catalyses the biotransformation of L-phenylalanine to trans-cinnamic acid. The synthesis of many secondary metabolites, such as flavonoids, flavonols, anthocyanins, condensed tannins, lignins, coumarins, and ubiquinone occur downstream of the phenylpropanoid pathway, [3-6] and is controlled by PAL. Koukol and Conn reported the first plant PAL in 1961. Currently, it is known that the PAL is widely found in all higher plants, a few fungi, and a single prokaryote, *Streptomyces*, but not animals [7]. Furthermore, PAL shows potential to treat human phenylketonuria, an inborn error of phenylalanine metabolism [8]. Several studies [9-12] have shown that the PALs from *Rhodotorula* photosynthetic bacteria and monocot plants also utilise tyrosine in addition to phenylalanine; however, the dicot PALs only utilise Phe efficiently. During the past four decades, many PAL genes have been cloned and studied from various plants, such as *Ginkgo biloba*[13], *Ephedra sinica*[14], *Oryza sativa*[15], *Isatis indigotica*[16], *Arabidopsis thaliana*[17], *Jatropha curcas*[18]*,* and *Lycoris radiate*[19]*,* and the first crystal structure of a plant PAL was determined from parsley (*Petroselinum crispum*) [20]. PAL exists as a small multigene family, consisting of 2–6 members; however, some species contain additional member, such as potato (~40 copies) [21] and tomato (~26 copies) [22]. During the evolution of higher plants, the plant PAL genes diversified into various functions in each species, such as *Arabidopsis thaliana*[23]. Another important ammonia lyase, histidine ammonia-lyase (HAL), is found in prokaryotes and animals and plays roles in the general histidine degradation pathway. The crystal structure of HAL from *Pseudomonas putida* revealed its catalytic mechanism of novel polypeptide modification [24]. Despite large differences in the primary sequence of proteins, PAL functions as a tetramer, similar to HAL *in vivo*. Presumably, PAL developed from HAL when fungi and plants diverged from the other kingdoms [7,25].

*Nelumbo nucifera* (*Nelumbo*, Nelumbonaceae) (2n = 16) is a perennial aquatic plant with ornamental flowers of medicinal and phylogenetic importance. *N. nucifera* produces a series of important secondary metabolites, including alkaloids, flavonoids, steroids, triterpenoids, glycosides and polyphenols [26]. The *N. nucifera* secondary metabolites have a wide range of medical functions and also play important roles in the response to environmental stress, such as pathogen attack and ultraviolet damage. For example, it has been reported that benzylisoquinoline alkaloids and flavonoids from the leaves of *N. nucifera* are a potential candidate for HIV therapy [27]. *Nelumbo* has survived since the Late Cretaceous, along with a number of other relicts, including *Ginkgo*, *Sequoia*, *Metasequoia*, and *Liriodendron*[28]*.* It remains to be determined the mechanism by which PAL evolution has allowed *N. nucifera* to adapt to harsh environmental stress. Along with the *N. nucifera* genome project [29,30], high-throughput sequencing data will provide a foundation for identifying the key genes in metabolic pathways. However, related research for *N. nucifera* is very limited.

In this study, three intact PAL genes in *N. nucifera*, *NnPAL1*, *NnPAL2* and *NnPAL3* are identified by genome-wide analysis. *NnPAL1* is an ancient PAL member in angiosperms*.* The objective of this study is to determine the evolutionary origin, gene structure, function, and expression patterns of this gene under stress conditions.

## Results

### Genomic identification and exon/intron structure analysis of the PAL gene family in N. nucifera

**Figure 1:**
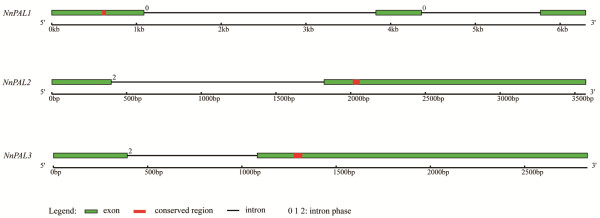
**Gene structure of the PAL family, *NnPAL1, NnPAL2 *and *NnPAL3, *in *Nelumbo nucifera.*** The green bars represent exons, and the red bars represent the conserved nucleotide sequences encoding the phenylalanine and histidine ammonia-lyase signature (GTITASGDLVPLSYIA). The black lines represent introns. The numbers 0, 1 and 2 represent the intron phase.

Based on whole genome sequences of *N. nucifera,* data mining using 4 *Arabidopsis thaliana* PAL homologues, *AtPAL1, AtPAL2, AtPAL3* and *AtPAL4*, as queries identify three intact PAL genes, *NnPAL1, NnPAL2* and *NnPAL3* (Additional file 1: Figure S1). *NnPAL1, NnPAL2*, and *NnPAL3* are located on separate virtual chromosomes, Vchr3, Vchr2 and Vchr7, respectively. According to the position of the introns, these genes are divided into the following three types: phase 0 (introns between codons), phase 2 (introns between the first and the second bases of a codon) and phase 3 (introns between the second and the third bases of a codon). *NnPAL1* has two introns of phase 0, whereas *NnPAL2* and *NnPAL3* have only one intron of phase 2 (Figure 1). In *NnPAL2* and *NnPAL3*, the exon/intron borders are within a conserved arginine codon (AG/A). The introns of *NnPAL2* and *NnPAL3* are separated by two exons. The first exon of *NnPAL2* encodes 136 amino acids, whereas the first exon of *NnPAL3* encodes 130 amino acids. However, two introns split *NnPAL1* into three exons, which code for 363, 179 and 175 amino acids, respectively (Additional file 1: Figure S1). Except for *NnPAL1*, the phase 2 intron of *NnPAL2* and *NnPAL3* is conserved, similar to other angiosperms during the evolution of angiosperms [31]. A phase 0 intron in *NnPAL1* indicates that *NnPAL1* has an evolutionary origin different from *NnPAL2* and *NnPAL3.*

Using BLASTP to search the protein database in NCBI, we found that *NnPAL1* is more similar to the PAL genes of gymnosperms (73% identity to *GbPAL*, ABU49842.1; 72% identity to *PmPAL*, ACS28225.2; and 69% identity to *EsPAL*, BAG74771.1) than dicots (63% *BnPAL*, ABC69916.1; 64% *AtPAL* NP_181241.1; and 63% *DcPAL*, BAC56977.1) (Additional file 2: Figure S2). This is contrary to the phylogeny of *N. nucifera* in the plant kingdom [32]. However, the deduced NnPAL1 protein has the same nine strictly conserved residues, Y112,L140,S204,N260,Q348,Y351,R354,F400, and Q488, that are found in PcPAL of *Petroselinum crispum*[33]. A typical phenylalanine and histidine ammonia-lyase signature (GTITASGDLVPLSYIA) also exists at position 199–214 (Additional file 3: Figure S3).

### Evolutionary analysis of NnPAL1 in N. nucifera

**Figure 2:**
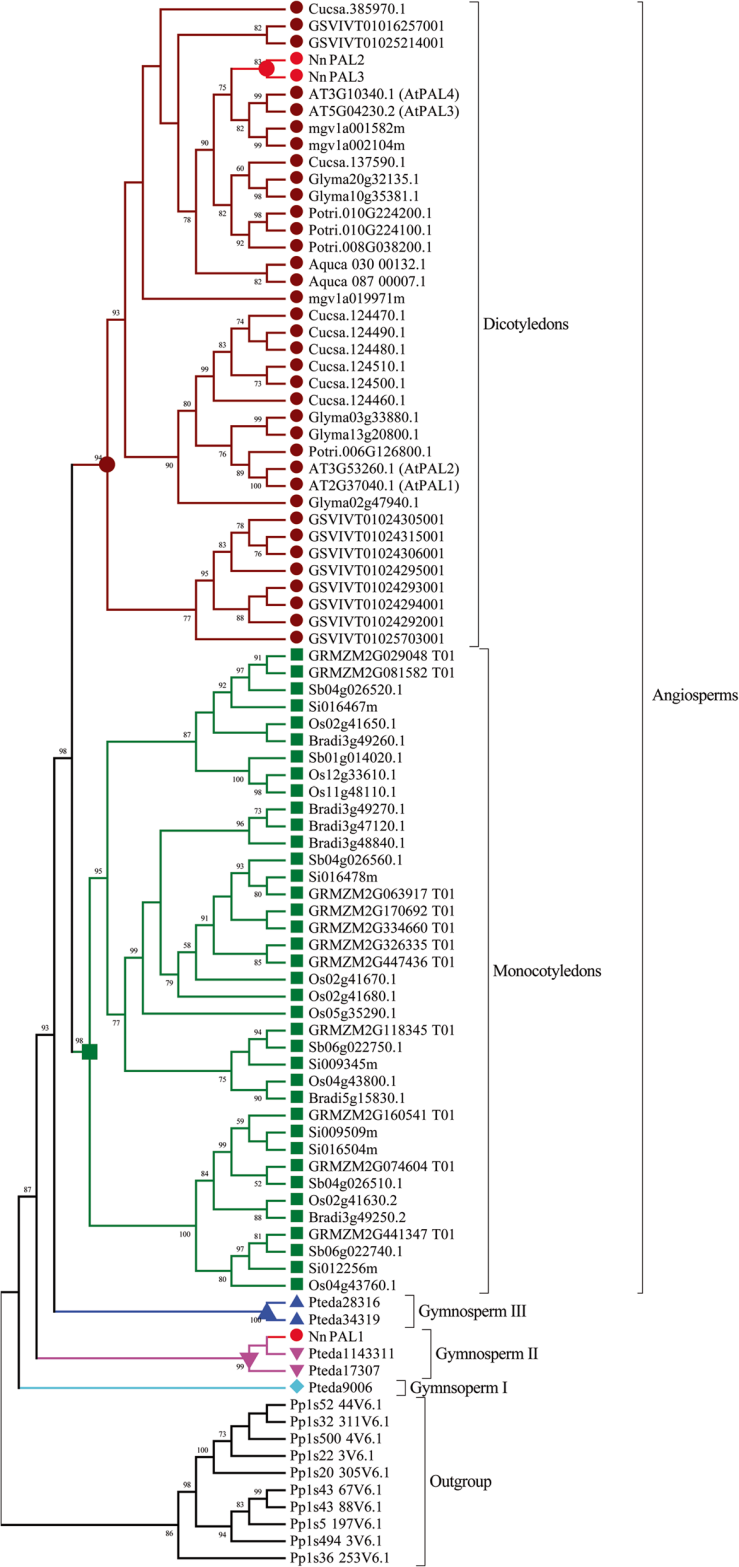
**Phylogenetic tree of the phenylalanine ammonia lyase gene family.** The amino acid sequences are aligned and the maximum likelihood tree as constructed using the program PhyML 3.0. The numbers at the nodes are the bootstrap values (>50%) from the maximum likelihood (ML). The other BI and NJ trees are shown in Additional file 5, Figure 5(A) and Figure 5(B). The numbers associated with the branches are the ML bootstrap support values and posterior probabilities. NnPAL1 is marked with a red dot, and the dicotyledon and monocotyledon clades are marked with carmine and green dots, respectively. Three clades, gymnosperm I, gymnosperm II and gymnosperm III, of *Pinus taeda* are marked with light green, pink and blue dots, respectively.

To understand the evolutionary process of *NnPAL1*, we use four PAL members, *AtPAL1, AtPAL2, AtPAL3* and *AtPAL4*, from *Arabidopsis thaliana* to query the Phytozome database. Five monocots and seven dicots that are uniformly distributed in the species tree are selected for analysis (Table 1). Intact PAL amino acids sequences from *Pinus taeda* are deduced from their transcripts (Additional file 4: Figure S4), and PAL sequences from *Physcomitrella patens* (Bryophyta) are selected as an outgroup. On the amino acid level, the PAL phylogenetic trees are constructed using the ML (Figure 2), NJ and BI methods (Additional file 5: Figure S5), simultaneously. Five different PALs from *Pinus taeda*, including *Pteda9006*, *Pteda1143311*, *Pteda17307*, *Pteda28316* and *Pteda34319*, are grouped into three clades as follows: *Pteda9006* belongs to Gymnosperm I, *Pteda1143311* and *Pteda17307* belong to Gymnosperm II, and *Pteda28316* and *Pteda34319* belong to Gymnosperm III, reported previously [34]. Except for *NnPAL1*, the other analysed PALs of the dicots and monocots, including *NnPAL2* and *NnPAL3*, are placed in separate monophyletic groups with high bootstrap values of 98 for ML and 94 for NJ and a posterior probability value 1.0 for BI (Figure 2 and Additional file 5: Figure S5). As a PAL in *N. nucifera*, the *NnPAL1* gene is clustered together with *Pteda1143311* and *Pteda17307* (Gymnosperm II) with high bootstrap and high probability values (Figure 2 and Additional file 5: Figure S5). Therefore, the PAL of angiosperms may not be derived from a single paralogue of a gymnosperm PAL. Except for *NnPAL1*, the PAL clusters from the dicots and monocots are monophyletic after the split between dicots and monocots. This phenomenon suggests that duplication events are an important occurrence during the evolution of the PAL gene family after the split between dicots and monocots [35]. However, the discovery of *NnPAL1* indicates that a different evolutionary origin may be responsible for the evolution of the angiosperm PAL genes.

**Table 1:** Identification of the PAL gene family from the Phytozome database in this study

**Species**	**PAL seq ID**
**Bryophyta**	
*Physcomitrella patens (10)*	Pp1s22_3V6.1 Pp1s32_311V6.1 Pp1s36_253V6.1 Pp1s5_197V6.1 Pp1s20_305V6.1 Pp1s494_3V6.1 Pp1s500_4V6.1 Pp1s43_88V6.1 Pp1s43_67V6.1 Pp1s52_44V6.1
**Gymnosperm**	
*Pinus taeda (5)*	Pteda1143311 Pteda17307 Pteda9006 Pteda28316 Pteda34319
**Monocotyledons**	
*Brachypodium distachyon(6)*	Bradi5g15830.1 Bradi3g48840.1 Bradi3g47120.1 Bradi3g49260.1 Bradi3g49270.1 Bradi3g49250.2
*Oryza sativa (9)*	Os02g41630.2 Os02g41650.1 Os02g41670.1 Os02g41680.1 Os04g43760.1 Os04g43800.1 Os05g35290.1 Os11g48110.1 Os12g33610.1
*Sorghum bicolor(6)*	Sb04g026520.1 Sb04g026560.1 Sb04g026510.1 Sb06g022740.1 Sb06g022750.1 Sb01g014020.1
*Setaria italica (6)*	Si016478m Si016504m Si016467m Si009345m Si009509m Si012256m
*Zea mays (11)*	GRMZM2G441347_T01 GRMZM2G118345_T01 GRMZM2G447436_T01 GRMZM2G063917_T01 GRMZM2G160541_T01 GRMZM2G081582_T01 GRMZM2G326335_T01 GRMZM2G334660_T01 GRMZM2G170692_T01 GRMZM2G074604_T01 GRMZM2G029048_T01
**Dicotyledons**	
*Nelumbo nucifera (3)*	NnPAL1 NnPAL2 NnPAL3
*Aquilegia coerulea (2)*	Aquca_030_00132.1 Aquca_087_00007.1
*Arabidopsis thaliana (4)*	AT2G37040.1(AtPAL1) AT3G53260.1 (AtPAL2) AT5G04230.2 (AtPAL3) AT3G10340.1 (AtPAL4)
*Cucumis sativus (8)*	Cucsa.124460.1 Cucsa.124480.1 Cucsa.124470.1 Cucsa.124500.1 Cucsa.124490.1 Cucsa.385970.1 Cucsa.124510.1 Cucsa.137590.1
*Glycine max (5)*	Glyma13g20800.1 Glyma03g33880.1 Glyma02g47940.1 Glyma20g32135.1 Glyma10g35381.1
*Mimulus guttatus (3)*	mgv1a001582m mgv1a019971m mgv1a002104m
*Populus trichocarpa (4)*	Potri.010G224200.1 Potri.010G224100.1 Potri.006G126800.1 Potri.008G038200.1
*Vitis vinifera (10)*	GSVIVT01024306001 GSVIVT01016257001 GSVIVT01024292001 GSVIVT01025214001 GSVIVT01024294001 GSVIVT01024305001 GSVIVT01024315001 GSVIVT01025703001 GSVIVT01024295001 GSVIVT01024293001

Note: The PAL gene family identified from one Bryophyta (*Physcomitrella patens*), one gymnosperm (*Pinus taeda*), five monocotyledons (*Brachypodium distachyon, Oryza sativa, Sorghum bicolor, Setaria italica, Zea mays*) and eight dicotyledons (*Nelumbo nucifera, Aquilegia caerulea, Arabidopsis thaliana, Cucumis sativus*, *Glycine max*, *Mimulus guttatus, Populus trichocarpa, Vitis vinifera*).

### Isolation and bioinformatics characterisation of the full-length NnPAL1 cDNA in N. nucifera

*NnPAL1* has a unique gene structure and phylogenetic position. To determine whether *NnPAL1* became a pseudogene during evolution, isolation of the full-length *NnPAL1* cDNA is performed from the transcripts of tender leaves. The partial cDNA is obtained by DOP-PCR with degenerate primers. A full-length cDNA containing an open reading frame of 2151 bp is then produced using 5′-RACE and 3′-RACE. Using BLASTN to search the whole genome sequence of *N. nucifera*, we confirm that the newly cloned ancient PAL gene exactly matched the *NnPAL1* sequence.

**Figure 3:**
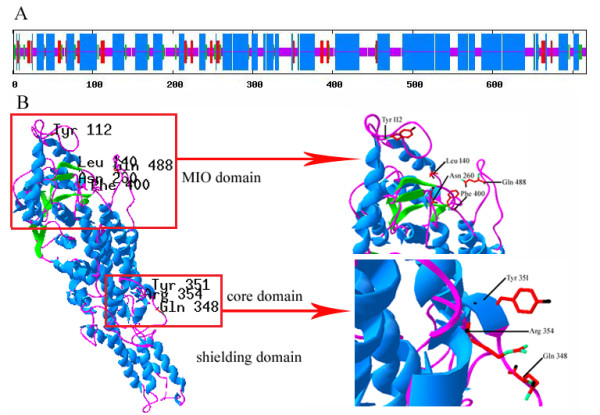
**Prediction of NnPAL1 secondary structure and tertiary structure. (A)** Prediction of the NnPAL1 secondary structure. The blue, pink, red, and green regions represent the alpha helix, random coil, extended strand, and beta turn, respectively. **(B)** The three domains of the predicted tertiary structure of NnPAL1 established by homology-based modelling (9 strictly conserved residues are marked).

Utilising the ExPASy tool (http://www.expasy.org), the resulting cDNA is determined to encode 717 amino acids with a calculated molecular mass of 77.8 kDa and a theoretical isoelectric point (pI) of 6.64. Additionally, PROSITE (http://prosite.expasy.org/) is used to identify possible posttranslational modification sites, including eight casein kinase II phosphorylation sites, ten protein kinase C phosphorylation sites, fifteen N-myristoylation sites, three N-glycosylation sites, two tyrosine kinase phosphorylation sites and one cAMP- and cGMP-dependent protein kinase phosphorylation site. The TMHMM Server 2.0 (http://www.cbs.dtu.dk/services/TMHMM-2.0/) is used to show that the deduced NnPAL1 protein is translated and located in the intracellular matrix. The SOPMA tool (http://pbil.ibcp.fr/htm/index.php) is used to predict the secondary structure of the NnPAL1 protein, and indicates that NnPAL1 predominantly consists of alpha helices (57.32%) and random coils (30.40%), along with sheets (7.11%) and beta turns (5.16%) (Figure 3A).

Based on the crystal structure of PcPAL (1 W27), the SWISS-MODEL software is used to predict the three-dimensional structure of the NnPAL1 protein. The result indicate that NnPAL1 comprises an MIO domain, core domain and shielding domain [20]. Moreover, a highly conserved Ala-Ser-Gly triad [7] that can be converted autocatalytically is also identified within NnPAL1 (Figure 3B). The results of the bioinformatics prediction and structural analysis of NnPAL1 indicate that NnPAL1 has similar structural features to the reported angiosperm PAL proteins.

### Purification and functional characterisation of recombinant NnPAL1

**Figure 4:**
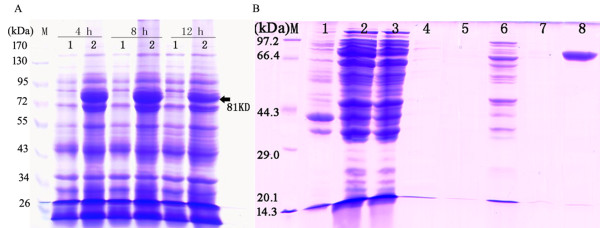
**Expression (A) and purification (B) of recombinant NnPAL1. A**: The total proteins from *E. coli* BL21 are harvested at 4 h, 8 h and 12 h after post-induction, and 1 and 2 represent the total proteins of *E .coli* BL21 harbouring the pET28a(+) vector and recombinant pET28a(+)-NnPAL1 vector, respectively. **B**: A series of imidazole buffer concentration gradients (10 mM, 50 mM, 100 mM, 200 mM); lane1: the supernatant of the *E. coli* BL21 lysate harbouring the pET28a(+) vector; lane2: (native control) the supernatant of the *E. coli* BL21 lysate harbouring the pET28a(+)-NnPAL1 vector; lane3: the supernatant of the flow through of the Ni-IDA column for three replicates; lane 4, lane 5, lane 6, lane 7 and lane 8: the products washed with 10 mM, 20 mM, 50 mM, 100 mM, and 200 mM imidazole buffer, respectively.

To confirm the expression of *NnPAL1*, the recombinant (His)_6_-tagged protein is heterogeneously produced in *E. coli* BL21 (DE3) and eluted with a series of imidazole buffers (Figure 4B)*.* The size of the expressed and purified recombinant (His)_6_-NnPAL1 protein is confirmed as ~81 kDa by SDS-PAGE (Figure 4A), which is consistent with the predicted mass of NnPAL1 (~78 kDa) combined with a His tag (~3 kDa). Compared to the production at 4 h and 12 h, the recombinant NnPAL1 (~81 kDa) is expressed maximally at 8 h. The optimal elution concentration of the imidazole buffer is 200 mM. The recombinant NnPAL1 protein has both PAL and TAL activities simultaneously, although phenylalanine ammonia-lyase from dicots only utilises Phe efficiently [33]. A study of the physicochemical properties shows that its optimal pH and temperature are pH 9.0 and 55°C, respectively. The NnPAL1 K_m_ values for L-phenylalanine and L-tyrosine are 1.07 mM and 3.43 mM, respectively.

### Expression profile of NnPAL1 under stress conditions

**Figure 5:**
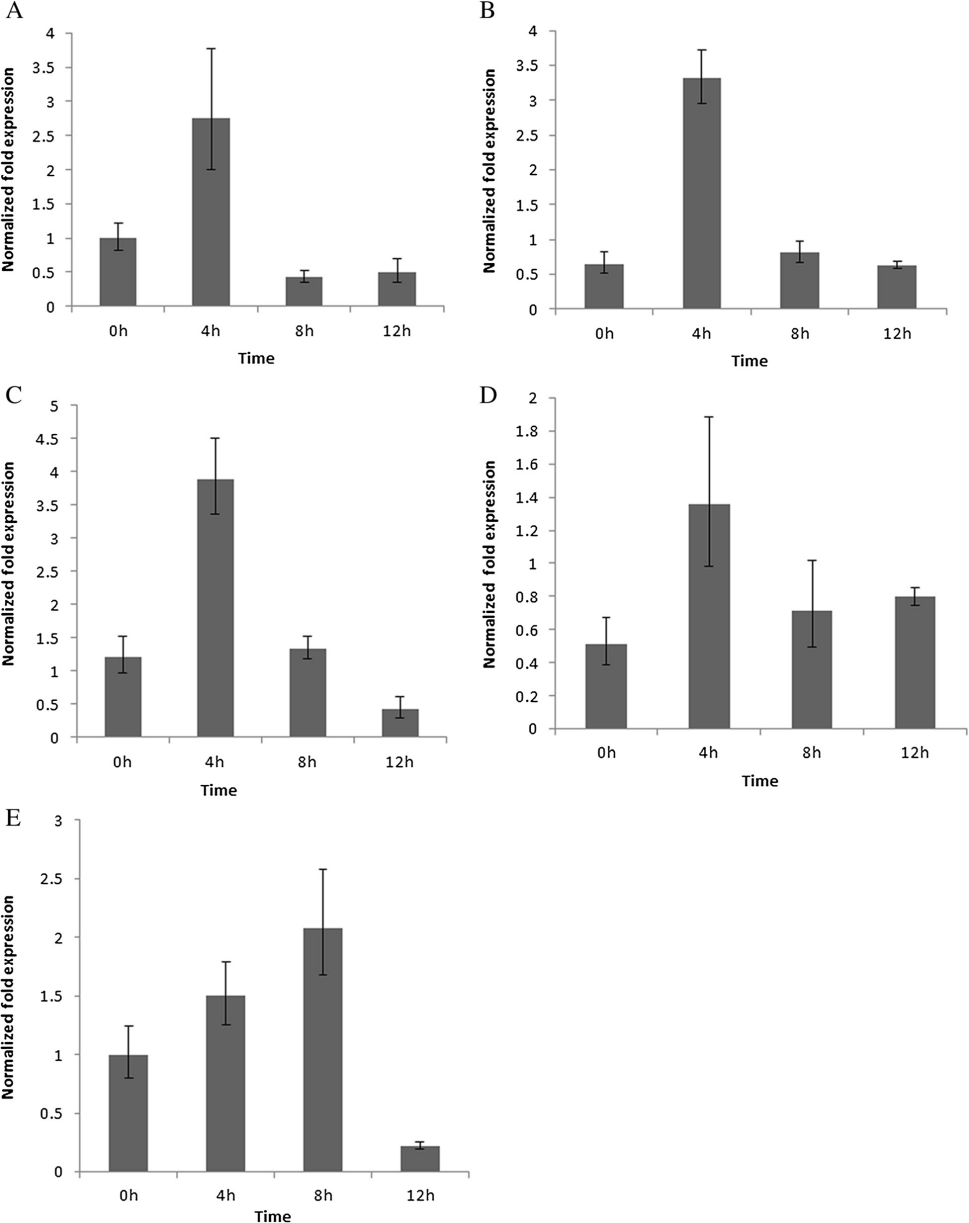
**Transcription of *NnPAL1 *under different treatments. A** 250 μM ABA, **B** 100 ng/ml IAA, **C** ultraviolet light treatment, **D***Neurospora crassa* (fungi) treatment, **E** drought treatment. The leaves obtained from the treated seedlings of *N. nucifera* are used as samples. β-actin is used as an internal control for all samples. The vertical bars represent the means ± SE (n = 3 replicates, SE < 0.5).

Because of the accumulation important secondary metabolites, such as alkaloids and flavonoids, these phenylpropanoid compounds from *N. nucifera* leaves play essential roles in stress resistance. PAL is vital to the phenylpropanoid pathway that leads to the production of these secondary metabolites. The upstream cis-elements of *NnPAL1* (Additional file 1: Figure S1), including the related regulatory elements, such as the MYB binding site involved in drought-inducibility (CAACTG), auxin-responsive element (AACGAC), fungal elicitor responsive element (TTGACC), cis-acting element involved in abscisic acid responsiveness (CACGTG), and light responsive element (CACGTG, CACGAC, CACGTG) are identified. Under different stress conditions, including ABA (250 μM abscisic acid), IAA (100 ng/ml), ultraviolet light, *Neurospora crassa* (fungi) and drought, the expression of *NnPAL1* is induced in *N. nucifera* leaves (Figure 5A-E). After 4 hours, PAL expression is maximal with ABA, IAA, ultraviolet light and *Neurospora crassa* (fungi), and after 8 hours, PAL expression is maximal with drought treatment. We conclude that these corresponding elements perform an important role in the response to internal and external environmental stimulus.

## Discussion

### Identification of the PAL family in N. nucifera from whole genomic sequences

The genomic DNA used for *de novo* sequencing is extracted from the clean shoots of *N. nucifera*. We use sixteen assembled virtual chromosomes of high quality as the resource for the PAL search. In previous reports of PAL from higher plants, all functionally identified PAL genes [13-23] encode approximately 700 amino acids and contain the characteristic conserved GTITASGDLVPLSYIA motif. Therefore, we set the sizes of the PAL family to larger than 500 amino acids with the GTITASGDLVPLSYIA signature. Three PAL genes, *NnPAL1*, *NnPAL2* and *NnPAL3*, are located in the well-defined regions of the assembled sequences. Consistent with the phylogeny of angiosperms, *NnPAL2* and *NnPAL3* are similar to the PAL from dicots. However, *NnPAL1* is similar to the PAL from gymnosperms. The full-length cDNA of *NnPAL1* is cloned from the RNA transcripts of tender leaves using RACE method. *NnPAL1* is transcribed with an intact open reading frame, suggesting that it does not become a pseudogene during evolution.

### NnPAL1 from the genuine PAL family of N. nucifera

*N. nucifera* is a perennial aquatic plant. Therefore, obtaining pure tissues is a prerequisite for molecular biology experiments. An endophyte is a bacterial or fungal microorganism, which colonises inter- and/or intracellularly inside the healthy tissues of the host plant [36]. We are careful to remove the residues from both shoots and leaves. To confirm that *NnPAL1* is a member of the PAL family of *N. nucifera* and not endophytes, we performed several experiments.

First, we determine the location of *NnPAL1* in Vchr3 and extract the upstream sequence (31,942 bps) and downstream sequence (26,288 bps) flanking *NnPAL1* (Additional file 6). Then, we performed a discontiguous megablast search against the nucleotide collection database in NCBI to search for homologous regions. In the upstream 1–5000 bps of *NnPAL1*, we find out a highly homologous region to dicots. The sequences with the first three highest scores are uncharacterised mRNA from *Vitis vinifera*, the mRNA of a tetratricopeptide repeat-containing family protein in *Populus trichocarpa*, and an mRNA of a conserved hypothetical protein in *Ricinus communis*. In the downstream 10,000-15,000 bps of *NnPAL1*, we find out a homologous partial coding sequence of the *GWD* gene for alpha-glucan water dikinase from *N. nucifera*. A homologous region to dicots is also identified in the downstream 20,000-25,000 bps. The sequences with the first three highest scores are the mRNA of a zinc finger CCCH domain-containing protein 17-like in *Citrus sinensis* and the mRNA of a zinc finger family protein in *Populus trichocarpa* (Additional file 7). As a basal dicot, the flanking sequences of *N. nucifera NnPAL1* show homology to sequences of all the other dicots.

**Figure 6:**
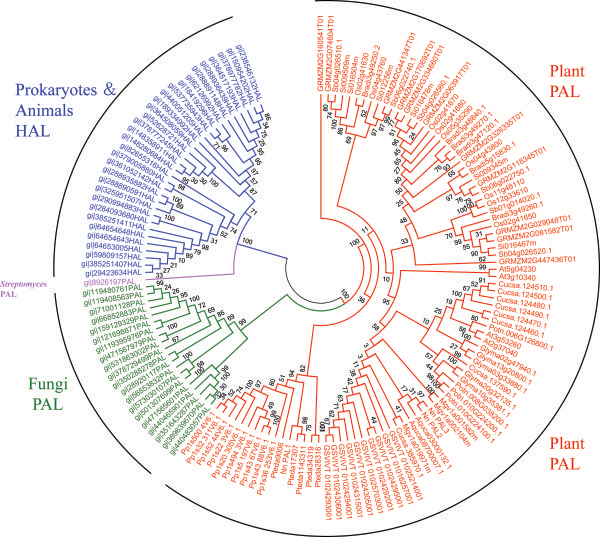
Phylogenetic tree of the phenylalanine ammonia lyase from prokaryote and animal HAL, fungal PAL and plant PAL.

Second, the identities between plant PALs and HALs and the PALs of microorganisms and animals are compared. Because PAL has the same catalytic mechanism as HAL, it is hypothesised to have developed from HAL when fungi and plants diverged from the other kingdoms. HAL is widely distributed among prokaryotes and animals. We extracted the prokaryote and animal HALs, and only one prokaryote, *Streptomyces,* and fungal PAL from NCBI the database are identified in addition to the plant PAL from our study. The identities of plant PAL to the prokaryote and animal HAL, *Streptomyces* PAL, fungal PAL, and plant PAL are approximately 18%, 18%, 25% and 64%, respectively (Additional file 8). The conclusion that there are significant sequence difference among these PALs and HALs is consistent with a previous report [20]. Similar to the other plant PALs, the sequence of *NnPAL1* is much more similar to other plant PALs, and distant from the PALs and HALs of microorganisms. Phylogenetic analysis of HAL and PAL using the neighbour-joining method demonstrates that they form three separate clades, prokaryote and animal HAL, including *Streptomyces* PAL, fungal PAL, and plant PAL (Figure 6). Therefore, *NnPAL1* is not from the endophytes. Based on these results, we can infer that *NnPAL1* is a genuine member of the PAL family from *N. nucifera*, but not endophytes*.*

### Evolution of NnPAL1 in N. nucifera during the evolution of plants

In this study, three PALs, *NnPAL1*, *NnPAL2* and *NnPAL3*, are identified using the database of whole genomic sequences as a resource. In previous reports, the angiosperm PAL had phase 2 introns at an Arg codon of [15,31,37,38], but the gymnosperm PAL had no intron [13]. Similarly, both *NnPAL2* and *NnPAL3* have only one intron of phase 2, whereas *NnPAL1* has two introns of phase 0. This result demonstrates that *NnPAL1* has unique gene structure that is different from *NnPAL2, NnPAL3* and other PAL genes from angiosperms. This difference between *NnPAL1* and other angiosperm PALs suggests that it is an ancient gene with a different evolutionary origin.

PAL and HAL are members of the lyase class I_like superfamily of enzymes, which catalyse similar beta-elimination reactions and are active as homotetramers. PAL and HAL diverged from each other when fungi and plants diverged from the other kingdoms (Figure 6). Because of their similar structures, PAL is derived from the His ammonia-lyase. HAL is a basic enzyme, participating in a central metabolic pathway, and PAL is derived from HAL to fulfil specific functions.

PAL is a ubiquitous higher-plant enzyme that catalyses the nonoxidative deamination of phenylalanine to trans-cinnamic acid. However, the origin and evolution of the PAL gene family in seed plants (Spermatophyta) have not been determined [31,39]. Currently, two major mechanisms are responsible for the evolution and functional divergence of genes. One evolutionary mechanism is called HGT (horizontal gene transfer) and refers to the movement of genes between different species [40]. HGT events occur only in plant mitochondrial genes [41-43], and rarely in nuclear genes [44]. The other evolutionary mechanism is gene duplication, which is the main mechanism for evolutionary innovations and functional divergence [45]. Based on morphological characteristics and molecular data, gymnosperms are considered ancestral to the angiosperms [39]. At least three ancestral duplication events of PAL occurred, leading to three clades of gymnosperm PAL genes, gymnosperm-I, gymnosperm-II and gymnosperm-III. It appears that angiosperms diverged from gymnosperm III when only one paralogue PAL gene is retained within the angiosperms [31]. In this study, we construct PAL phylogenetic trees that include the PAL gene families from *Pinus taeda* (gymnosperm I, II, III), monocots and dicots according to the gene sequences of the sequenced species (Figure 2 and Additional file 5: Figure S5). The phylogenetic trees show that *NnPAL1* is clustered together with *Pteda1143311* and *Pteda17307* (gymnosperm II); however, *NnPAL2* and *NnPAL3* are clustered with dicots with high bootstrap and posterior probability values. Perhaps, *NnPAL1* has a different evolutionary origin from *NnPAL2* and *NnPAL3*. Except for *NnPAL1*, the other PAL clusters are monophyletic after the split between dicots and monocots (Figure 2 and Additional file 5: Figure S5). However, the PAL from one species is clustered together with the other species rather than with a single species. This result indicates that duplication events are important in the evolution of PAL genes after the split between dicots and monocots [46,47].

During evolution, *NnPAL1* is found to be an ancient member of the PAL family that has been retained in angiosperms. A different evolutionary history for PAL genes in angiosperms suggests different mechanisms of functional regulation. In the phylogenetic trees of PAL (Figure 2), *NnPAL1* is not found where expected. Interestingly, *NnPAL1* shows high homology to *Pteda1143311* and *Pteda17307* from *Pinus taeda, and Pinus taeda* is also rich in various secondary metabolites*.* There may be a shared secondary metabolite produced by *NnPAL1* or *Pteda1143311* and *Pteda17307.* Moreover, this specific product may protect *N. nucifera* and *Pinus taeda* from similarly extreme environments. *NnPAL1* may have been essential for *N .nucifera* to survive in harsh environments during the Cretaceous period.

We speculate that the angiosperm PAL is not of monophyletic origin. Ancestral gene duplication and vertical inheritance from gymnosperms may occur during evolution from parent to offspring. In gymnosperms another paralogue of the ancient PAL exists that is retained prior to the formation of angiosperms. *NnPAL1* may be derived from the product of gymnosperm-II PAL. Discovering a functional *NnPAL1* indicates that angiosperm PAL genes are not derived from a single gene in the ancestral angiosperm genome. However, similar modification sites and structure to other angiosperm PALs suggest that *NnPAL1* can catalyse the deamination of phenylalanine to trans-cinnamic acid and is involved in the phenylpropanoid pathway.

### Functional characterisation and expression patterns of NnPAL1

During evolution, *NnPAL1* remained functional with both PAL and TAL activities. Compared with other PALs cloned from other plants [14,18], both the PAL and TAL activities of *NnPAL1* show higher K_m_ values, which can be explained as follows: (I) during the evolution of angiosperms, the function of the most ancient PAL (*NnPAL1*) is gradually replaced by a new PAL; (II) *NnPAL1* has many posttranslational modification sites, which may be involved in the subunit turnover of *NnPAL1 in vivo*[48], and prokaryote expression systems lack multiple protein modifications, which affect enzyme protein stability; and (III) the ancient *NnPAL1* has evolved a novel function required for other metabolic pathways [49]. The optimum pH is 9.0 and the optimum temperature is 55°C, which is similar to other PALs from higher plants [18]. The expression patterns are validated by real-time PCR. In response to environmental stress during the Cretaceous period, *N. nucifera* is eventually trapped in aquatic areas of Asia [50]. This type of environment makes secondary metabolites important for *N. nucifera* because they protect the species from various stimuli. PAL expression is regulated by various factors at the transcriptional level. Bioinformatics analysis of the upstream cis-elements in *NnPAL1* identified several related regulatory elements, such as the MYB binding site involved in drought-inducibility, the auxin-responsive element (IAA), the fungal elicitor responsive element, the cis-acting elements involved in abscisic acid responsiveness (ABA), and the light responsive element in *NnPAL1* gene. All the treatments used in this study cause increases of the *NnPAL1* transcripts, which suggests that *NnPAL1* is regulated by these elements. The ancient *NnPAL1* of *N. nucifera* is involved in the response to stressful environments, which makes *N. nucifera* the representative of plants that survived from the Cretaceous period [28].

## Conclusions

Using comparative genomics and phylogenetic analyses, three PAL members, *NnPAL1*, *NnPAL2* and *NnPAL3*, are identified. The distinction between *NnPAL1* and other angiosperm PALs suggests that *NnPAL1* is not derived from a PAL paralogue of a gymnosperm leading to angiosperms. We postulate that there may be another ancestral duplication event and vertical inheritance from the gymnosperms. The ancient PAL *NnPAL1* from *N. nucifera* is characterised at both the RNA and protein levels *in vitro*. The unique biochemical characteristics of *N. nucifera* may allow it to overcome the harsh environment. Additionally, as a basal dicot, *N. nucifera* is a perennial aquatic plant with agricultural, evolutionary and medicinal importance [26,27]. Polyphenolic compounds in *N. nucifera* have important pharmacological and physiological activities. The discovery and characterisation of an ancient *NnPAL1* provides new insight into PAL evolution in angiosperms and may also lead to improved function through the genetic engineering of *N. nucifera*.

## Methods

### Identification of the PAL gene family in N. nucifera

High purity DNA is extracted from clean and tender shoots of *N. nucifera*, and is used for *de novo* sequencing. For the *de novo* assembly, 16.4 Gb of filtered data with 15-fold depth is used. Sixteen virtual chromosomes (2n = 16) are assembled with high quality.

We search the orthologues in *N. nucifera* against the whole genome using four *Arabidopsis* PAL homologs, *AtPAL1, AtPAL2, AtPAL3, and AtPAL4*. The search criteria are as follows:

1) A local database with the sequences of sixteen virtual chromosomes are constructed on the Bio-Linux platform;

2) tBLASTN is conducted with a cut-off E value of 1e-20 in the local database with each member of the AtPAL family;

3) the aligned frames containing a highly conserved phenylalanine and histidine ammonia-lyase signature (GTITASGDLVPLSYIA) are selected for further analysis;

4) the related genome sequences with intact open reading frames are located in the well-defined region of assembled sequences and are extracted;

5) the extracted codes larger than 500 amino acids are selected and annotated.

### Identification of gene families in other plants and construction of the PAL phylogenetic tree

Based on a conserved phenylalanine and histidine ammonia-lyase signature, the PAL families from other plants except for *Pinus taeda* are identified and downloaded from Phytozome (http://www.phytozome.net) with a cut-off E value of 1e^−20^. The PAL proteins from *Pinus taeda* are deduced from their RNA transcripts [34]. The analysed species (Table 1) are as follows: one Bryophyta (*Physcomitrella patens*), one gymnosperm (*Pinus taeda*), five monocotyledons (*Brachypodium distachyon, Oryza sativa, Sorghum bicolor, Setaria italica, Zea mays*), and eight dicots (*Aquilegia caerulea, Arabidopsis thaliana, Cucumis sativus, Glycine max, Mimulus guttatus, Populus trichocarpa, Vitis vinifera,* and *Nelumbo nucifera*). The protein sequences are aligned with the CLUSTALW program [51] with manual adjustments. The phylogenetic trees are simultaneously inferred from the protein alignment using three methods as follows: the NJ (Neighbour-joining) tree with the JTT model, the ML (maximum likelihood) tree with LG model, and BI (Bayesian inference) tree with the GTR model, are generated with Mega 5 [52], PhyML 3.0 [53] and Mrbayes 3.2 [54], respectively. The bootstrap values are set 1000 for the neighbour-joining and maximum likelihood tree. For Bayesian inference, we sample every 10 generations for 300,000 total generations on two independent parallel runs of the Monte Carlo Markov Chains. Then, the average standard deviation of the split frequencies is calculated to check the convergence of the two runs.

### Plant material, cloning and expression vectors

*N. nucifera* mature seeds are harvested from East Lake of Wuhan, China. The tender leaves are collected when the seedling germinated from seeds in the greenhouse. *E.coli* top10 (TaKaRa, Dalian, China) is used as the host for plasmid pMD18-T vector (TaKaRa, Dalian, China) amplification. *E.coli* BL21(DE3) is selected as the host for pET-28a(+) expression vector.

### Isolation of the full-length NnPAL1 cDNA

The total RNA is isolated from the leaves using a modified CTAB method [55]. The first strand cDNA is produced by RT-PCR using reverse transcriptase (MBI Fermentas). Two degenerate primers, (Nf-F) 5′-GCNTCNGGHGAYYTDGTBCC-3′ and (Nf-R) 5′-ARNCCBARDGART TWACATC-3′, are designed according to the highly conserved regions of the plant PAL for both the amino acid and nucleotide sequences. The partial cDNA of PAL is amplified using the following conditions: initial denaturing at 94°C for 4 min, followed by 35 cycles of 94°C for 40 s, 59°C for 40 s, and 72°C for 80 s, with a final extension at 72°C for 10 min. The target fragment is checked on 1% agarose gel and purified with a Gel Extraction Kit (BioDev-Tech, Beijing, China). The purified products are then ligated into the pMD18-T Easy vector (TaKaRa, Dalian, China), transformed into competent *E. coli* Top10 and sequenced on an ABI 3730.

The full-length cDNA of the PAL gene is isolated by 3′- and 5′-RACE using the RACE Kit (TaKaRa, Dalian, China). Based on the sequenced DNA fragment, four gene-specific primers, 3′GSP1 (5′-CTGGACTACGGATTCAAGGGTG-3′), 3′GSP2 (5′-TCAGTATTTGGCAAACC CAGTCA-3′), 5′GSP1(5′-AGCATCACTTCGCAGAACATCG-3′) and 5′GSP2 (5′-GTACGGAC CTTGGAGTTGGGAC-3′), are designed for the 3′- and 5′-RACE experiments, respectively. A 860-bp fragment and 791-bp fragment are then obtained by 3′-RACE and 5′-RACE, respectively. The full-length coding cDNA of the 2154-bps is amplified and sequenced using two gene-specific primers, 5′-GAATTCATGGTTGCAGGGGCCGAGATAG-3′ and 5′-CCCTCGAGCACAAGAAGGCAACACCAAAGT-3′.

### Bioinformatics analysis of NnPAL1

The amino acid sequence and protein analysis of *NnPAL1* are performed with the ExPASy tools (http://us.expasy.org/tools) and NCBI server (http://www.ncbi.nlm.nih.gov/). The possible posttranslational modification sites are predicted by PROSITE (http://prosite.expasy.org/). The prediction of secondary structure and trans-membrane helices in the PAL protein are performed with SOPMA (http://pbil.ibcp.fr/htm/index.php) and the TMHMM Server v. 2.0 (http://www.cbs.dtu.dk/services/TMHMM-2.0/), respectively. Homology modelling is performed with Swiss-Model (http://swissmodel.expasy.org/) and is based on the PAL crystal structure from *Petroselinum crispum*[20].

### Expression of NnPAL1 in E. coli

Primers NnP1 (5′-*GAATTC*ATGGTTGCAGGGGCCGAGATAG-3′, the italics is *EcoRI* restriction site) and NnP2 (5′-CC*CTCGAG*CACAAGAAGGCAACACCAAAGT-3′, the italics is *XhoI* restriction site) are used to amplify the *NnPAL1* gene coding sequence. The PCR products are digested with *EcoRI* and *XhoI* and then inserted into pET28a(+) expression vector. The recombinant plasmid NnPAL1-pET28a(+) is transformed into the BL21 strain and sequenced to confirm the correct ORF of *NnPAL1*.

The transformant with the correct NnPAL1-pET28a(+) is selected and cultured in Luria–Bertani (LB) medium containing 50 μg/ml kanamycin at 37°C until the OD_600_ reached 0.6. Protein expression is induced with 0.5 mM isopropyl β-D-1-thiogalactopyranoside (IPTG) at 16°C for 12 h. The recombinant proteins are purified on a Ni-NTA agarose column and eluted with a step gradient of imidazole buffers (10 mM, 50 mM, 100 mM, and 200 mM). The purity of the recombinant protein is verified by SDS-PAGE. The 200 mM fractions are dialysed with Spectra/Por Membranes (MWCO: 8,000-14,000) in dialysis buffer.

### Enzyme activity assay for the recombinant NnPAL1 protein

The protein concentrations are determined with the G250 dye-binding method [56] using bovine serum albumin as the protein standard. The enzyme activity of the recombinant NnPAL1 is assayed by measuring the trans-cinnamic acid formation at 290 nm [57] and p-coumaric acid formation at 310 nm [9]. The PAL activity and TAL activity is expressed in nkat (nanomole of trans-cinnamic acid/p-coumaric acid formed per second).

To determine the optimum temperature and optimum pH for enzyme activity, several assays are performed at pH 8.5 for 30 min at varying temperatures (4, 23, 30, 37, 45, 50, 55, 60, 70, 80 and 90°C), and at 37°C for 30 min with buffer of various of pH (5, 6, 7, 7.5, 8, 8.5, 9, 10, 11), respectively. The reactions are performed in 150 μl reaction mixtures with 6 μg recombinant NnPAL1, 15 mM L-phenylalanine and 50 mM Tris–HCl (pH 8.5), and are terminated with the addition of concentrated HCl [57].

To determine the kinetic parameters and substrate specificity, 150 μl reaction mixtures containing 6 μg recombinant NnPAL1 proteins, 50 mM Tris–HCl (pH 8.5) and a range of L-phenylalanine (0.15-15 mM) or L-tyrosine (0.3-2 mM) concentrations are used. Hyperbolic plots and double reciprocal plots (Lineweaver–Burk plot) are used to calculate the K_m_ (Michaelis-Menten constant) using the Michaelis-Menten equation [35].

### Cis-regulatory element analysis and expression of NnPAL1 by quantitative real-time PCR

The 5′ upstream region of *NnPAL1* is characterised using BLASTN against the whole genomic sequence of *N. nucifera* with *NnPAL1* gene. We predicted the cis-elements by submitting 5′ fragment to PlantCARE (http://bioinformatics.psb.ugent.be/webtools/plantcare/html/).

Two-week-old leaves are treated with 250 μM abscisic acid (ABA), 100 ng/ml IAA, ultraviolet light, *Neurospora crassa* (fungi) and drought according to the cis-elements. The treated leaves are harvested and immediately frozen after 0, 4, 8, and 12 h. The total RNA is isolated from the leaves and treated with RNase-free DNase I to avoid DNA contamination. Real time RT-PCR analysis of *NnPAL1* is performed on an Applied Biosystems StepOne Plus, using *β-actin* gene (β-actin-F: 5′-CCTGATGGGCAAGTGATT-3′, β-actin-R: 5′-GCTCATACGGTCAG CAATA-3′) as an internal control for all the samples.

## Abbreviations

HAL: Histidine ammonia-lyase; PAL: Phenylalanine ammonia-lyase; TAL: Tyrosine ammonia-lyase; NnPAL1: One ancient member from PAL gene family of *Nelumbo nucifera*; DOP-PCR: Degenerate oligonucleotide primer PCR; RACE: Rapid amplification of cDNA ends; ML: Maximum-likelihood; NJ: Neighbor-joining; BI: Bayesian inference.

## Competing interests

The authors declare that they have no competing interests.

## Authors’ contributions

YD and ZHW designed the general idea and experiments of the study. ZHW characterized the sequences, carried out most of the experiments, and drafted the manuscript. STG participated biochemical assays of protein. SZW performed the expression analyses. All authors read and approved the final manuscript.

## Supplementary Material

Additional file 1: Figure S1 Nucleotide sequences of *NnPAL1, NnPAL2* and *NnPAL3,* upstream cis-elements of *NnPAL1* identified from the whole genome sequences of *Nelumbo nucifera.* Click here for file

Additional file 2: Figure S2 Sequences alignment of NnPAL1 and other typical PALs from seed plants. The phenylalanine and histidine ammonia-lyase signature (GTITASGDLVPLSYIA) is underlined with red lines, and the conserved Ala-Ser-Gly triad is framed in a red box. Click here for file

Additional file 3: Figure S3 The nucleotide sequence and deduced amino acid sequence of *NnPAL1*. The start codon (ATG) and stop codon (TAA) are underlined. The typical phenylalanine and histidine ammonia-lyase signature is boxed. Nine strictly conserved residues, Y112,L140,S204,N260,Q348,Y351,R354,F400,Q488, are marked in red italics. Click here for file

Additional file 4: Figure S4 Deduced amino acid sequences from the PALs of *Pinus taeda.* Click here for file

Additional file 5: Figure S5 Phylogenetic trees of the phenylalanine ammonia lyase gene family constructed using the BI method (a) and NJ method (b). The posterior probability and bootstrap values (>50%) for the two trees are shown on each branch, respectively. Click here for file

Additional file 6 **Identification of the upstream sequence (31,942 bps) and downstream sequence (26,288 bps) of *NnPAL1.***
Click here for file

Additional file 7 Homology search for the upstream sequence (31,942 bps) and downstream sequence (26,288 bps) against the nucleotide collection database in NCBI. Click here for file

Additional file 8 **Identities of the plant PALs to prokaryote and animal HAL, *Streptomyces *** **PAL, fungal PAL and plant PAL, marked with yellow, green, purple and blue, respectively.** Click here for file
