# Supplementary material for: Molecular evolution and functional characterisation of an ancient phenylalanine ammonia-lyase gene (NnPAL1) from Nelumbo nucifera: novel insight into the evolution of the PAL family in angiosperms
Source: BMC Evol Biol. 2014 May 9;14:100. doi: 10.1186/1471-2148-14-100 (PMC4102242; doi:10.1186/1471-2148-14-100)
Supplement: Additional file 1: Figure S1 — Nucleotide sequences of NnPAL1, NnPAL2 and NnPAL3, upstream cis-elements of NnPAL1 identified from the whole genome sequences of Nelumbo nucifera. [file 1471-2148-14-100-S1.pdf]

Figure S1. The upstream cis-clements and nucleotide sequence of *NnPAL1*, complete genome sequences and coding sequences of *NnPAL1*, *NnPAL2* and *NnPAL3* identified from whole genome sequences of *Nelumbo nucifera*. Coding region of each gene was marked with yellow colors and boundary of GT-AG in introns was present with green colors in complete sequences.

>5’ cis-elements of *NnPAL1*

CGATTATCCCCTTGTCCAGACATCAAGACTAACTTTAAAGAAAAAATATTTTAGAAGTAGT
TAAAAATGTTAGACAAATTAAAAAATATATAAAAAATTTTAGAAATAGTAATAAGATAAC
TAGGTTTGCTAAAACTTAATGCCATCAACTATTTGTTGTTAGCCTCTGATTCTTTGAAATGA
AACCCACAAAGAAACCACTAAATCCATCATTATAATTTTTTTAAATACACATTAAATTTTA
ACACAAAAAACAACATCTTACTAGTAACTAGGTTATATAGATCAATAATAGCAAATATTC
TTCATTAGAAGGAAAAAAAAAGAAACACTAAACTTTTCTTTTAAAGAAATAATATACCTT
AAGACTTCAAAACTTGACATCCCAAGACTTGGATACCTATCTTTCCATCAAAATTCAAAAA
AAGCAATTTTTTCTAGATACTGTGACAATAATCAGTCTACCTATCACTCTGATATTTGAAG
GGAATTAGTAATAATGATGTAGTCATCATCTTGTTATTTAGATCAATTATCAATACTCTTTT
TTCTTAGAGATGGTCTAAGCACTTCAGTGGTAGAGATAGAAAACTTAATTTTTTCATCTTC
CAATCAAACCAAGATTATCCAATTCATTGATAAAGCTATGATTAGATATTCCAAAGAGAA
TAGAAGAATGCATATCTATGGAATTGCTAGTTTATTTTATGTTCTTTAAGGTTTCATGTAAG
TGTAAATCTTAAAAAATTTCTCACAGATATCTTTTAGATAAGAATTTAGCTTTAAGAAGAA
AATCTTCATACTTGTTTACTAGTCTTTCTAATTTCATAACATTAAAATTTGGTTTGTCCACG
TAGAATATTATACAATTGTACACCAGGAAACCAATAGGAGGCTTTTGTGAGGAGGTTCCT
CCAAATCTTGTTCATCACAAATATCTTGCTTTCTTTTACTTTTTCTTAGAGAGTTTTAAAAC
ATGCATTCCTATAGGGTGCAGACAATATTATTATTTGCTTTTTGATTATGAACACAACAAT
GTTATAAACAAGAAAAAAAAGGAATGCAAGTTGATATCAAAGATGAGACAATATTAACC
AATTTGACCTTGGAAAATGCTACTAATTGAAACCTTATGTGAAATCTGTGGCCAATACAAG
TATGTTACATTAAGCTGCTACAACAAGATATCTAGAAGGATGTTAAGTAGCAACTAACAT
ACAAAAGTTTATCTCCAGAATCATGAATGAGAGTTGGGTTTTGTGTAAATGAGTGTAGGTC
AATTGTCACGCAAAAGTTTATAGATACAAAATCCAACTTGTTTAATTTGTTGTAGTGATCG
CAAGTATATCAATAACATAGTAGAAAAGAAAGGCTTGTATAAGACTTGTTGGCCTAAATT
AAAATAAACAAATAACATCTCAATAACTTAAAAACACAAATATGAAAAACAACTGACTAT
GAAAAATACACAAATAGCAAAAGAATCCTAAAGAGGGGATCTAATCCTGGGCCTCACCAT
CTCAATAACTTAAAAATTAAAACAAGTATGAATATTTAGGGGTAGACGTGTAATGCCTAT
CAACTCCTTAAAACCAATATTTGTTATCCCCTATTTTTTGTTAGGAGATCATCATCACTACA
TGAATCTTGTTTTTCCATCCATTTGTGGCTAACTTACTATTAGACATGCTAATAAAGTCACT
CACCTCTTCAAATAACTGATACTTAAAGAGGTAGGGAGAAGCGGATGAGACAACAGTCAA
AACACTGGTCCACCACCCACTGGGGCTGAACTACTAGCGCTACTGCTTGCAAGTCATGGG
GAATTAAGTGAGGGAAGGAGAAATGGAGAATGTGAATAGAGGAGAGTCTCGACTCTAGA
TTTTGGAAGTCGGAATTGCTATTGCTACTTGCAATTTCAAATTCGTCGGACAGTGCTTGCT
GAGTTTAGAGGGTTGATTCATATTTCAAATTTGCTGATTTGGCCATTTAGGAGTTTCATTAT
CATTGTCAAAGAGGAAGAAAGGGATTATGGGAAGGAAAGGGTTTTGCCACTTTTTGGGTT
TTGGCTTTTGGAATTTAGATTGAATTGGGGGAAATGTCGACGTCTGGAAACTAAAAGACA
AAAGTCCAAAATCTAAGACTAAGAGTCATGTGCCAAGCCATCACTCATCACCTATAACAT
TTAAAGGGTAATGATGTAATTTACCATTGGGTGAGGCTGGTTTAGGGATTTATATGTAGAC

CCCATCCCTAGTTCAAGGTAAAAAAATCCTTGAACCTGACCCATCCCCATTTAAGGTCGAG
GAAAATCCACCCCGTTCTGGGCACATTGGATCGGGGCCTCAATTTAGTGGGGAAAAGTGT
CATCCCTAGTTTTAAATTGTTGCCATTTATGGAGAAGACAAATAATTATTGTCGTTGTTGG
GGAGGAAAACAAGTTAAAATCAATAATTTTAGGAAATCAATTGAACCATGACCCAAACTT
ATTTCCAACTCAGTTGTACCCTATAAATTGGATTGCATAGAGAACTGCTTAATACCAGATG
ACCCATCAGTTTAACCAAAAAATCGATGAATTGATCTTAGTTTCTTAAAACAACTCGAGTC
TTAAATATATCAAATTTTCAAAATGCAAGTAATAAGATTTGAACTAGACCTGTTCATAGAC
CGGGAAGCCCGACAGGCTATCCCAGCTCTACCTGTTATTAAGTCGAGCTTGGTCATAATTT
TAGGCCTGTAGATTGGGCCCAACTTTTTTTTGGGTCGAGCGGCCTAGGCCTGAGCTCGACT
TGATACTATATACTAAATGATAAAAATAAAATATATATATATATATATATATATATGTATA
TATATAATATTTTTTTCTTTGTTCTTCTTCTCTTTTTTCCAAGTAATAGTGAGATGTTTATTA
TTTTTGAGTTTTGAAGGTTTTTAATATAAGTAAAACACTTTTTTATTGAATAAAAAAGAAA
AAAAATTCTATTTTCAAATTTCTGGTTGGGCTCGGGCCCGGGCTTGGGCCCGAGTGTATTC
TTTTGATACATATACAAAGCCCAGCCCGACCTGACCAATGAACAGGTCTAATTTAAACCC
ATGACCTTCATTCATGTATATTGACTTTTTACCATTAATCCCCTTCATTGTTTACGTTCACTT
ATAAATATTTAATGCATGTACTACTTTAATAAATATGTATTTTAAAGAAGATGGAAGTTAT
GAGGTTTGATCCCTAGTCAATTGCGCTAAAACTTAAAGAACTAACCATCTATCCACCACAT
TTAGGTGCAATAATACCTTCGCTTTATAGTTAAACCTCTAATCCATGAACCTCTCCCTTTTT
TGGTTATATGTTCAATTCAATTCTTTGAACACTCATTTTGTTTTTAAAACAAATGTCGCAAT
CACACTCACTTTTGTATGTTGTGGTGGTAGAATAGTGGACTACATAGGATAATGATTTGGC
CAAGGAATTGCCTGCATTTAATCCTAATACAACATGCTTGAGATTGTTGGGTCTTGAGAAG
CTTAAATGAAAATAGGGAAGAACTTTTAAGTTTTAAAAGTTTTAAAGAAAAGAGTAAAAT
GGTCTTAAAAATAATGAAAAAGATAGTTATAAAGTTTTAAGTAGAGGTGTAAATGGATTG
AATTTGAATCGAATTAGGCAATATCCGAATTCGAATCCGATTAGAAAATTCATTATCCAAA
TTTGGTTTGGATTCAACCAATTAACTATTTTAGGTAATTAATAAATATATAAAATAAATAT
TTTATTTATATAATTTAACTATTATATATATATATATGTTATATAAAATATTTAAATATTTT
ATATATCATATAAAAGAATTAAATAATCGACCGAATTCAGATATTTATCGAGTCGAATCA
GGTTAGATCCGAATTCAAATACGATTAACTATTCGGTATGGATAAGGTATCCGAATTTGAT
CGATTATATATTTAAATTATCCAAATTCGATTTGATTATGTTGAATCTCGAATTTGAAATTA
GATTTTTGAATACCATTGACACCCCTAGTTTTAATATGCAATTTACTAATATACATCTTGCC
ATGATTTTCCATGCAAAATGACATTCTAAGGGCTTGTTCATTTGCTCTAAAACCTGACATC
TGCAAGTATAATAATTACATTAAGAAGTATATTGGCATTGATGAACTTTTCTTTTCTCTAA
ACTCTGAATGCAGAGAATATTGTTCTGAATTTTGAAAATTATCAATTATCTATTTTGACCC
CATATATGAGAGTTTTTATTTATCTTTTTTAGAGTATGGTTGAAAAATAAAAAATTGTATG
ATTGAACTTAAAAAAATAAGAGATAAAAAATCGGACAACCATTTTTCTCTTAAAATTTAA
AGATTTCTATTAAGATATAAAATTTAAAAAGGAACGAACAGGCACCAACTTCTAACTCAA
TAATCTCAAACACATGTTTCACTTTCGTCGCTGGTTTTGGCGTTTATTCTTGAAAATGCAAT
TGTTACCTACCTATCAAAAACCAAAGCCGCACATAATTAAGATGCTAGACACCCTACACA
CCTTGCTATTAACTATTACCTATTAGCTAGGAAGAGATAGGTAAGCACATGCGAAGCACG
AAGTAGTTGGAATATGTGGAGAGGGAGGGGGAAGGGCTGTTGAACAGTTGGAGTTGGAA
GTTGTAAGTGTTGAACGCACGATGACGGCTGCGACAACCACCGCTACTCTCACGTGCGAA
CCCAATCTCCCACTCTCAACGACCAAACCGTGGTGGAAGGTAGAGAACCAAACGCTCAGA
TCTCCCCACGACACACCCTCCCTTCAACCCATCCTCCTTCCTTTCGTGGCATTTCCTCTCAC
CGCACACACACACACACTTTCTCTCATTCTCTATTAAACCCGTCTCTTCTTCTACCTCTTTA

CCCAGTTCAATCCCTCAATTTCCGGTCCCCCGCTCTCTCTCTCTGTCTCGAACCTCTTGC
ACGCGTCACC

>*NnPAL1* complete sequence

ATGGTTGCAGGGGCCGAGATAGTGCAGAATGGCTCGCACTCGCAGATCAAAGTCCTTC
CATGCAGCATGTGCGAGGAGGATCCTTTGAACTGGGCCAAGGTGGCCAAGGAGCTCC
AAGGTTCGCACTATGAAGAAGTGAAGTGCATGATCGATCGATTCAGTCGAACCAACTC
AGTCAACTTGCAAGGTCAGAACCTCCAAGTCGCAGACGTCGTCGCCGTCGCTCGTCG
CCAAGCCGACGTCGAAGTCCGACTCGACGCCGACACTGCCAAGTTCCGGGTGCAAGA
GAGCGCTGCCTGGGTCTCGGCTCAGTCCTGCAAAGGTACCGACACTTATGGCGTCACA
ACCGGTTTCGGCGCCACCTCGCACCGCCGGACGAACCAAGGGGTTGATCTTCAGCGG
GAGCTTATCAGATTCTTAAACGCCGGCGTCATTGCCGGAGACGGAAATGAGCTCCCCG
GCGATGTTGCCCGAGCAGCCATGCTTGTACGTACCAACACTCTTCTTCAAGGCTACTCG
GGCATCAGATGGGACATACTTAGTACCGTCAAGGACCTCCTCAACGCTGGCCTGACAC
CGTTACTCCCCCTCCGCGGCACAATCACAGCCTCCGGCGACCTCGTCCCGTTGTCCTAC
ATTGCAGGAGTAATCACCGGGCGTCCCAACTCCAAGGTCCGTACATGCACAGGCGAGC
TGATCTCCGGAGCGGAAGCTCTCCGGCGCGTGGGAGTGGAGAAGCCATTCGAGTTAC
AACCAAAGGAGGGGTTAGCCATAGTCAACGGAACCGCAGTGGGAGCAGCGCTGGGG
GCTATCGTGTGTTACGACGCTAACGTCCTGGCGGTGGCGTCTGAGATCGCATCGGCGAT
GTTCTGCGAAGTGATGCTGGGGAAGCCGGAGTTCACGGATCCGTTGACTCACCGGCTG
AAGCACCATCCGGGTCAGATGGAGGCGGCGGCAATGATGGAGTACGTTCTCGCCGGA
AGCGGCTTAGTTAAGAATGCGGCAAAGCTTCACGAATACAATCCCTTGCAGAAGCCGA
AACAAGACCGATATGCTCTTCGTACTTCTCCCCAGTGGCTGGGCCCACAGGTATGTTCA
CTCTATACTTAATCTCTCGATCGGACGGTTGAAAGAGGTCGGCTGTCGATCGATACATG
CACATGGTGCACGGATGTGTAGGTTGTAGAAACGTTTTCGAGCCGCCGTTGACCCGGA
CGGTCGGACCGTTCGGCCCGAGAACCGTCCAGGCAATCGGTTCTATTTTACCCAAAAC
CCGAAATCTGGTCAAAAATCATTGAAAAAGTCAATAAACCGTTGACTTGGGTGGTCAA
ACCATTGATCTTTCAACGGTTTTGGACCATTTTTTTAATTACTTTTAATTTTGCTGTTAGA
ATAGGTCATCTGTTCGAACTGGACGATCAGACCATTGACCCTGAAATCGGTGTTCAAAA
TGGTTCCGTTTCCGAGTCATTTTTAAAAATGTTGGGTTGTAGTTATATCAGATAATCATA
CGACGAACGTGACCATAAATGAAAGAGGGGCCCGCTTAAGATTATGGAAAGCTGGAG
AAATCAATATTTTAAAAACTAACCCGGAACCAGATTTTGAACAAATACCATGTTTGAGG
TAAAATAGAATCAGATGCTTAGACGATTTTCGGTCCGAATGATCTGACTATCTGGATCA
ACAGCGGTTCGAAAACATTGGGCGAGATGCTTAAGGTTAATACATATTTATTACATTAAT
ACATACTATCAAAGTCAATCTATTTAATTAAACTTGAAAAGTTATATAATAAATAGGAGC
TAAAATTCGGATAATTTGCTCGTGGCTAATCAAATCAATTGCACTTATTATCCATGTGGT
GGCAGTTGAATAATTGACTTATTATCCATAATTATAATTTCTTAAACAAATTAAAATTCTT
TTAAAAAACAAAAATTAGTAAAAGAAATGAAAATATTAGATAGAAAATAATAAAAAAC
GTTTATAAAAGTAAAAATTGTAAAAGATAAAAAAGAATAACAAAAAAAACTTCACTCC
TCTCTAGATTTGTAGCATGATGAAAATTGTTATATTTTTTTAGTATTAATCACTACTTGAC
ACCCCTTACGGGTCATTACTCTTTTAGCTAAACAATACAACATTACATTAATATCTCTTTT
CTTTGATAATAGTGGGGATTCAAAGTCCCACCATCGATGCGACAATTTATCTACCGCAC
CTAATGTTTTTGAACCGTTGTTGATTTAGATGATTGGACTATTTAACCTAGGAATCGTTC
AAGTAACTAGTTTTGTTTTACTCCAAACCCAGTTCCAGGCCAAAAATCGTTGAACTGGT
TAATTTATTGAGTTAAGTGGTCGAACCACTGACTCGTTTGACCCATTCAATGGTTTATGG

TCATTTTTTATTTTAATTTATTTAATTTTTTGTTAAAATGGGCTATCTATTCGAACCATTGA
CCCAAAAACCAATGGCCAAATCAATTCTAGTTCTAAGTCGATTTCAGCTGATAATGAAA
ATTGTTATATGAGCTCATGGATTAGAGTTGTCCAAGAATTTGGAAAGTCCAATATGATTT
CAAATCCAATCTCTTTACGAGTTACAACCCTAAATTGAGTGTTAGATATTGATAATTGAA
TTGGACTTAGATACTAAGGTGACTCCTATCCAAATCAGCCCTAACTAGGGGTGGATTTG
AGCCGAGCCAAGCCGAATACTTGGCTATTCGGGCTCGACTCGCTGAAATTGTATTAGGC
TCGAGCTCGATTCGAGCTAGAGACTTGAGCTCAAGCTCGACTCGGTTCAAAGCTCGCA
AGTATGAGCTCGACTACAAGCCAAGTTTTTTTTTAAAACTCGTCTCTTGTTTAATTTGT
AAGCCTAGACAGGTTAATGATCCGTACTTGGCGAAGCAGAATTGATGTAGCCGATTACT
TTGAGTTGGGATAAGGCTTACAAGATGATGTTTGTTCTGAGCCCAAAACTGTGCAATCG
ATTATTACCATTCCAATGGGTTTAATGGCAAATGGGAAAAATGTTTCGTTGTAAAGAAA
CCGGGTTCAGTCGGTGCAAAGCCCGTTCTAACACTTATTATAGGCTTGCATTGAAAATG
TATATTTATGGGAAGAATAATACAATAATTTTATAAATTAAAGAAAAGATAAATTAAAAA
AAAAATATTATATATAAGATATTATTATATAAATAATAAAAATTATTATTATATATATACATAT
ATATTTATATGTATATGTATGCATATAATAAAAAATATTTATTTATTATAGTAGGCTCGATTA
GGTTCGCGAGCCTCACGAGCCGAGCTTCACTGTGCTCGAGCTCAGCTCGATTAGCAAA
TAAGCCTATATATCAGGCTCGAACTCGGCTCGAAGCTTGGTTTAAACCGAGCTAAGCCG
AACTCAAATAGCTCGTGAGAAGCTCAGTTTGTGGCCGCCCCTAGACCAAACCCTAGGT
TTAGAGCATGGTTTTGAAAATTAGACCGAAAATGGAACCACTTTAACCATTGATTCTTG
GGTCAAATGGTTCGACCATCTGATCAGAACCTTGGTCGGATGGTTTTGGGGTCTAAAG
CTGAATCCGGAGCCATGCCCCTTCTCAAGTCAAATGGTCCGACCATCCATTCCGGTCCG
ACTTTCCAAAGCATGGTTTAGAGTTTGTAACAAGAGATATAGTGGGTCAGACTCAGAG
GTAATCGATGGGACAAGCCGGGCAAGGAAAGGTCAATCTGTTTGCAACGGGTTGGTG
GGTAGGGTAGAGTCAAATATTGTGAACCAAAGTAACGTTGATTACGTTGGATGCAGATC
GAGGTGATTAGAGTGGCCACCCACATGATTCAACGTGAAATAAACTCAGTGAATGACA
ACCCGGTTATTGATGTGGCCCGAGACAAAGCCCTCCACGGTGGCAACTTCCAGGGGAC
CCCAGTCGGTGTAGCGATGGACAATCTACGTCTAGCCGTGGCGGCGATCGGAAAGCTG
ATGTTCGCGCAATTCTCTGAGCTAGTGAACGACTACTACAACGGAGGCCTGCCTTCCA
ACCTCAGCGGCGGACCCGACCCCAGCCTGGACTACGGATTCAAGGGTGCTGAGATTGC
CATGGCATCATACACGTCAGAGCTTCAGTATTTGGCAAACCCAGTCACAACCCATGTAC
AGAGTGCCGAGCAACACAACCAGGATGTTAACTCTCTCGGCTTGGTATCCGCCCGGAA
GTCGGCGGAGGCTATCCACATCCTCAAGTTGATGACTGCAACCTACCTGGCCGCGCTC
TGCCAAGCCATTGATCTCCGCCATCTTGAGGAGAATCTCCGCCAGACCGTCAAATCCGT
TGTTGCACAGGTGTGTATTCTAGTCAGCCACTGACATAGCTACACTAACAGTTATTTTT
ATTTGACTTTTCTTTTTTAAGAGTATATACAATTTAATTCATTAAAATATGGGCCGCTGTT
TCTGGGGTTCAACAACCTTGGGCCTAGTTTTTCTTTTTTTGTTCAGGTCCAGCATAGGC
CCACAAATTTATGGCCGCGCCTGGGCCTAATACTCCAGGCCCAACCTGGCCCATGTTAT
AAAATATTTATATATGATTTTTTATGTTGCATTATATAGTCTTATATATATAATCTGTGATATG
ATATGCATAATTCATATTTTATATATTTGATGTATTTATAGATAAAAACACGGGCCCGCAG
CTTACTGTTTCAAGGCCTAAAACCCACACTGGGCTGGTCTGGGGCCCAGGCTATAGCC
TTAACCAAAGGTACAAAGCCTGGCCTATGATCACCCCTGGCTTTTTCTAAATCAATTGG
GTTGGTCCAAAGTTCAAAATATTGTTATAGCCGATATGCATTGTATCGAGGCCTTCTCTA
TGAGACCAAATCAGTCCCCCCACAACATCCCATAGCACATTTTCACCGTGTATTGAAAA
CATCTCTCAATGATAACCTCAGAAATGATGATGTCATCATTAGGAGATGCTCTCAATATA

CAACAGAAACGTGTTGTGGGGAGCAATCTTGTGGGGGGCTGATTTGAACTCCTCCTCT
ATATGCCTCTATAGGGGATGTATCGGGTCAGGCCATGGTTTAGAGGATAGTCAGGACTT
AAACCAACCCTTTTGAATCGGTTTTGAAAAAAAATAAATAAAACACAAAAAAAGCACT
GATACAAGCTGATACACCTTTAACGGCGTATCGGCCTGTATTGATATTTAGAACCTTGGG
TTGGTCTAATGAGTAAGATATGGAAAAATCTATGTTGTCGAGAAGAGAATTCTAAAAGA
GTTTTGGCCAATCAACACCACCATACTGCCTGATTAGTTACCAATTCCTGGTTGATTGGT
TGCAAGCTTTTCTACTATCATGTCTTTTTGGTATCATAGTTGAAATCTTGCAATTTTTCAA
TTTTTTTTTTCCTTTTTGTGGCAAGGTCCATACCCAGTCTGGATCTCTTAGTGGCAAATC
CTTCCCAAATTCCCTACCCATAAAACACCACCCCTGTGTATTGAGAATAATCTGATATTC
CCAACAATGTCGTGTGTGTAAAATTGTGGGAACAGCTTGTGGGTATCAGATCCAAGCG
CGTCCCATGCAAAACACTCACAAAATTGTGGTATGATTTTTTTGAGAAAAAAAGTGCA
GCCGCTCAATTCAATTAATTTCTCATGTACCATACCAAACGCCAACTAATTGGGCTTATA
TTATTATATGTATCTTAGCAACTAAAACTCATTGATATTCCATGGCACAGGTAGCAAAGA
AGACCCTAAGCACAGGACCCAACGGTGAGCCGCTCCCTGGCCGATTTGTTGAGAAAG
ACCTGCTCCAAGTAGTGGAGAGCGAACCAGTATTTGCCTATGTGGACGACCCTTGCCG
TGTCGACTACCCTCTCATGCAGAAGCTCCGGCATGTCCTCGTTGAACACTCACTACAG
AGCTCGCATACAGAGGCGGAGCTGTCGCCCAAATCTGGTGTCTTCGGACGGATAAAGA
TGTTCGAATCCGAGCTCAAAGCGCAGCTCAATGCCCAAGTTAAAATTGCGCGTGCCAA
ATATGATAACGGAACCCCACAGGTTCCCAACAGGATCGCCGATTGCCGGTCGTATCCGG
TCTACAAGTTTGTCCGTACAGAGCTTGGTACTCAGCTGCTTAGTGGCACCAGAAAAGT
GTCTCCTGGGGAACAAATCGAGGCCGTCCATGCGGCTATCTGCGATGGCAAACTGGTT
GCTCCATTGATGGAGTGCTTGAATGGATGGCCCCAGAGGCCTGGGCCATTTTAA

>*NnPAL1* coding sequence
ATGGTTGCAGGGGCCGAGATAGTGCAGAATGGCTCGCACTCGCAGATCAAAGTCCTTC
CATGCAGCATGTGCGAGGAGGATCCTTTGAACTGGGCCAAGGTGGCCAAGGAGCTCC
AAGGTTCGCACTATGAAGAAGTGAAGTGCATGATCGATCGATTCAGTCGAACCAACTC
AGTCAACTTGCAAGGTCAGAACCTCCAAGTCGCAGACGTCGTCGCCGTCGCTCGTCG
CCAAGCCGACGTCGAAGTCCGACTCGACGCCGACACTGCCAAGTTCCGGGTGCAAGA
GAGCGCTGCCTGGGTCTCGGCTCAGTCCTGCAAAGGTACCGACACTTATGGCGTCACA
ACCGGTTTCGGCGCCACCTCGCACCGCCGGACGAACCAAGGGGTTGATCTTCAGCGG
GAGCTTATCAGATTCTTAAACGCCGGCGTCATTGCCGGAGACGGAAATGAGCTCCCCG
GCGATGTTGCCCGAGCAGCCATGCTTGTACGTACCAACACTCTTCTTCAAGGCTACTCG
GGCATCAGATGGGACATACTTAGTACCGTCAAGGACCTCCTCAACGCTGGCCTGACAC
CGTTACTCCCCCTCCGCGGCACAATCACAGCCTCCGGCGACCTCGTCCCGTTGTCCTAC
ATTGCAGGAGTAATCACCGGGCGTCCCAACTCCAAGGTCCGTACATGCACAGGCGAGC
TGATCTCCGGAGCGGAAGCTCTCCGGCGCGTGGGAGTGGAAAAGCCATTCGAGTTAC
AACCAAAGGAGGGGTTAGCCATAGTCAACGGAACCGCAGTGGGAGCAGCGCTGGGG
GCTATCGTGTGTTACGACGCTAACGTCCTGGCGGTGGCGTCTGAGATCGCATCGGCGAT
GTTCTGCGAAGTGATGCTGGGGAAGCCGGAGTTCACGGATCCGTTGACTCACCGGCTG
AAGCACCATCCGGGTCAGATGGAGGCGGCGGCAATGATGGAGTACGTTCTCGCCGGA
AGCGGCTTAGTTAAGAATGCGGCAAAGCTTCACGAATACAATCCCTTGCAGAAGCCGA
AACAAGACCGATATGCTCTTCGTACTTCTCCCCAGTGGCTGGGCCCACAGATCGAGGT
GATTAGAGTGGCCACCCACATGATTCAACGTGAAATAAACTCAGTGAATGACAACCCG

GTTATTGATGTGGCCCGAGACAAAGCCCTCCACGGTGGCAACTTCCAGGGGACCCCAG
TCGGTGTAGCGATGGACAATCTACGTCTAGCCGTGGCGGCGATCGGAAAGCTGATGTT
CGCGCAATTCTCTGAGCTAGTGAACGACTACTACAACGGAGGCCTGCCTTCCAACCTC
AGCGGCGGACCCGACCCCAGCCTGGACTACGGATTCAAGGGTGCTGAGATTGCCATGG
CATCATACACGTCAGAGCTTCAGTATTTGGCAAACCCAGTCACAACCCATGTACAGAGT
GCCGAGCAACACAACCAGGATGTTAACTCTCTCGGCTTGGTATCCGCCCGGAAGTCGG
CGGAGGCTATCCACATCCTCAAGTTGATGACTGCAACCTACCTGGCCGCGCTCTGCCA
AGCCATTGATCTCCGCCATCTTGAGGAGAATCTCCGCCAGACCGTCAAATCCGTTGTTG
CACAGGTAGCAAAGAAGACCCTAAGCACAGGACCCAACGGTGAGCCGCTCCCTGGCC
GATTTGTTGAGAAAGACCTGCTCCAAGTAGTGGAGAGCGAACCAGTATTTGCCTATGT
GGACGACCCTTGCCGTGTCGACTACCCTCTCATGCAGAAGCTCCGGCATGTCCTCGTT
GAACACTCACTACAGAGCTCGCATACAGAGGCGGAGCTGTCGCCCAAATCTGGTGTCT
TCGGACGGATAAAGATGTTCGAATCCGAGCTCAAAGCGCAGCTCAATGCCCAAGTTAA
AATTGCGCGTGCCAAATATGATAACGGAACCCCACAGGTTCCCAACAGGATCGCCGAT
TGCCGGTCGTATCCGGTCTACAAGTTTGTCCGTACAGAGCTTGGTACTCAGCTGCTTAG
TGGCACCAGAAAAGTGTCTCCTGGGGAACAAATCGAGGCCGTCCATGCGGCTATCTGC
GATGGCAAACTGGTTGCTCCATTGATGGAGTGCTTGAATGGATGGCCCCAGAGGCCTG
GGCCATTTTAA

>*NnPAL2* complete sequence
ATGGAATTCGCCCACGAGAACTGCAATGGCAACGGCAACGGCAGCATCAAAAGCTTC
TGCGTCCAACAAGACCCACTGAACTGGCAAATGGCCGCCGAGTCTCTCAAGGGAAGC
CACCTGGATGAGGTTAAGCGCATGGTGCAAGAGTACCGGAAGCCGGTGGTCAAGCTA
GGCGGCGAGACCCTTACCATCTCCCAAGTGGCGGCCATCGCCACTCACGACGCCGGTG
TCAAGGTGGAGCTGGCGGAGTCGGCAAGGGCCGGAGTGAAAGCGAGCAGCGACTGG
GTCATGGAGAGCATGAACAAGGGCACGGACAGTTATGGTGTCACGACCGGTTTCGGTG
CAACTTCACACAGAAGAACCAAACAAGGAGGAGCCCTTCAGAAAGAGCTTATTAGGT
AAATACATTAATCATCCCCACTTTCCCCAGATTTATCATTTCTTAATGAATTGAACCATTT
TAATTCTTGGTAGAGGGGATTAAGTGGTTGGCCACCGATTTTCCCCTGTTCCAACGTAT
TTGGGCTGAGGCGTTTAATATATTGGTTGAGCGGCCTGCTGAGCTGACAGAATTTTTAC
CTGGTGGTTCGTTCAGGGGATACTAGCCTAATCCTGCTACCTTTACGAAAGGTGGTGGT
AGTGGTAGCCTGGTGGGTACTAGATAGTTGTAACTTGTGGGCCAGGAAATACCCGACA
ACGGGTAGGAAAAGAGTCGGAGCTATTTTCATATACCCGAAGCGGGTCTGGGTTTGGA
CCACGAAATTGACCACTGTGTGTGTACAATTTATCTTAATTATCCATCAAAGGGTTATGC
CCCTGATCCATTACTGGACTCCAGATCTCTACCCACCACGGGAATATCTCTCCATGCATA
ATAACATACAAGCATAATAGGAGATGCTCTGTATGTGTGGTTGTGAAATCATGTGGGTA
AGTGTGGATAGTGAATCCGAACTGCCATTAATGGGTTAATGAAAATGTAAAATTATTAA
ATTTTCCATTTAAGATTTTATGAGTTGAGAGCAATAAATATTAAAAGGGCAAATTTCTTA
AATTATCTTTATTGTTTAGTTAATTGATAAATTCTACCTTTACTAGAAGTTTTTAAGATATA
TAAACTCTATTGAAAAAAAGTTGAGAAAAATATGTGAGAGTTTAGGACTTTAGAAAAA
AAACTGAGTTTAAGGGGCAAAATGGTCTCAATAAGGGTAGTTTGGGAAAATATTTCAT
GAAGTTAAATAACGGAATAGTATGATAAAATGTTGGAATCCAAAAGTTTAAAGGGGTAT
TTGTGAAAAAATAAAATTCGGTGGGCTAAGTGTTACTGCCTCCTTAGTATAGGGTGTTG
TCTATAAAATTTATAAAAAAAAGTACAGCTTGTCAGCTTCGGTCACTGCAATTACCGTT

TGAATACTGTTCGAAAAGCCTTTTCAGCCTTGCATTAGAATTTATTTCGTATAAAGATAT
TCCTAATACAAGGCTATGATGGTTGTTCGAACGGGCCTTGCAGTGAATGATTCCCATCA
CCTTAATTTAGTTCCAAGGGACAATATAACTACAAAATCTCAACTGCCCATGCTCATTAA
TTTTATACTAAAATAAATGGGCGGCTGTGATTATATGATCGGACCAATTCATGGGAAGGG
GACTCGGCTCTGTAGTGATAGGGCTCACAAGTCACAAAGTTTATTTTTTGGTAAATAAT
AATACATTAGAATTTGTTTGTTTTTTTCAGCAGAAAATTGAATTATAAAAAAAATGGCTT
TGGGCCTTTGGGCCGGTATATAACTAACAATTAATTAATGTTTTGTTGCACCAACCAGAT
TCTTGAATGCTGGAATCTTTGGGAACGGAACGGAATCGTGCCACACATTGCCTCACTC
GGCGACCAGAGCGGCCATGCTGGTGAGGATCAACACCCTCCTCCAAGGATACTCTGGC
ATCCGATTTGAGATCTTAGAAGCCATCACTAAATTCCTCAATAATAACATCACCCCATGC
TTGCCCCTGAGAGGCACCATCACCGCCTCCGGCGATCTCGTCCCTCTCTCCTACATCGC
CGGCCTCCTCACCGGCCGCCCCAACTCCAAAGCCGTGGGACCCGATGGCCACATCCTT
AATGCCAGCGAAGCCTTCAACCTTGCCGGGGTCAATGATGGATTCTTTGAGTTGCAGC
CCAAGGAAGGTCTTGCACTTGTTAATGGCACCGCTGTGGGGTCTGGTTTGGCCTCAAT
GGTTCTCTTCGAGACCAACATCCTCGCTGTCTTGTCCGAGGTCTTATCCGCCATCTTCG
CCGAAGTGATGCAAGGCAAACCGGAGTTCACCGACCATCTGACTCACAAACTGAAGC
ACCACCCCGGACAGATTGAGGCTGCAGCTATCATGGAACACATCTTGGACGGAAGCTC
TTACGTCAAGGCAGCTCAGAAGATACACGAGATTGATCCTCTCCAGAAGCCCAAGCAA
GACAGATACGCTCTCCGTACATCTCCCCAGTGGTTGGGCCCCCAAATTGAAGTGATCA
GATCGGCGACCAAGATGATCGAGCGGGAGATCAACTCCGTGAATGACAACCCATTGAT
CGATGTGTCGAGGAACAAGGCCCTCCACGGTGGTAACTTCCAGGGGACCCCCATTGGC
GTTTCCATGGACAACACTCGCTTGGCGATTGCCTCCATTGGGAAGCTCATGTTTGCTCA
GTTCTCCGAGCTTGTTAACGATTTCTACAATAACGGGTTGCCTTCCAATCTATCAGGCG
GTCGCAACCCCAGCTTAGATTATGGGTTCAAGGGTGCCGAGATCGCCATGGCTTCTTAC
TGTTCTGAACTCCAGTTCCTCGCCAATCCGGTCACGAACCACGTCCAGAGTGCAGAGC
AACACAACCAGGACGTCAACTCCTTGGGCTTGATCTCTTCCAGAAAGACAGCCGAAG
CCGTCGACATCCTGAAGCTCATGTCTGCAACATACATGGTTGCACTGTGCCAAGCAATC
GATTTGAGGCACTTGGAGGAGACACTGAAGAGCACAGTCAAGAACACAGTGGGCCA
ACTGGCCAAGAGGGTTCTCACCATGGGCGTCAACGGGGAGCTTCACCCATCCAGGTTC
TGCGAGAAGGATTTGCTCAAGGTCGTCGACCGCGAGTACGTCTTCGCCTACATTGACG
ACCCATGCAGCGCGACGTACCCACTTATGCAAAATCTCAGGCAAGTCCTCGTCCAGCA
TGCACTCCTCAACGGCGAGAATGAAAAGAACTCCAACACCTCAATATTCCAGAAGATC
ACCGCCTTCGAGGAAGAACTGAAGGCCCTTCTGCCCAAGGAAGTCGAGGGTGCCAGA
ATCGCGTACGAGAGTGGGAGCCCAGCAGTCCCCAACCGCATCAAGGAATGCAGATCCT
ACCCGATATACAAATTTGTGAGGGAACAGCTACACACAGGGTTGCTGACCGGCGAGAA
GGTCCGATCACCGGGCGAGGAATTCGACAAGGTGTTCTCAGCAATGTGCGAGGGGAA
AATGATCGACCCACTATTGGATTGTCTCAGGGACTGGAACGGTGCTCCTCTCCCAATCT
GCTAA

>*NnPAL2* coding sequence
ATGGAATTCGCCCACGAGAACTGCAATGGCAACGGCAACGGCAGCATCAAAAGCTTC
TGCGTCCAACAAGACCCACTGAACTGGCAAATGGCCGCCGAGTCTCTCAAGGGAAGC
CACCTGGATGAGGTTAAGCGCATGGTGCAAGAGTACCGGAAGCCGGTGGTCAAGCTA
GGCGGCGAGACCCTTACCATCTCCCAAGTGGCGGCCATCGCCACTCACGACGCCGGTG

TCAAGGTGGAGCTGGCGGAGTCGGCAAGGGCCGGAGTGAAAGCGAGCAGCGACTGG
GTCATGGAGAGCATGAACAAGGGCACGGACAGTTATGGTGTCACGACCGGTTTCGGTG
CAACTTCACACAGAAGAACCAAACAAGGAGGAGCCCTTCAGAAAGAGCTTATTAGAT
TCTTGAATGCTGGAATCTTTGGGAACGGAACGGAATCGTGCCACACATTGCCTCACTC
GGCGACCAGAGCGGCCATGCTGGTGAGGATCAACACCCTCCTCCAAGGATACTCTGGC
ATCCGATTTGAGATCTTAGAAGCCATCACTAAATTCCTCAATAATAACATCACCCCATGC
TTGCCCCTGAGAGGCACCATCACCGCCTCCGGCGATCTCGTCCCTCTCTCCTACATCGC
CGGCCTCCTCACCGGCCGCCCCAACTCCAAAGCCGTGGGACCCGATGGCCACATCCTT
AATGCCAGCGAAGCCTTCAACCTTGCCGGGGTCAATGATGGATTCTTTGAGTTGCAGC
CCAAGGAAGGTCTTGCACTTGTTAATGGCACCGCTGTGGGGTCTGGTTTGGCCTCAAT
GGTTCTCTTCGAGACCAACATCCTCGCTGTCTTGTCCGAGGTCTTATCCGCCATCTTCG
CCGAAGTGATGCAAGGCAAACCGGAGTTCACCGACCATCTGACTCACAAACTGAAGC
ACCACCCCGGACAGATTGAGGCTGCAGCTATCATGGAACACATCTTGGACGGAAGCTC
TTACGTCAAGGCAGCTCAGAAGATACACGAGATTGATCCTCTCCAGAAGCCCAAGCAA
GACAGATACGCTCTCCGTACATCTCCCCAGTGGTTGGGCCCCCAAATTGAAGTGATCA
GATCGGCGACCAAGATGATCGAGCGGGAGATCAACTCCGTGAATGACAACCCATTGAT
CGATGTGTCGAGGAACAAGGCCCTCCACGGTGGTAACTTCCAGGGGACCCCCATTGGC
GTTTCCATGGACAACACTCGCTTGGCGATTGCCTCCATTGGGAAGCTCATGTTTGCTCA
GTTCTCCGAGCTTGTTAACGATTTCTACAATAACGGGTTGCCTTCCAATCTATCAGGCG
GTCGCAACCCCAGCTTAGATTATGGGTTCAAGGGTGCCGAGATCGCCATGGCTTCTTAC
TGTTCTGAACTCCAGTTCCTCGCCAATCCGGTCACGAACCACGTCCAGAGTGCAGAGC
AACACAACCAGGACGTCAACTCCTTGGGCTTGATCTCTTCCAGAAAGACAGCCGAAG
CCGTCGACATCCTGAAGCTCATGTCTGCAACATACATGGTTGCACTGTGCCAAGCAATC
GATTTGAGGCACTTGGAGGAGACACTGAAGAGCACAGTCAAGAACACAGTGGGCCA
ACTGGCCAAGAGGGTTCTCACCATGGGCGTCAACGGGGAGCTTCACCCATCCAGGTTC
TGCGAGAAGGATTTGCTCAAGGTCGTCGACCGCGAGTACGTCTTCGCCTACATTGACG
ACCCATGCAGCGCGACGTACCCACTTATGCAAAATCTCAGGCAAGTCCTCGTCCAGCA
TGCACTCCTCAACGGCGAGAATGAAAAGAACTCCAACACCTCAATATTCCAGAAGATC
ACCGCCTTCGAGGAAGAACTGAAGGCCCTTCTGCCCAAGGAAGTCGAGGGTGCCAGA
ATCGCGTACGAGAGTGGGAGCCCAGCAGTCCCCAACCGCATCAAGGAATGCAGATCCT
ACCCGATATACAAATTTGTGAGGGAACAGCTACACACAGGGTTGCTGACCGGCGAGAA
GGTCCGATCACCGGGCGAGGAATTCGACAAGGTGTTCTCAGCAATGTGCGAGGGGAA
AATGATCGACCCACTATTGGATTGTCTCAGGGACTGGAACGGTGCTCCTCTCCCAATCT
GCTAA

>*NnPAL3* complete sequence
ATGGAATTCGCCCAGGAGAACTGCAATGGCAACGGCTCCCTCAAAAGCTTCTGCATCC
AACAAGACCCATTGAATTGGGAGAAGGCCGCCGAGTCTCTCAAGGGCAGTCACCTCG
ATGAGGTTAAGCGCATGGTCCAGGAATTCCGGAAGCCCGTCGTCCGTCTCGGTGGCCA
GACCCTTACCATCTCTCAAGTGGCCGCCATCGCCACTCACGACGCCGGCGTCAAGGTC
GAGCTCTCCGAGTCCGCCAGAGCCGGAGTCAAAGCAAGCAGCGACTGGGTAATGGAG
AGCATGAACAAGGGCACCGACAGCTACGGTGTCACCACCGGTTTTGGTGCTACTTCCC
ACAGGAGAACCAAACAAGGAGGAGCCCTTCAGAAGGAGCTTATTAGGTAAATTTTTA
AATAAAAATAATAATAAATTCCCACACAAATTTGTCGTATCGCAACCCGGGTGTGCGTC

CGGCTCAGCCAGACACAATTTGTCGGGTTGCTCTGATTGCCCAGAGAACCGGAGAACT
GGTTGTGGTTGGTGGTTGATGGTTGGTGGTGGTGGACGAATGACGATCTATCTGAATCA
GTGAATTGCGGTCTATATTAAAGAATATATAGTCGTATCGCAACCACTGATAATTCACTG
CAATGGACGGCAGTGATTATATCATCAAGCCGAATTATGGGGAAGGAGATTTGGTGATG
GTAGGTAGGTGGACGATCAATAGGAGTAGGTCAAGTTGGAGTTAAGAGTAGGAAAATC
GGGTGGCATTTCAGAATGGGCCCGGGTCGACTCGTTTGGGGGGTGTGCGTGGCTGATA
CCAAAATCCGTGATAAGACACTTGTCACTGATCAGGAGATAAGAACCGTATGTTTTACG
GTTGGACAAGGGCCCACAAAACCTGACCAAAGACAGAGGGTGATCAATAATCAATTC
CCCTGTAACTTCCTACAACCATTTGATTTGGACCGAAAGGAGGAGCTGTGGCCTGTGG
GCCCTCTTTTTAAAATGCTAGGCTGCTAGAGTGCTAGCTAGTAATTTTCAATTTTTCCAT
TTCTAAATTAATCTGTGTAATTTCTGTGCACCAGATTTTTGAACGCTGGAATCTTTGGGA
ACGGAGTGGAATCGTGCCACACGCTGCCACACTCGGCCACGAGAGCGGCCATGCTGG
TGAGGATCAATACACTTCTGCAAGGATACTCCGGCATCCGTTTCGAGATCTTGGAAGCC
ATTACCAAGCTCCTGAACAGCAACATCACTCCATGCTTGCCTCTGAGAGGTACCATCAC
AGCGTCCGGCGATCTCGTCCCGCTGTCCTACATCGCCGGTCTCTTGACCGGCCGACCC
AATTCCGTCGCCGTGGGACCCGACGGACAGAACCTTAATGCCGCTGAAGCCTTCAGCC
TTGCCGGGATCAATGGTGGGTTCTTTGAGCTGCAGCCTAAGGAGGGTCTCGCGCTTGT
TAATGGCACTGCAGTGGGGTCTGGTCTGGCTTCCATGGTTCTCTTCGAGGCCAACGTG
CTCGGTGTCTTATCGGAAGTGTTGTCGGCCATCTTCGCCGAAGTTATGCAAGGGAAGC
CCGAGTTTACCGACCACCTGACTCACAAGTTGAAGCACCACCCGGGTCAAATTGAGGC
TGCAGCTATCATGGAACACATCTTGGACGGAAGCTATTACATTAAGGCAGCTCAGAAG
CTCCATGAGATTGATCCCCTGCAGAAGCCCAAGCAAGACAGATACGCCCTGCGTACGT
CTCCGCAGTGGCTGGGCCCCCAGATTGAAGTGATCAGATCATCGACCAAGTCGATCGA
ACGGGAGATCAACTCTGTGAATGATAACCCATTGATCGATGTCTCCAGGAACAAGGCC
CTCCATGGCGGAAATTTCCAGGGAACTCCAATTGGTGTTTCCATGGACAACACCCGGT
TGGCGATTGCCTCCATCGGAAAGCTCATGTTTGCACAGTTCTCCGAGCTGGTTAACGAT
TTCTACAACAACGGGTTACCATCCAACCTATCAGCAAGTCGAAACCCCAGCTTAGATTA
TGGCTTCAAGGGTGCCGAGATCGCAATGGCAGCCTACTGCTCTGAACTCCAGTTCCTC
GCGAATCCGGTGACCAACCACGTCCAAAGTGCAGAGCAGCACAACCAGGATGTCAAC
TCCTTGGGCTTGATCTCTTCCAGAAAGACTGCCGAGGCAGTGGACATATTGAAGCTCAT
GTCTGCAACATACTTGGTTGCACTGTGCCAGGCAATTGATTTGAGGCACCTGGAGGAG
ATTTTGAAGAGCACAGTCAAGAACACAGTGAGCCAAGTGGCCAAGAGGGTTCTCACC
ATGGGCGTCAACGGAGAGCTCCACCCTTCGAGGTTCTGCGAGAAGGATTTGCTCAAG
GTGGTCGACCGAGAACACGTCTTCGCCTACATCGACGACCCATGCAGCGCCACCTACC
CGCTAATGCAAAAACTCAGGCAAGTCCTCGTCGAGCACGCGCTCCTCAACGGCGAGA
ACGAGAAGAACTCCAACACCTCAATCTTCCAGAAGATCACCGCCTTGGAGGAGGACC
TTAAGACGCTTCTGCCCAAAGAAGTAGAGGCTGCGAGAATCGCCTACGAGAGCGGGA
GCCCGGCAATCCCGAACCGCATCAAGGAATGCAGGTCATACCCATTGTACAAGCTCGT
GAGAGAAGAGCTGCAGACCGGTTTGCTGACCGGCGAGAAGGTCCGATCACCGGGGG
AGGAGTTCGACAAGGTGTTCACTGCAATATGCCAGGGGAAAATAATCGATCCTCTATTG
GATTGTCTCAAGGGTTGGAACGGTGCCCCTCTTCCAATCTGCTAG

>*NnPAL3* coding sequence

ATGGAATTCGCCCAGGAGAACTGCAATGGCAACGGCTCCCTCAAAAGCTTCTGCATCC

AACAAGACCCATTGAATTGGGAGAAGGCCGCCGAGTCTCTCAAGGGCAGTCACCTCG
ATGAGGTTAAGCGCATGGTCCAGGAATTCCGGAAGCCCGTCGTCCGTCTCGGTGGCCA
GACCCTTACCATCTCTCAAGTGGCCGCCATCGCCACTCACGACGCCGGCGTCAAGGTC
GAGCTCTCCGAGTCCGCCAGAGCCGGAGTCAAAGCAAGCAGCGACTGGGTAATGGAG
AGCATGAACAAGGGCACCGACAGCTACGGTGTCACCACCGGTTTTGGTGCTACTTCCC
ACAGGAGAACCAAACAAGGAGGAGCCCTTCAGAAGGAGCTTATTAGATTTTTGAACG
CTGGAATCTTTGGGAACGGAGTGGAATCGTGCCACACGCTGCCACACTCGGCCACGA
GAGCGGCCATGCTGGTGAGGATCAATACACTTCTGCAAGGATACTCCGGCATCCGTTTC
GAGATCTTGGAAGCCATTACCAAGCTCCTGAACAGCAACATCACTCCATGCTTGCCTCT
GAGAGGTACCATCACAGCGTCCGGCGATCTCGTCCCGCTGTCCTACATCGCCGGTCTCT
TGACCGGCCGACCCAATTCCGTCGCCGTGGGACCCGACGGACAGAACCTTAATGCCGC
TGAAGCCTTCAGCCTTGCCGGGATCAATGGTGGGTTCTTTGAGCTGCAGCCTAAGGAG
GGTCTCGCGCTTGTTAATGGCACTGCAGTGGGGTCTGGTCTGGCTTCCATGGTTCTCTT
CGAGGCCAACGTGCTCGGTGTCTTATCGGAAGTGTTGTCGGCCATCTTCGCCGAAGTTA
TGCAAGGGAAGCCCGAGTTTACCGACCACCTGACTCACAAGTTGAAGCACCACCCGG
GTCAAATTGAGGCTGCAGCTATCATGGAACACATCTTGGACGGAAGCTATTACATTAAG
GCAGCTCAGAAGCTCCATGAGATTGATCCCCTGCAGAAGCCCAAGCAAGACAGATACG
CCCTGCGTACGTCTCCGCAGTGGCTGGGCCCCCAGATTGAAGTGATCAGATCATCGAC
CAAGTCGATCGAACGGGAGATCAACTCTGTGAATGATAACCCATTGATCGATGTCTCCA
GGAACAAGGCCCTCCATGGCGGAAATTTCCAGGGAACTCCAATTGGTGTTTCCATGGA
CAACACCCGGTTGGCGATTGCCTCCATCGGAAAGCTCATGTTTGCACAGTTCTCCGAG
CTGGTTAACGATTTCTACAACAACGGGTTACCATCCAACCTATCAGCAAGTCGAAACCC
CAGCTTAGATTATGGCTTCAAGGGTGCCGAGATCGCAATGGCAGCCTACTGCTCTGAAC
TCCAGTTCCTCGCGAATCCGGTGACCAACCACGTCCAAAGTGCAGAGCAGCACAACC
AGGATGTCAACTCCTTGGGCTTGATCTCTTCCAGAAAGACTGCCGAGGCAGTGGACAT
ATTGAAGCTCATGTCTGCAACATACTTGGTTGCACTGTGCCAGGCAATTGATTTGAGGC
ACCTGGAGGAGATTTTGAAGAGCACAGTCAAGAACACAGTGAGCCAAGTGGCCAAG
AGGGTTCTCACCATGGGCGTCAACGGAGAGCTCCACCCTTCGAGGTTCTGCGAGAAG
GATTTGCTCAAGGTGGTCGACCGAGAACACGTCTTCGCCTACATCGACGACCCATGCA
GCGCCACCTACCCGCTAATGCAAAAACTCAGGCAAGTCCTCGTCGAGCACGCGCTCCT
CAACGGCGAGAACGAGAAGAACTCCAACACCTCAATCTTCCAGAAGATCACCGCCTT
GGAGGAGGACCTTAAGACGCTTCTGCCCAAAGAAGTAGAGGCTGCGAGAATCGCCTA
CGAGAGCGGGAGCCCGGCAATCCCGAACCGCATCAAGGAATGCAGGTCATACCCATTG
TACAAGCTCGTGAGAGAAGAGCTGCAGACCGGTTTGCTGACCGGCGAGAAGGTCCGA
TCACCGGGGGAGGAGTTCGACAAGGTGTTCACTGCAATATGCCAGGGGAAAATAATCG
ATCCTCTATTGGATTGTCTCAAGGGTTGGAACGGTGCCCCTCTTCCAATCTGCTAG
