# Supplementary figures and images for: Molecular evolution and functional characterisation of an ancient phenylalanine ammonia-lyase gene (NnPAL1) from Nelumbo nucifera: novel insight into the evolution of the PAL family in angiosperms
Source: BMC Evol Biol. 2014 May 9;14:100. doi: 10.1186/1471-2148-14-100 (PMC4102242; doi:10.1186/1471-2148-14-100)

Figure S2. Sequence alignment between NnPAL1 and other typical PALs in seed plants.

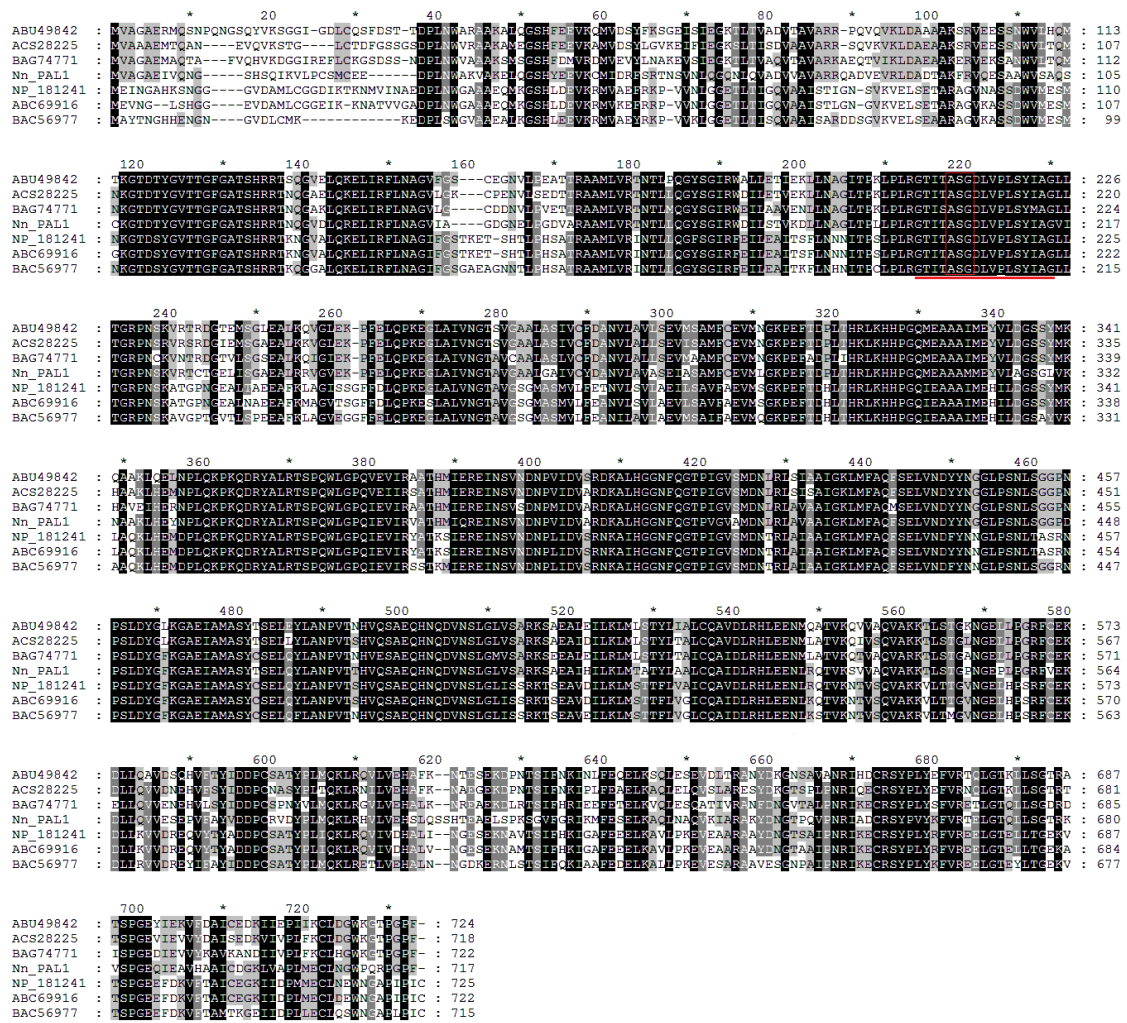

Supplement: Additional file 2: Figure S2 — Sequences alignment of NnPAL1 and other typical PALs from seed plants. The phenylalanine and histidine ammonia-lyase signature (GTITASGDLVPLSYIA) is underlined with red lines, and the conserved Ala-Ser-Gly triad is framed in a red box. [file 1471-2148-14-100-S2.pdf]
