# Supplementary material for: Molecular evolution and functional characterisation of an ancient phenylalanine ammonia-lyase gene (NnPAL1) from Nelumbo nucifera: novel insight into the evolution of the PAL family in angiosperms
Source: BMC Evol Biol. 2014 May 9;14:100. doi: 10.1186/1471-2148-14-100 (PMC4102242; doi:10.1186/1471-2148-14-100)
Supplement: Additional file 3: Figure S3 — The nucleotide sequence and deduced amino acid sequence of NnPAL1. The start codon (ATG) and stop codon (TAA) are underlined. The typical phenylalanine and histidine ammonia-lyase signature is boxed. Nine strictly conserved residues, Y112,L140,S204,N260,Q348,Y351,R354,F400,Q488, are marked in red italics. [file 1471-2148-14-100-S3.pdf]

Figure S3. The nucleotide sequence and the deduced amino acid sequence of *NnPAL1*.

gaaaacccagttcaatccctcaatttccggtcccccgctctctctctctctgtctcgaacctcttgcacgcgtcacc

ATGGTTGCAGGGGCCGAGATAGTGCAGAATGGCTCGCACTCGCAGATCAAAGTCCTTCCATGCAGCATGTGCGAG

M V A G A E I V Q N G S H S Q I K V L P C S M C E

GAGGATCCTTTGAACTGGGCCAAGGTGGCCAAGGAGCTCCAAGGTTCGCACTATGAAGAAGTGAAGTGCATGATC

E D P L N W A K V A K E L Q G S H Y E E V K C M I

GATCGATTCAGTCGAACCAACTCAGTCAACTTGCAAGGTCAGAACCTCCAAGTCGCAGACGTCGTCGCCGTCGCT

D R F S R T N S V N L Q G Q N L Q V A D V V A V A

CGTCGCCAAGCCGACGTCGAAGTCCGACTCGACGCCGACACTGCCAAGTTCCGGGTGCAAGAGAGCGCTGCCTGG

R R Q A D V E V R L D A D T A K F R V Q E S A A W

GTCTCGGCTCAGTCCTGCAAAGGTACCGACACTTATGGCGTCACAACCGGTTTCGGCGCCACCTCGCACCGCCGG

V S A Q S C K G T D T *Y* G V T T G F G A T S H R R

ACGAACCAAGGGGTTGATCTTCAGCGGGAGCTTATCAGATTCTTAAACGCCGGCGTCATTGCCGGAGACGGAAAT

T N Q G V D L Q R E L I R F *L* N A G V I A G D G N

GAGCTCCCCGGCGATGTTGCCCGAGCAGCCATGCTTGTACGTACCAACACTCTTCTTCAAGGCTACTCGGGCATC

E L P G D V A R A A M L V R T N T L L Q G Y S G I

AGATGGGACATACTTAGTACCGTCAAGGACCTCCTCAACGCTGGCCTGACACCGTTACTCCCCCTCCGCGGCACA

R W D I L S T V K D L L N A G L T P L L P L R G T

ATCACAGCCTCCGGCGACCTCGTCCCGTTGTCCTACATTGCAGGAGTAATCACCGGGCGTCCCAACTCCAAGGTC

I T A *S* G D L V P L S Y I A G V I T G R P N S K V

CGTACATGCACAGGCGAGCTGATCTCCGGAGCGGAAGCTCTCCGGCGCGTGGGAGTGGAAAAGCCATTCGAGTTA

R T C T G E L I S G A E A L R R V G V E K P F E L

CAACCAAAGGAGGGGTTAGCCATAGTCAACGGAACCGCAGTGGGAGCAGCGCTGGGGGCTATCGTGTGTTACGAC

Q P K E G L A I V *N* G T A V G A A L G A I V C Y D

GCTAACGTCCTGGCGGTGGCGTCTGAGATCGCATCGGCGATGTTCTGCGAAGTGATGCTGGGGAAGCCGGAGTTC

A N V L A V A S E I A S A M F C E V M L G K P E F

ACGGATCCGTTGACTCACCGGCTGAAGCACCATCCGGGTCAGATGGAGGCGGCGGCAATGATGGAGTACGTTCTC

T D P L T H R L K H H P G Q M E A A A M M E Y V L

GCCGGAAGCGGCTTAGTTAAGAATGCGGCAAAGCTTCACGAATACAATCCCTTGCAGAAGCCGAAACAAGACCGA

A G S G L V K N A A K L H E Y N P L Q K P K *Q* D R

TATGCTCTTCGTACTTCTCCCCAGTGGCTGGGCCCACAGATCGAGGTGATTAGAGTGGCCACCCACATGATTCAA

Y A L *R* T S P Q W L G P Q I E V I R V A T H M I Q

CGTGAAATAAACTCAGTGAATGACAACCCGGTTATTGATGTGGCCCGAGACAAAGCCCTCCACGGTGGCAACTTC

R E I N S V N D N P V I D V A R D K A L H G G N *F*

CAGGGGACCCCAGTCGGTGTAGCGATGGACAATCTACGTCTAGCCGTGGCGGCGATCGGAAAGCTGATGTTCGCG

Q G T P V G V A M D N L R L A V A A I G K L M F A

CAATTCTCTGAGCTAGTGAACGACTACTACAACGGAGGCCTGCCTTCCAACCTCAGCGGCGGACCCGACCCCAGC

Q F S E L V N D Y Y N G G L P S N L S G G P D P S

CTGGACTACGGATTCAAGGGTGCTGAGATTGCCATGGCATCATACACGTCAGAGCTTCAGTATTTGGCAAACCCA

L D Y G F K G A E I A M A S Y T S E L Q Y L A N P

GTCACAACCCATGTACAGAGTGCCGAGCAACACAACCAGGATGTTAACTCTCTCGGCTTGGTATCCGCCCGGAAG

V T T H V Q S A E Q H N *Q* D V N S L G L V S A R K

TCGGCGGAGGCTATCCACATCCTCAAGTTGATGACTGCAACCTACCTGGCCGCGCTCTGCCAAGCCATTGATCTC

S A E A I H I L K L M T A T Y L A A L C Q A I D L

CGCCATCTTGAGGAGAATCTCCGCCAGACCGTCAAATCCGTTGTTGCACAGGTAGCAAAGAAGACCCTAAGCACA

R H L E E N L R Q T V K S V V A Q V A K K T L S T

GGACCCAACGGTGAGCCGCTCCCTGGCCGATTTGTTGAGAAAGACCTGCTCCAAGTAGTGGAGAGCGAACCAGTA

G P N G E P L P G R F V E K D L L Q V V E S E P V

TTTGCCTATGTGGACGACCCTTGCCGTGTCGACTACCCTCTCATGCAGAAGCTCCGGCATGTCCTCGTTGAACAC

F A Y V D D P C R V D Y P L M Q K L R H V L V E H

TCACTACAGAGCTCGCATACAGAGGCGGAGCTGTCGCCCAAATCTGGTGTCTTCGGACGGATAAAGATGTTCGAA

S L Q S S H T E A E L S P K S G V F G R I K M F E

TCCGAGCTCAAAGCGCAGCTCAATGCCCAAGTTAAAATTGCGCGTGCCAAATATGATAACGGAACCCCACAGGTT

S E L K A Q L N A Q V K I A R A K Y D N G T P Q V

CCCAACAGGATCGCCGATTGCCGGTCGTATCCGGTCTACAAGTTTGTCCGTACAGAGCTTGGTACTCAGCTGCTT

P N R I A D C R S Y P V Y K F V R T E L G T Q L L

AGTGGCACCAGAAAAGTGTCTCCTGGGGAACAAATCGAGGCCGTCCATGCGGCTATCTGCGATGGCAAACTGGTT

S G T R K V S P G E Q I E A V H A A I C D G K L V

GCTCCATTGATGGAGTGCTTGAATGGATGGCCCCAGAGGCCTGGGCCATTTTAAtttgctttactttggtgtt

A P L M E C L N G W P Q R P G P F *

gccttcttgtgttttactttttctttctcctcctctacggtttgtaggaagaggcgattccaaataaattataga

tgttgcaaaaaaaaaaaa
