# Supplementary material for: Molecular evolution and functional characterisation of an ancient phenylalanine ammonia-lyase gene (NnPAL1) from Nelumbo nucifera: novel insight into the evolution of the PAL family in angiosperms
Source: BMC Evol Biol. 2014 May 9;14:100. doi: 10.1186/1471-2148-14-100 (PMC4102242; doi:10.1186/1471-2148-14-100)
Supplement: Additional file 4: Figure S4 — Deduced amino acid sequences from the PALs of Pinus taeda. [file 1471-2148-14-100-S4.pdf]

Figure S4. Deduced amino acids sequences from PAL transcripts of *Pinus taeda*

>Pteda1143311
MVAAAEITQANEVQVKSTGLCTDFGSSGSDPLNWVRAAKAMEGSHFEEVKAMVDSYFGAK
EISIEGKSLTISDVAAVARRSQVKVKLDAAAAKSRVEESSNWVLTQMTKGTDTYGVTTGF
GATSHRRTNQGAELQKELIRFLNAGVLGKCPENVLSEDTTRAAMLVRTNTLLQGYSGVRW
DILETVEKLLNAWLTPKLPLRGTITASGDLVPLSYIAGLLTGRPNSRVRSRDGIEMSGAE
ALKKVGLEKPFELQPKEGLAIVNGTSVGAALASIVCFDANVLALLSEVISAMFCEVMNGK
PEFTDPLTHKLKHHPGQMEAAAIMEYVLDGSSYMKHAAKLHEMNPLQKPKQDRYGLRTSP
QWLGPQVEIIRSATHMIEREINSVNDNPVIDVARDKALHGGNFQGTPIGVSMDNLRLSIS
AIGKLMFAQFSELVNDYYNGGLPSNLSGGPNPSLDYGLKGAEIAMASYTSELLYLANPVT
SHVQSAEQHNQDVNSLGLVSARKSAEAIDILKLMLSTYLTALCQAVDLRHLEENMLATVK
QIVSQVAKKTLSTGLNGELLPGRFCEKDLLQVVDNEHVFSYIDDPCNASYPLTQKLRNIL
VEHAFKNAEGEKDPNTSIFNKIPVFEAELKAQLEPQVSLARESYDKGTSPLPDRIQECRS
YPLYEFVRNQLGTKLLSGTRTISPGEVIEVVYDAISEDKVIVPLFKCLDGWKGTLAHSEI
NNLPRSPLYNDCYDLSPRMLLLMLLFSDPEFDWS

>Pteda17307
MVAGADLGAVQANGNQNGNGFHHVHSVDLCIQNGPDPLNWGQAAKALQGSHFEEVKLMVE
SYFGSEEVSIEGKSLTIADVTAVARRPEAKVKLDAVSAKARVDESSNWVLQNMLKGTDTY
GVTTGFGATSHRRTNQGAELQKELIRFLNSGVLTDGNVLPQETTRAAMLVRTNTLMQGYS
GIRWEILETIQKLLNAGITPKLPLKGTITASGDLVPLSYIAGFITGRPNSKARCRDGKEL
GALEALQQIGVEKPFELQPKEGLAIVNGTSVGAALASIVCFDANIICIAAEVLSAMFCEV
MLGKPEFTDPLTHRLKHHPAQMEAAAIMEYVLDGSSYMKNAAKKHEMNPLQKPKQDRYAL
RTSPQWLGPQIEVIRSATHMIQREINSVNDNPVIDVARDKALHGGNFQGTPIGVSMDNLR
LALAAIGKLMFAQFSELVNDYYNGGLPSNLSGGPNPSLDYGFKGAEIAMASYTSELQYLA
SPVTTHVQSAEQHNQDVNSLGLISARKSAEAIDILNLMVSTYLLALCQAADLRHLEENML
STVKSVVSHVAKKMLSTHNGELLTAGRFCEKDLLQAVENLHVFAYVDDPCNENYPLMQQL
RQVLVAHALTETAQIQTQTQSSIFNKIPAFEKELKDQMEAEIGRARQDYYERGVAGSIPN
RIQECRSFPLYDFARSQLGTQLLSGDRVTSPGEYIEKVYTGIREGKIISPLFKCLDGWSG
TPGPFHS

>Pteda28316
MAPQEFTGEVKFCAGNGGTASLNDPLNWAAAAESMKGSHFEEVKRMWEEFRSPVVRLQGS
GLTIAQVAAVARRMGSVRVELETGAKARVDESSNWVMDSMANGTDSYGVTTGFGATSHRR
TRQGEALQKELIRFLNAGIFGGCGDSNSLPRDTTRAAMLVRANTLLQGYSGIRWGILEAM
SGLLNAGITPRLPLRGTITASGDLVPLSYIAGLLTGRSNARAVTADGKELGAAEALAAAG
VENGPFELRPKEGLALVNGTAVGSALAATVLFDANVVVLLSEVLSALFCEVMQGNPEFTD
HLTHRLKHHPGQIEAAAIMEHLLDGSSYMKAAAAKHQEADALSKPKQDRYALRTAPQWLG
PQVEVIRASTHMVQREINSVNDNPLIDAARNKALHGGNFQGTPVGVAMDNARLALAAVGK
LMFAQMSELVNDFYNNGLPSNLSGGPDPSLDYGFKGAEIAMAAYTSELQFLANPVTTHVQ
SAEQHNQDVNSLGLISARMTAQAVEILKLMTSTYLVALCQAIDLRHLEENLLAAVRQSVG
QACKKTLVVGPGGELLPSRFCEKDLLKAIDREPVFSYIDNPCSATSVLTTKLRQVLFEHA
IEKTANNDASILTRIPAFEEELKARIVAEVQETRGAFEKGAALVPNRIKDCRSYPLYEFV

RVELGASLLVGTNSRSPGEDFDKVFVAINEGKAVEPLFKCLERWNGAPIPI

>Pteda34319
MNLCAGNDPLNWASVAESMKGSHFEEVKRMVEEFRAPVVRLQGSGLTIAQVAAVARRLGS
VRVELDTGARARVEESSNWVMDSIANGKAIYGVTTGFGASSHRRTSHGEALQKEMARFLN
AGIFGGCGDSNTLPRDATRATMLVRTNTLLQGYSGIRWGILEAMTGLLNAGITPRLPLRG
SITASGDLVPLSYIAGLLIGRPNARAVMADGTEVGAAEALAAAGVGNGPFVLRPKEGVAL
VNATAVGSALAATVLFDANVVVLLSEVLSALFCEVMQGDPGFTNHLIHRLKDHPGQIEAA
AIMEHLLDGSSYMKAAAAKNKEADPLSKPKKDRYALYTSPQWLGPQVEVIRASTHMVQRE
INSVNDNPIIDAAGNKALNGGNFQGTPVGVAMDNVRLALAAVGKLIFAQMSELVNDFYNK
GLPSNLSGGPDPSLDYGFKGAEVAMASYTSELQFLANPVTTHVQSAEQHNQDVNSLGLIS
ARMTAQAVEILKLMTSTYLVALCQAIDLRHLEENLHAAVRQAVGEACKKTLVVGPRGELL
LLKAVDREPVFSYIDNPCSATSVLTTVLRQVLFEHALEKTTDNDGSILTRVPAFEEELKA
RIVADVHETRAACEKGTALVPNRIKDCRSYPLYEFVRAELGTSLLVGTDSRSPGEDFDKV
FVAINEGKAVAPLFKCLEGWNGAPIPI

>Pteda9006
MSSSVGEESSRMEIMSSSVGEESSRMENINGSMRSMELCRPPTLPLPGPLDGKNPDHVTF
PTHWKKAAEAMQCSHYEEVRKMIKQFNTTRKVVLRGTTLTVAEVTAVTRRVEVRVELDEA
SAKERVERSYQWVAKNVARGTDTYGVTTGFGATSHRRTDKAADLQKELIRFLNAGVVGKE
RLCLPAEYTKAAMLVRTNTLMQGYSGIRWEILDALRKLMDCNITPKLPLRGTITASGDLV
PLSYIAGLLTARPNSKALSPDGHLLDAMEALRKAGILEPFKLQPKEGLALVNGTAVGSAV
AASVCFDANVLGVLAEILSALFCEVMQGKPEFVDPLTHQLKHHPGQIEAAAVMEFLLDGS
DYVKEAARLHEKDPLSKPKQDRYALRTSPQWLGPPIEVIRAATHSIEREINSVNDNPLID
VSRDMALHGGNFQGTPIGVSMDNMRISLAAVGKLLFAQFSELVCDYYNNGLPSNLSGGPN
PSLDYGFKGAEIAMAAYTSELQYLANPVTTHVQSAEQHNQDVNSLGLISARKTAEAVEIL
KLMFATYLVALCQAIDLRHLEENMRSVVKHVVLQAARKTLCTAEDGSLHDTGFCEKELLQ
VIDHQPVFSYIDDPTNPSYALMLQLREVLVDEALKSSCPDGNAESDHNLQPAESAGAAGI
LPNWVFSRIPIFQEELKARLEEEVPKARERFDNGDFPIANRINKCRTYPIYRFVRSELGT
DLLTGPKWRSPGEDIEKVFEGICQGKIGDVILKCLDAWX
