# Supplementary material for: Molecular evolution and functional characterisation of an ancient phenylalanine ammonia-lyase gene (NnPAL1) from Nelumbo nucifera: novel insight into the evolution of the PAL family in angiosperms
Source: BMC Evol Biol. 2014 May 9;14:100. doi: 10.1186/1471-2148-14-100 (PMC4102242; doi:10.1186/1471-2148-14-100)

Figure S5. Phylogenetic trees of the phenylalanine ammonia lyase gene family constructed by BI method (a) and NJ method (b). Posterior probability and bootstrap values demonstrated on each branch of BI tree and NJ tree, respectively.

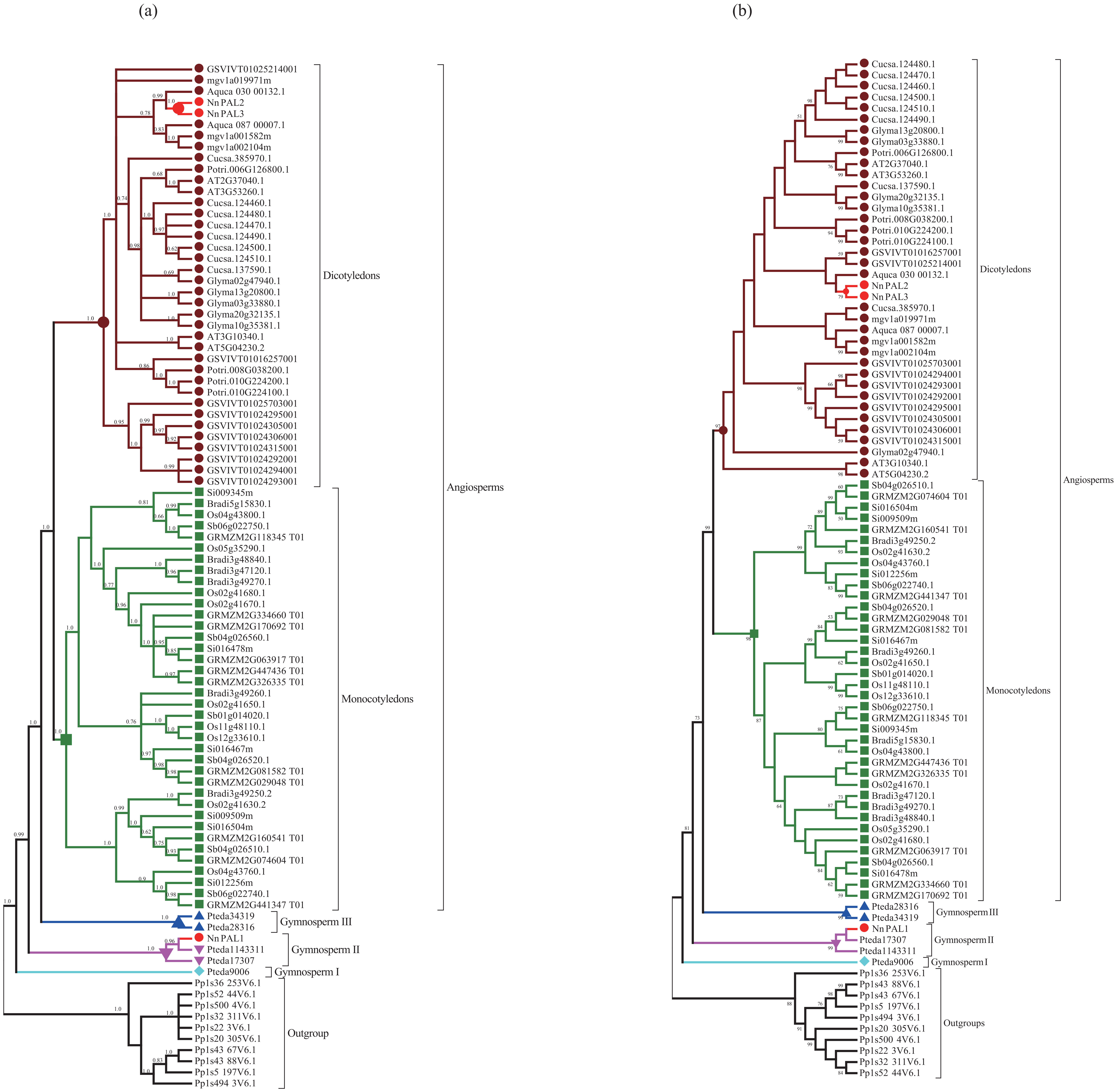

Supplement: Additional file 5: Figure S5 — Phylogenetic trees of the phenylalanine ammonia lyase gene family constructed using the BI method (a) and NJ method (b). The posterior probability and bootstrap values (>50%) for the two trees are shown on each branch, respectively. [file 1471-2148-14-100-S5.pdf]
