# Supplementary material for: Molecular evolution and functional characterisation of an ancient phenylalanine ammonia-lyase gene (NnPAL1) from Nelumbo nucifera: novel insight into the evolution of the PAL family in angiosperms
Source: BMC Evol Biol. 2014 May 9;14:100. doi: 10.1186/1471-2148-14-100 (PMC4102242; doi:10.1186/1471-2148-14-100)
Supplement: Additional file 6 — Identification of the upstream sequence (31,942 bps) and downstream sequence (26,288 bps) of NnPAL1. [file 1471-2148-14-100-S6.pdf]

Additional file 6. Identification of upstream sequence (31,942 bps) and downstream sequence (26,288 bps) of *NnPAL1* marked with shadow boxes. The coding sequence of *NnPAL1* marked at the posotion of start codon and stop codon with red box and yellow box, respectively.

CTGCACCAGGGAGCTCCACCATCCTCTGAGTTGCAGGCTGCCTAGGAGCAAGTCAAATGACGAGTCCACCA
GGTAGTTCGCCACCAGGCAATTCGCCACCAGGGAGCTCGCCACCAGGGAGTTCGCCACCAGGCAGTTCGC
CACCAGGGAGTTCGCCACCAGGGAGCTCGCCACCAGCCAAATCGCCACCAGGCAGTCCACCACCAGGCAG
CTCGCCACCTGGGAGCTCACCACCAAGCAGTTCGTCACCAGGGAGCTCACCACCGGGCAGTTCTCCACCA
GGGAGTTTGCCACCAAGTAGTTTGTCACCAAGTAGCTCTCCACCAGGGAGCTCGCCACCAAGCAGTTCGTC
ACCAAGTAGCTCTCCACCGGGGATCTCGCCACCAGGCAGTTCTCCACCAGGGAGTTCGCCACCAAGTTGTT
CATCACCAAGGCACGTCACCACCGGGTCAAATAACGAGGTACTTAGCCAAAAAAAATTTATACATCACAAAA
AATGGTACGTGCCCAGAAACGGTTACATCACTTTCCATCATCTCCTTCAAGTGAAAAACCAAATATCCAGTGT
ATGTAGTATTGTAATAGTATGTAGTATCATGCCCTCCTTTGTCTTGTAATTGTAAAGTTGTAATATATATTCTGCAT
CATGATTCATAACTGCATCTAGCCAACCCTGGTAGGTTGGTACAGTGGTAGTGTCGTAGTGTGGTACCTGCA
GGTCCTGCACTAAAACAACTAAACAAGACTCCAATGGAACTATAGCTTACCAGAGGGGTTAGTAGATCACG
AATCACGTGCCATGGCAGGATTGGCGACGAGAAATGTTCTCCGAGACTCCGACTTCTCCCAATATTTTTAAA
ATTTATTTTTCATTATTTCAAAAATTCATTTAAATTGTGGGTTATTTCGTTTTCCTTCTCATGTGTGAGAAAATAT
CTGGTCAAATGGCCAGTACCACCCTTGCTACAATAGCTAAAGTCTTTTCTACATTTTTTGCAAACTGCTCTTTT
CCCCTTCCCCTCAACATCCTCTATTTTAAATATTCTCCAAATCAAGTTACTAAACTTGGGGTTTTGGGAAGAGT
TGACCATTATTTTCAAATTCTAAAAAAATAATAAAATTAATTTAATCGGACTTACCCCAGAATCCCGGATTGCC
AGTTCAAAATCCCAACTTGATTTCAATGGTTAAAGCCTTTCAACGTGACAACATCCAAGGCTCAAAGCCTCC
CAAATTGCTAACTTGGAGAATCAGACAATCAACCAATCAAACTATAAGACTATCAGAGTTCACTCATATGGTA
GAGAATGTATGAGAGACTGAAAGAGACCGAGAGTTTGAGAGACTGAGAGAGGAAAGACTGAAGAGATTA
AGAGAATAACTTAATAGGAGAATTGTTGAATGTTGATTGTTAAATTGGTGTGATGAAAATGGTGGAGGATAA
AGCTATTTATAGGAATTGAACATAATTCAATTACCCAAAAAACTATTTGTTATTTAGTTGTTGGGAGGGGTATT
TTGATCATTGTAAAATTACTTCTAATTTTACCCAAAATGCCTCATAAACTAGTTGTTATATAACTATTGGGGGGT
ATTTTGTAAAATACCCCCAAAAAGCTGAAACTAGCCGTTGAGCCCCTTTGGGAAGGGGGGAACCACCCAA
GTCCCAAGTCCCAAGGCTCCCAATGACTATATTCCCGAACGGGCCGAGCATGGCCCGTTACGGGCCTGGCA
GGCTGTGCTTTGTGTTGTGCCACGAGCTGGGGCATGATGGCACGTGAGCCACGAGCGACCCGGCCCGATT
TACACATCTAGTGAAAGATGCATGTGAGCCTAAGGCCCAATGGCCATACCACATAGAGAGGTTTGATCCTAC
ACCTCTATTTAGGAGTGAGGTTGAATGATCATCTCAAGTTGTTGCTATAGCTTCTTCGCCCTTTTTTCTATTCTT
TTTCTTTGGATAACAAGAGGGATTCAGCCCCTATATTTTCTGAGTTTTCTTTTCTTTTTCTTCTCGAGAATATGT
TGATTTTCTACATATAAATTTTTATATGAAATACATACTTATTTTATGAAAACTATTGAAAATTACTAGTGAAAGA
CAATTGCTAATTGCTATGAATCATAGGAAATTACCTAAGACTTTAGGGTTTATTTAAAAAATTAAATTGCCTAG
GCAATAAGGTGCTACCTGTCTTGTTGGCATGATGATTTCCCAAAAACCCATACCACGTAGGATCCACACTAGA
ATTTGATGAATCAGTGATTTTTTTCATGTTTGGTTGGTCGGATTATGCCGATATTTGGGAAATCATCCAAAAAT
CAATTATTAAAATCAGTCGTTTTCAAGGATCACCTTTTTTTGGTGATTTCCTGATTTTTAACTGATTCCGATATT
TTGATGGCATGGAATTGAATAATAACTCTAATACAAAAATAATTGGCCCTTGAAAATCAGTACCAAACAGACT
CAAAGCCAATTTCTCAAAAATCAGCTTACATGCATAAATGATATCAAACAACAATTTTTTACGATTTTTCAAAA
ATCACTGATTCCAGAAATCACCGATTTTTGAATAATCAGCAAGGAGAAATCATCATCGATGCCAAACATCACC
AGAATTAGCAGTTAAAAGAATGAGTTCCACTGATGCTGGAAATACATGATGGCAGAAAAACTTATACCAATC
AACCGGCTTCATAAATGTGGTAACCAATAAGGTGAGTTTTTAACAAAAATTGATGGGGAGTTTTCATTGGTC
AAATCCCTTTGGGTATCACGTCTTTCCAGCATCATAGGTTTTTCCTTAAAAATTCAAATCATGTAATTTAACATA
ACATAAAATGAAAGAAACAACAGTTACCAAAAAAAATGAAAGAAACAATTATAAAACTCCAATAGTTGGGA
GCTTATGGAATCATCAAACTGAAGTGAGAGAATCTTGTTTATATTCTTGGAGCTAATAAAAGATCCTGAAACA

AACAATTTGGAGAAAATGACTCGCATCATTGTCTTCATTTGGCAGAAGATAATATAACTAAGTGGGCAACAA
ACACTACCAAGCTTTAACATGGGCTGATGGGGTAAATTTTGAAACCAGAAAGCTTTCCATAACCACCAAATA
GATCAGATTTTAGGCACCTATGTTAACATATCATCCTCTCCTGTCAATATCTGCAAATTGATCTACTGATCAAAA
AAAAATCTGCAAATTGATCTGGCAGATACCCAGGGAAAGACAGGGTCCATAAAGATGTCTTACAATGTAGA
AATTCAATGCCTCTGTACCCTTGTTATGGTTCATTATTTTTTGAAGTTTTCACTATTAGTTTGAACAGGCAGAA
ACTGCCACAGATTATGCTATATTTACCTATAGAAGCACAAGACAAATTTTTCCTTTCTCTGTAATTATTTCCTAT
GAAGCTGCAATGGAGATTCGACAGCCTTGCTTTAACCTACAGAACCAAAAAGTAAGTACCTACTGAAAATTT
TAAAACAAGCAGAACTTTACCAGACAAAATCTCAACTCTGCCTTCTGGAGAAATATAACTTCTCTCCCCACTT
ATTATCGAATCCTCAGCAACAAAAAATCCACGATGTTCAATTATCTGAGTCTGTCCCAAACAGCTTCATCCTCG
ACAATGGAGTCTGCAAGAACCCCTCCACCTCGAATTTCTTGGGACAAAGTTCGTGGTATTTCTTAAACTGCT
CTCTCGCCTCCTTGTTCCGATCAAGCAAGCTATAAATCACCCCTTGGCAAAAGTAAGGCCTGAAATCCTTGG
GGTCCTCCTTGGCCAATTCCTGATAACTCCTCAATGCCTCATCCACATTCTTCTGAAGATACTGAATCTGTGCC
ATTATCAACCTTACGTCGCGAGCTTCTTTCACCTTGTTTTCCTCCTCTGCAAGTTTCAGGGCTTCTTGAAGCC
GGTTAAGCACTGCTTCACCTTCTCCACAGCGGTCCATAAGCAAGGCATTCTCGAATAGAGCTTCGAAAGAG
AGAGGATTCACAGCCAAAATAGCTTCAAAAACCTGCCGAGCTTCATCAACTTTCCCCATTTCATTCAATAATC
TCGCCACGAGAAATTTCCATTCAACCTCCGATGGTTGAGCAGAAACCAAGCGTTCCAATATCTTCAAAGCTT
CCTCATCCTCTCCATCTTCCAGCTTCCGTTGCAAGAGAGATTTCAAGGCGTCAATGGCGTCAGAATGCGATTC
TAGAAACTGAGACAGCGGAGATTCCTGGGATTTCTCCTGCTCCTGCTCTTGTCCCAGCTCTTCTTCCAACAC
AGGTTTTTCCTCCGTGAACGTCGCCGGGGGTTCAGCCCTGGCCGGAAACTGAGAGAACTTTCCGACCATCA
ATGCGGCCACTGCAGTTGCTAGAATCGCCGTTGAGGCAGTGCTCTTAATTCTTCGGATTATGAGGTTTTTGTC
ACAGTTAGGACTGGAACTCGAAGAAACCCTAACGCTTATAACCCTAGCCCTGGTTTGGGAAATTGAAGGTG
GTTGGGTGGAGAAACGGAGATGGGAAGGTGATGATGAAGTGAAGGAGATAGGGAGAGATGTTCTGCCAT
GGATGAGAGCTCCTCGGATTAAGGAAGTATCCATGGCTAGGGTTAGGGTGTAGGGTTTTATCTCTTGATGGC
AAAGAAAGACGAAGGGGTTGAAAACAATGAGCAGGGCTTTTGCTATTGCTGTTTGTTATGCAGAGAATGGT
ACGAGCGGGAGAGTATCAAAATTACATTGATGTCCGGAACTTTTCTTTATTTCCTTCTCACATGTGGAACTGA
CGAATTGTAGGTGTGGGGCCTAGAGGGATCGAGACGGTTTTGCTTTGCTATCGGGTGATCTAGGCGTCTATC
AACTCTCAGTGCATCTGCCATCTGATTTTAATGGGAGTGTTTAATGAGTTGAGAATTTCCGCCAACAAAACAC
TTTCCAACCCTTTACCAATTTCATCTAATACTGCACATCTCACTATGAGAATAGACTCACCATAAAATAAAAATA
GGACCCGCCATGTATGAGATGTGCTATATAAGATCACATTGGTAGAGGATTGGAATGTATTTTGTTGGAAGTG
ATCCGGAGTTGTGTTTAATGGGTAACTATTCTGTTGGGTAAACTGACTCCAAAAACCCAACACACGTGTTGG
ATTGGGTTTGGATCCTGCATTTACGGCAGCCTCTAATATGAACATGCCATGCCATCTCAATTAGAGTCTACTTT
TAGGGGCATTCCTTTTTTGGATTTTAAGAGTGGGAAAGTTGGTCAATGGGATTTTGGTTTTTAGTGACGAG
AATCTCTTATTCATTTTTCAGGTTCATGTGGGGACCAAGGAAAGTTTATATTCCTAAGTATCACTTATCCTAAG
TGGGCAAATAACACCTAGGGGGGAAGTTGCTAACACTTTAATGGGACAAAAATGCCCCTTTTAATAATTAAT
TTTTATGGTTAAAATGGTCATTACCTTATTTACCTAGGAAATAAAAGAACATGGGTAAAATGCACTTACCCGG
ATAAGAGTTTCCTACGAGAAACTTACCTAGGTAACTACATTTACCCAAGTAAATAACTTACCGAAAAGCAAAC
AGGGCCTTAATAGTAGCAACAAACTCTAATCCGAATTCATCTATCTATTGGAAAATGACCTCCCAACTAAATAT
GGATTTCTTATTCTTTTATCTATGTATTTTAATAAAGGACAAATTTCTTAAATTATCCCTATGTTTAGTTAATTACT
AACTTTACCCTTACTGTTTAACTATTGTCTTGCACCACCCCTATTGTTTATGACAATTGTCATGCCATTTTTCCTC
TCTCCTCACTCCCTTCTCTTGTATTATCATCGTTGTTGCAAGATCTGACGACAAACATAGGTAGTTGTAGGGGT
GAGCATTCGGTTTTTTCAGTTCAAAATCGAGCAAAAAAAACTAAAATTTTAAAAACCGATTTAATTATATGGT
AGACCGAACCAAAACTGAAATATATGAAAACTGAATAAATCGAACCCTTTTTTTTTTGTTATTTCGGATATTTT
GGTTAAAACCGAAATTATTTAACCTGAAAGATGCAACGTAGCGTAGGAAGATGTAGGTAGCCAACAACACT
CGGCAACGAATCAACGTCGTTCGTGTTTGGAGCCTATAGCGCAGGAAGAAGAAGAGGACCGAAGCCAGA

GGCGGCAAAAGGGGGCCATCGGTTAGCCACGGCACTCGGCAACGAGTCGACGTTGTTCATGACTTCGTGT
TCGGTGTTCCTACTAGTGTTGGGTGAGCTGGTGACGTCCAGTAGCTGTGGTAGTTGAAGAGACGAACTAAA
CTGATGTCCAGCAACCGCAGTAGTCAAAGAAACGAACTAAGGAAGATGCAGTCCAATAGCCGCGACACTTG
ACAATGAGTCAACGCCATTCATGACTTCATGTTTCGTGTTCCTGCTGGTGTTGGGTGAGCTAGTGACGTCCG
ACAGCCATGGTAGTTGAAGAGATGAGACTCTGGAGACTCTGGACTCTAGTCTGGAACTGGAAGACGATTAA
ATGAGAGTCCCAAGATGGCAAGTCGAAAGAGATAGGAATAGAGAGAAAGGAGAAAGGGTCTTAGTCTCTA
ACAGAGTGACAGTCTGACAGTCTGACTCTTAGATGCCCACATGACAAGAAAGTTTCTTTTCTAAGACATTTT
GTGGTTTGCTTATTTTGGAATTTGGCAAATAGCCAAATATGCTTCTTAATTTATTAAATATTAATATAAAATTAA
ATTATAAATTATAATATTAAATCGGTTTTTTCGGTTTTAACTAAATTATGCTCACCCCTAGGTAGTTGACCTGCTA
TCTACAATTTTCCCTTGTTGGTGAAGCATCGTTGTTAGTATCAGTTGGTTGTTCTAATGGAAATTACACTTCCG
GAGATTTACCCTTCCTCGTAGAGAACTAGGCAACCAGGTTTCCCAAATTGTTTATTCTCAAGGATCGGAGAT
CGAAGATAGCCTTAGCCCTCAACCTCCCCAATGCCACTCTTGCCCTGTCAATGATCAACCTCCTTGATGACTG
TAGTATTGCAGAGGTTTCTCCAACCAAACTTAGTTTTGGCCTCTACACTATGAAACCCGACCTATGACAATGC
ACAAGGCAAGGTGAGGTGAGGTAGTACCTCATCTCCGACTGTTGTGGTTGAACCATAGCACCTCATCACCG
ACGAAACCAGAGATGAGGTAGGTGATTAGGTGAGGTGGGGGAAGGAGATTTGGTAGTAGTGACTGTTGTG
TTTGAACCATAGTACCTAATCACCAACTACTATGTTCAAACCCTAGTACCTCATCACCGACTAAACCATAGATG
CAGTGGCGAGGCAAGGCGGGGGATGGAGGGTTTGGCGGTAGTGAGGCAAGGTGGGGATCATGCCCCAA
CTCTACTAGGCTCTCTCTTTCTCTCTCCTCTTCGTCTTCTCTACAATCGAACAAACCAAATAGGGCTTTCTCTCT
TCTCTTCTTCATTCTCTCTACAATCGAACAAACCAAACATCACTTTCTCTCTCCTCTTCTTCGTCTTCTCTATCGT
TGAACAAAACCAAATAGGGCTTTCTCTCCCATCTTCTTCGTCTTCTCTACAATCAAACAAACCCAAGTAGGGC
TTTCTCTCTAATCTTCTTTGTCTTCTCTACAATCGAACAAAACCAAACAAGCCTCTTCTTTGTCTTCTCTTTCAA
TTGAACCCTTTCTTTCCTTTACTCGAATCCAAGAGCAAGATCATCAAGACAGGGGGCTCAACGTTCATCTTCA
TCATCAAGATAGTGGGTTTTGCTCTAACGGAAGATCTAGGGCTTGTTTGGCGTGTCTTTCTATGCGGTCTTTC
ATCTATTTACAACCCAGGAAGACCGATTTTTGGTCTATTTCCATTTGGTGCGCAGAAGATCTAGCCGCCAAAA
GACGGAACGCCAAAAATATGCGAAAAGCCCATTTTTAGCTGATTTCGAGAAGCTTCAAATGAAGCTTCTTGT
GTCTTTCACATTTTAATCTCGCACGGAAAGAGACGTGATTGCCAAACATGCTTTGGGCTACTCAAATCTTCAT
ACAAAAGCTCAATGCATCTTTTGACTGTCAAAGCTACCAGAAAATCTTCCCAAAAAGACATGCCAAATGGGC
CCCTAGTTTTAGCCTATGTTAAAAAAAAATCTTTCTTTGTTTCAAAAAGAGGAGAAGAAAAAGGAGAAAAG
GAAGAAAGAGAAAGATAGAGGGAAGAAAGAGAGAGATTGGATGGGGAAGGGGTGTCAATGTTATGTCAC
TACGTAAAGAGGAAGGAAACAAGGGTAATATTGGACTTTAAAAAATTAAATGTCACATATGTACGAATTTTG
ATAGAATATTAATGGATGCTAACAATAGGGTTAGAGCTAGTCAATTGTCAGAAACAATAGAGGTGGTGCAAG
ACAATAGTTAAATAGTAAGGGTACAATTTATCAATTAGTTAAAAAGTAGGGGAAATTTAAGAAATTTTCCCTT
TAATAAATTTTGTTTGATACTCGTTTTTTATTTTGTAACTTTAAATCATTACTATTTGATTTTTTTATGTATATATTT
ATTTTTTTAGAAAAGCCTAACAATGCATTCAATGGCTAAAATAATCATATACAATGGAAGATCTAAGGAAATCA
TTTGGGGACCATGTGTGAGGACAATTTAATGTAAGACCAAATTTGGCAAGTGTATGTGCCACAAAATTCATT
GATCTAGGAGCATATCTAACTGAGCAACTAGGGAGGTGATTGAGAGAATTTCTGTAATAGGCAATTATAGCAT
GAAGATGCTACAATGGTTTAGAGTATACCACCAAAGTCAGTAATAACTTATGCATCACCTTCAATTATAGTAGT
TGATTAAAACATTGATCTAGAAAATGCAATAATTTAGACAACGCTTTTTTTTTCAACAACAAATGAAGAGCAA
TGGGCTATAGGCATAGCCCGAGCAAAAATAGAAGCACCAAAGTGATTTCTTGCTACCCAATCTAACAATGGA
TAAATTGTGCTTAACTATGGCATTAACATTTATCTTGATCACATCAATCCGTGGTGGGGTCAAATGTAGTTGTT
GGCTTGGTGACAAAGGAATAGTAGATGTTTGAGTAGGAGATATTGGCAAGGAGAACACATTATCCATGGTT
GCATGAGATTTGGACATAACCATTACTAGATGTTGTTGAAAGTGCATTCAACCAAAAAATCTCATAACCCCTA
GGCTTTTATGGTAGGCTTCGGGTTGTAAGCAATGTCAAACTCTTGTAGCTCTATAACCCACTTTGCTAGCCAA
CCCGAGGTCCCAGGTTTTTGCAATATTTGCTCTAGAGGTTGGTTGGTTAATACAATTATAGTGTGAGTTTGGA

AGTAGGGTTTGAGTTTCCGACCATTTTGAGACTAGTGATGTGTTGATTAGTTTAGAGACTCTTCACAACCATG
TCATCGACATAGCCTTCAACGTAGCGGTTGATCTGTTCCTTGAATATTTTATTAGCCAGCCATTAGTAACTGGC
ATCGGCATTTTTCATCCCAAATGGCATCACCCTATAATAATAGGTGTCATGTTCTATGATGAAGGTAGTCTTCTC
TTTGTCATCGAGGTGCATTATGATCTAATCATAACTGGAAAAAACATCCATGAAGATGGAGAGTTCATACCCT
GGGGTAGTCGATGATGCAATCATTCTTCGACAAGGAAAAGATGTCTTTGGGGTAGGCCTTATCGAGATCTAT
AAAATCAAAGCATATTTATCATTTCCCATTAGCCTTTCTCACCATATCCATATTAGCTAATCAATTAGGGTAGCAT
ACTTCGCTGATGAACTTTGCTTTGAGTAATTTATCCACCTCCTCTCTGATCATTTTCCATTGCTTTGGTCCGAA
GTTCCTCTTCTTTTGTTGAACTAGCTTATAGCTCAGGCCACATTAAGCCTATGAATAATGACTAAGAGGTCAAT
TCTTGGCATGTCAGCAGCAAACCAAGAAAATACGTTAGCATTGGCTTTGGGGAAGATTACTAGTTCAAAATG
TATGTCCTTAAGGAGGGACCCAACTTGTATTGTTTTCTTGGGTTTGGCCTCGTCCAAGTGTACAACCACAAG
GTCCTCTATAGGTTAACCTTGGATGGCTGGCCCATCTTTGAGGTCGAGCCTTTCAAGAGGTAGGATTCTTTG
GTTTTTCCTTTTAGAGTGGTGTGTCGAGGTTCAAGTCCTACCATGAGAGGCAAGGTAATCTTCCCTTCTGATT
TAACTACTTCCCCAAAGAAATTGACCAAGGAGATATCAGTGGGCTAAAGTCAATTCGGGGACAACTTCATCT
TTTCAAAGGCCTCGTAAAATAATTAGTGATCTAAGGAGTTGTTATTTACGAGGATCCTTTTTACATCAAAATTG
AAGATCATGAGAACAACCATCAATGCATCATCATGGGGGTGACAACCCCCTATAAGTCATCGTGAGAGAAGG
TGATAATCTCCCATTTGGCTTTCTTATAAGGCTTGTGTCCAATCTTGAGTTATTGGGCATAGGACTTTCTGGCA
AAAGAGCTTTCTCTACCCAATGTTGACTCCTAATATGACATTAATGACCCTAATAACAAGGCCTAAGTGATTAC
AGTACTGCCCCAGCAGGCTACGGTTGTGCCACATGTCCTCATATGGTTGCATGAGTGAATTTGGATACATCAC
CTATAAATAGGGGTTCCAATTCCCATTTTGGGAGCACCGTTTTGGACCACTATTGATCCATCACCCTTTCGCTA
GTCCATTTCTCTCTCTCTCTCTCTCTCTCTTCTACCATTTTCCCATTTTCTCCCTTTCTCCTCTACTACAAAAACTT
TAAGCCATCTCCCCAAATTTTAGGGTTTAGACCACCATGTCAATTGGCCTTATACCAAGTAAATAAACCAATCA
ACCTCACATGGTTTCTGGTACTTCCTATCAACATAGTTCCATGGGAAATTGTCACTGAAAAATAGTTGCAAAG
AACACCATTATCATTGATGATACAAAGGTTCTCTACTAATCCTCTTTGTAGTTATTGACCAAAAAAGAAAGGG
GTTTTAGACTACCACCATTTTTAGAGAAGATAAGTAATTATAGTTGTTGTTAGGTTAGAAAACAAGCTAAAAA
CTATAATTTTGTCCTATGTTTAGAAAACTAAACCACATCGATCGATCAAACCATGCCACGACTTGGACTTATGC
TCGGTTCAATTGTACCATAGAAATTGGCCTACATAGAAAATCGCTTAAAACCGGATGCAAAATGCAAATAATA
AGATTTGAAACCATGACCTTTTCTTCATGTATATTGACTTATTACCACTAATCCACTTCATTATTTATGTTCTCGG
GCTCATCCTCGTCCAAGGATATAGTCATGAGGTCCTCTACTGGTTGGCCTCGACTAGTTGGCTCATTTATGAG
GTTGAGCCCTTCAATAGGCGGTGTTTTTTTCTATTTTTCCTTTCAGTTCTGCAACATAGCATTACCTTGATATCT
ATTAGTCTCCCCTGACCACTTCAATGCCATATTCCATGGGGAACTTCCTCACTAGGTGGTATGAGGACAACAC
CACCTTCATAGCATGCAATGTCGATTGAATGATAATTATATTATATGGGGAGCCGAGCTTGACGACCAAGAAC
TTGGTCAGTAGAATGGTTTGTTAGGGCTTGGCCAGTACCATGAAAGATAGGGTAATCTTCCCTTTTGGTTTAA
TTATTGCCCCAGAGAAATCAACTAGGAGGACATCGGTGGGCTGAAGTCAATCCAAGGATAACTTCATCTCTT
CAAAGGCCTTGTAGAATAATATGTCAGTTAAGGAGTCATTGTCTACAAGGATCCCTCTTACATCAAAATTGGA
GATCGCAAGAGCAATCACTAGTGCATCATCATGGGGGTAACAACCCCCTATATGTCATGATTAGAGAAGATGA
TAATCTCCCTTCAGTTTGGCTTTCTTCTAGGGCCTATGTCCAATTTGGAGTTGTCAAGCATAGGGGTGTAAAT
GGGTTGAATTCGAATCGGATCAGGCAATATCCGAATTCGAATCCTATTAAATATTTGAATATTCGATTAGATTC
GGATTCAATTAATTAATTATTTTAGATAATTAATAACTATATAAATAAATATTTTATTGATATAAAGTATATAAATAT
ATAAATTATTTATATAACTTAATTATTATATATAAACTATATAAAATATTTTATATATTATATATAAGAATTAAATAATA
AGTCAAATTCGATTCGATTATGTAGTATATCAAATTCAAAATTGGATTTTTGAATACCGTTGACACCTCTAGTC
AGGCATGGGCCTTTTTGGAAAAAGAGCTTTCTTTTCTTGATGTTGAACCTCCTTATATGACATTAATGACCCC
AATGAAGAGTTTATTAGTTGGGAGATCATCTAGCTCCGAGGTTGACCCTACTATTTGATCACCTAATATGAAG
TCCTTCAAGTAGCCTTTCTTGATGATGGTCTCGATCTTGTTTTTCAGCTTGCAATAATCTTTTGTATCGTGGTTA
ATGTCGTAATGATATTTATAGTACTTGGTTCCCCCTTTTGGACTTCTTCTTCATCTTAGAGGGTCATTTTATGAA

CTCATGCTTCTTGACCTACATGTGGATGTGACTTTAGGTAACACTTAGAGGAGTGTAACTATCACTTGAATAT
GGGGCATGTTTGGCCTTTATCTTTCTTATTTTTGGAGCTATTAGGCCTCAGCTCGTCTCTGTCCTTCCACTTCA
TGTCGGTCAAGTCATCAGTCATCGGGTGTTTGTTAGCCTCGACTTCTTCAACATTTATGTATTTCTTTACTCTCA
TCAAGAACTCAGCCATATCCCAAGGTGGCCACTACAAATATCGCTTGATCGAGGTCCTTACCTTCAAGCACCT
CAAATCTCTATTTTATGGCCATTAGTGAAACCGATGTCTTCTAGCATCAGCAGTTGCTCACAAAGTGAGACAT
TAAGAGGTTTGCAAGCCAGTGTTTTAAAATTGACCCAGAGGCCAAACTATTTTTGCCTTTGGGTTGCGGGT
CATTGATTTGACCATCTAGGTTGAACGGGTCAGACCATGCCACGTTAGCATCTATATATTTTAAGTAATATATTT
ATATACATACATACATATACATACATACATACATACATACATACATACATACATACATGTATTGTGTGTGTATGTACA
AATGCATATTTTATCAATAATTAATGTATAACTATGTTTTAGACATGATATATGTATTATTTTATATTTAATTTATGT
TTTCAGTTCAGTTTTTATTTTTTATTTATAAAATATTAAAAATTGCAATTTGAGGCCAAAAAAGGGTTTTTTTT
TTTTTGCAAAAATAGCGGAGGCAAGACACCTATGGCTCCTTAGGATGCAGAGATCTCAAAAAGTTACCCAA
AATTTAAAAAAAAAATATCACCTCAATCAAGCATCAAAGTGCAAAGTTATGGCCTCCCGAAGTTTACATACATA
GTATGCAACCCACAAACCAGTCACCAGGTCGACCAAGTTAACAGTCCAACCATCTGGGTTAGAAAGTTTTT
GACCATGTGAATGGGTTTTGACCTTTCTCCAAGTTTTACTCAAATTGGACCCAAAATCTTGATCGGGTGATAG
TTCGAGTGGTTCAACCGGTCGGGTCAATGGCAGGTTGAAAACACTGTTGCAAACTAGTCCCATGAGATGAT
GGACCATGGGTGAGACCTGAGAACCATCTCCTAGTGGTGCCCTTGAATGTAGTTTGGAAAGCTCTACACATT
AAGGTGTCAAAGAACCCATGAAAAAGAGCATGATTCTGTAGAAGCTCTCAAATTGGGAGCCTTAAAGTTTG
AGGGGAGAGGCTTTGTCATGATGGTCAGTGAAGGCCTCCATCTGCCTCGCTGAGATGTGTTATCTAGTTAAG
GCATCAGCTCTACCTTCTTAAAATTGGTAGATTTCAACTCGGATAACAACAAGCTCTACTACAATGTCATTAAA
TCTACAGTCGTGATCCCTAAGGTTGTGCTTGGTTTGAAGGAAAATGTAGGAAAAGGAAAGGAAAGATAGG
GAAAGCTTTGCAAACCTTGTATGGGTTTCCCTACCTTTCCCTCCCTACTTTTTCCTTCAAACCAAACATAGCCT
AAGGGGGATCTTGGTAGGGATGTCTTCCTCACCCATGAAAGGTTTGAGTGGTTTTGGAAGTGTCCCGTATC
AACTAGAATGTTCCCTCCCCGAGTCCAATTATTGTTTAGGTGCTCACAGAGATCTGATACTTTCATAGGAGTG
AGCTACTGGTCAGCCTCATTTTTCTAACTTGGATTCCATCATTCTTGATGTTTGTTAGTGCTCTTATGTTGGCCC
AAAGGAAAATGACCAAAGTGATATCTACATGTGAGTACGGGAACAACGTAGACTCACCAAATGGCTATTTCT
CTTGGTTTTATAGTGGTCATCCCTGGTTCATACTCTAATATGTTGGCTTAGCAAGGCCTTGGCAGACTTCCTAC
CCTGATGCCCCCTAAGCATCAATGTGTTGACCGTGTTGGGGTTAGCCTCTTGTTGGTTTTTGTAAGTTGTCCA
GACTATTGGGTCCTAGGATCCTAGGGAGTAGGAGGAGTGTTGTTTCTCACCCTGGTATTATCTTGGTGATTGA
TCATAGTAAGTGAAATGGAAAGTTTGCTAACAATCGTTCTCCCCAAAAGTGCTAATTGATGTTGCCAAAGATA
GAAACAAAGTCAACATTGAAATTCCTAGGGTTATGAGCTTAAGAGTCCCACCTGCAAGCACATAAAAGACG
TCAGACATCCCGGGGTGTGGTCCCAAGGGTGAAGATTCTGATTGTCAAGTCAGTTGAGGACTCAAAGAAG
CAAATGTGGTAGGAATAATGATAGTTAAGTGAGAAAGAATAAGAGCGTTACATGAGACCTTGAGTCTATACT
TATAGTCGAAGCCTCCCCTACGTGGGCATGATGAGTTCTTGATAACGGCTCCACTTGTCAAGCACCATTAAGA
ATGGGAGCATTAAGGTGTTGACTACATGACCAAGCATTACACTACCAAAAAAATGCACTAGAGAGGGCTGG
GTTGAAGACATAATACTTGTTATTAAGGCTCCGCACTAACCTAAGAAGGACAAGATGGGGATGCCCCTGAG
GCCTGAATGATGACAATATTTTCCCTATGGACATGCCACATGTCCTCATACAATTGTAGGAGTGAATTTGAATA
CATCACCTATAAATAGGTGTCCCAATGCCCATTTTGGGGGACCCTTTTGGACCACTATTGGCCCATCACCCTTT
GGTTGGCCCATTCTCTCTCACTCTCTCTCTCTCCTATCATTTCTTCCCCCATTCTCCCTTTCTTCTCTACTACAAC
TTTGAGATATCTCTTCATATTTCAGGGTTTAGACCACCACGTCCATTGGCCTTACACCATGTAAATAGACCAAT
CAACATCACATGGATTTTGGTACTTCCTACCAACATAGTTCCATGGAAAATTTTCACTGAAAAAGAGTTACAG
AGAACACCATTATTAGTGATGATACAAACGTTCTCTACTAGTCCTTTTTATAATTTTTCACTTGAGAAGAAAGT
AAGGATGGAAATTCTCCCCACTAAACCGAGGCATCGATTCGACCCACCTCGAACGTGGTAGATTTTCCCCAG
CCTTAAATGGGAACAAGTTAGGTTTAGCAATTTTCTTTTACCTTGAACTAAGGATGGGGTTTGCATGTAAATC
CCCAAACCCGCCTTGAACCCGGTGGTAAATTGCATTATTACCCTTTATATATGTAATATAGGTGTTGAGTGATG

ACATGGCACATGGCTCTTAGTCTTAGGTTTTGGACCTTTGTCTTTTAGTTTCCAGACTTCAACATTTCCCCCA
AATCAGTCTAAATTCCAAAACCCAAAACCCAAAAAGTGACAAAACCCTTCCCTTTGATAGTCATAAAGGAAC
TCTCAAATGGACAAATCGAAAAATTTGAAATCAATTCTTTAAACTTAGTAAGCACAAGTGGACGGGTTTGAA
ATTGCAAGTAATAGCAGCAATTTTGTCTTCTAGAATCCAGAATCGAGACTCTCCTTTGTTTGGATGGTGGACC
AATGATTTGATTGTTATCTCATCTACTTCTCCCTACCTTTCTTAGTCTTCGTTATTCAAAGAGATGAGTGATTTTA
TTAACCCATCTAATAACAAATTAATAATGAATGGATGGCAAAATAGGATTCATGTAGTGATAATGATCTCCTAA
AAGAAAATAGGGGATAGTCAATATTGGTCTTAAGGAGTTCGTAGGCACTAGACATATACACCTAAATATTCAT
ACTTATTTTTTAAGTTATTGAGATGGTGAAACCCAAGAGTAGATCCCCTCTTTAAGGTTCTTTTGTTATTTGTT
TACTTTTCATATATAGAAGTTTCTCATATTTATTTTTTTAAGTTATTAAGATGTTATTTATTTATTTTAATTTAGGTC
AACAAGTCTTATATGAGCCTTTCATTTCTACTATATTATTGATATACTTGTGATCACTACAAATTAAGAAAATTAT
ATTTTGTACCCAACATCTATAAACTTTTGCATGACAATTGACATGTGCTCATTTACATCAAAACCCAATTCTTAT
TCATGATTCTAGAAATAAACTTTTGCATGTTAGTTTCTATTGTACGTCCTTCTAGATATCTTGTTGTAGTAACTTA
ATGTACCTTGCTTGTCCTGGCCATAGATTTCACATTTTTCCTTTTTACTTCTATATGATTTCCATAAGGTTTTAGT
CAAACATGAAATTATTTTAGTCACATTGGTTCAATCTTTTATCATCATTATCTTGTTTACTCTTATTTGCCATGCT
TTATGTTTGAAATTTTTGAATTACCGTATTCTTTTCTTCTTTAGCAATAGCCTTTTCCAAGGTCAAATTAGTTCA
TTATATCTCATCTTTGATGTCAACTTTCAATCCCAAATGATACACTTAGCCAAATTAGTTCATTATATGAATTCTC
TACTAGTTAGCCAAAGTGTTATCATATCTCTTGCTTTAACCCTAAATGATAAAATTACAATAGCTTCACCTTTAA
ACTCTAGCAATGATGTTAGCAAAGAAGGTTTAAGTACTAAAACTTCAGAGACACCATCTAAGAAAAGAGTCA
TATTTATTACATGAGATCATTTAAAAAAACAACTATTGAAGGATAAGTCGAGGCGGATTCTAACTACTATAATA
AGAAGCTTGGTAGGGACTCAAAAAATGGAACCAAACATTTGCATTAGCATTTTAAAAGGTGTTTAAGGCGT
AATAAACAAAATATAGGGTAACAAATTTTGGGGGACAAGTCAAAATAAGAAAGATGAAAAGTTTAAGCTCA
CATACATCACCTTTAATCTAGAAATTTTAAAAGAGAACTTGCTAATATGATTATTTTATATGATTATCTTCTTTCT
ATGGTCAACCACATTGGATTTAAGAGGTATTCATATAGTCTTCAATGTATTTCCATAGTGGTTTCAAAAAACAC
AATTAAAGATGTTTTGAGGATTTTTTAGCAAGAAAAAAAAAATATAATTAAGTTATTGCAAAGGAATTAGAGC
AGAGTAGCCATCACAACTAGCTTGTGTATCGCTAATAATCAGAAAAAATGTTCATGAACATCACAACACAAGT
CATTGATGTTGATTAAGTTCTACAAAGTTGAATTATAAGGTATGTCTTTTAATATTATAATTCATTACTAGTATTT
TGTATTCTTAAGTAAGTTATACCATGTTTTATATTTAGTATTGTTGCTTAGATTTTTTCCCTTCTAATTTTGTTTAT
ATAACTTATCTATACATAAAAGAGGTTTTTGTGGATATGTTAGTGGGATATTTTTTATATTGGACTATGATAGAA
GGTTATTTTTAATTGTTATTGATAATTATATTACAAATGATGTAATTTTCAACGATATTATGGATGAACTATCTTG
AGTTCACTTATGCTAGATGAAAGATTCTTGCATATGTGTTGATGAGCACATATATTAAACTTCATTGTACAAGA
TGGACTACTTATGATTGAAGATGCCATTGAGAAAATTCGTAATAATATTGTGTATTGAAAAATAACTCTAAAAG
AGAGTAAAAATTTGAAAAAACATCCCAATAATTACAAATCAAAGTAGGAAAAACTCACTTGATTGTAAAAAT
ATATAAAAATCAACATATAAAATACTTCATATTGCTTTGATTTATAAGGATATTTTCTTATTTAAGACAATGTTGT
ATCACAATATAAATGTTTGCCTAGTAAGTGTGGAAATTGGAAAAAAAAATTGTAAGAGGATAAAATTGTTTTAT
AATGTACTAAATTATCATCAAAGATTAAGTATCTCACAACAATTTTATATTTTCCAAAAGTTTATGAGGTCAGAA
TAGTATTATCTCTATAGTTTGGATATTTATTTAGGATTATAAAGAAAATGACATTAAAATTGTTCGAGAAATTTA
ACAAGTATGGTGTATAATTAATGAAATTTTGGGTGTAGCATCAGTGTTGGATCCATGATATAATATAAAATTAAT
AGAGTATTACTTTCTTATGATATATGGGGATGGTGTATTAGATGTGATTGAGATGACTCGTCAATTATGTTATGA
CTTCATGAGTACCAAACCAAATCCAAGAATAAAGAACTTAGACATAGTAGTAAAACTTTAATTGATCTAACTTA
TACATCTGAAAATGTTGATATGGAAGTACAAGATCCAATTTTCAATTATGAAGTTAGAATTTGATCATTATTCA
GAAGAAAATGTGTTGCCTAGAACTCAAAGTTTCGATATTTTCGCTTAGTGTAAGTCAAATGGATTCAAGTATC
TTACCTTACAAATAATTGCCAAGGAAGTGCTAGCTATTCCTATTTTAATAGTTACTTTAAAGCCTACATTTTTCA
CTGGGAGAAGGCTACTAAGTCTACATCGTAGTAGACTTCATGTCAAGACATTAAAGGCATTCATATATGCTTA
AAATTGGTTGGAATATGAAATTAAAGGTAATGTTATTTAAATTGAAGTTTTATTTATAAAAAAAAATTGAATGTT

CATCAGTTCAGCTTTAAAATTTACTTTCTTCTTTTATGTAGATGCTCCTGTTGGTGTAGATCCTTGCACTTATAA
GACTATCAACAATAATGATTTTGATATAGATGTTGAGGTATATGATTTAAAATGGTTATTATTGAACATAATAAG
TAAAAATAATTTTTGACTTAGTCTTCATATACTTACTCTTGCTTTTTAATTTAAAGTAATTTCATATCTCAACAAA
CATTATCAAATAACTTTGTATCTCAAGTCTCAACAATTCACAAAAAACAAACCATTTATCTTAATCAAACAATTT
TCTATTTTAATCCACAACCTAAGTTTCATAAAATAATTAAAAGGCAATAGTAAATATATAGAGACTAATATAAAA
ATGATTTTGACTTATATATAAAATTCAATAATTACCATTTTAAATCATATAATTCATCATCTATATCAAAATCATCAC
TATTGATGGTGTCATAAGTGCAATGATCCTCATCGAGATGTGCATGTTGGATTAACTAAAGTTTTACTACCATA
TCCAACTTCTTTATCTTTAGATTTGGATTAAAACTCATAAAATAAGTCATAACACAATTGATGAATCCTCTCAAT
TTTATCCAATACATTATCCCCATATATTATAAGAAAATAATATTCTATTAATTTTATCTTATATCTTAAATCCAACAT
TCCTACCAAACCCAAAATGCCATTAATCCCACATTAATACTTGTAAAATTTCTCAAACATTTCATCCAATGCATT
ATCCTCATATATTATAAAAAAATAATATAAATTTATCATGAATTTAATCCCCAAGAATTATCCAATCGCATTATAAA
ACAACTTTAACCTCTCACAAATTTTCTTTGCCAATTCCCACTTTTCCTCACTTTGTAAACATTTATGTTGTGATT
CACATTGTCTTAAGTGGGAGAAAACATCCTTATAAATTATAGTAAAATGAAGTATTTTATATGTTGAATTCAATC
TATTTTTACAATCAAGAAATAATTTCTTATTATCAATATTTTAATATAAAAAAGGACATTATTTGTAATATAACTAT
CAATAGTAATTATGGACAATAACTTTCTATCAATATTTTAATATAAAAAACATTCTACTTGCATATTTGCAAAAAC
CTCTTTTGTTTGTGGGCATGATACATAAATAAATCTAAAACAATAGATTAGAAGGGGAAAAAAAACTAAGCA
ACAATACTAAATATAAAAAATGGCACAACTTAAGCATAATAAATGGCAGTATTGAATTATTATGTTAAAATGCAT
ACATTATAATTAAACTTTATAGAACTTAATCAACATCAATGGAATGTCTTATGATGGTCATGATCCCTTTTTTCTA
ATTATTAGTGGTCCACGTGTTAGTTGTAATGGTTACTCCGCTTTTATTCTTTTCTAACAACTTAATTATCTTTTTC
TTCTCTTGCCTAAAAACCTTTAGAATATATTTTTTAATTATTTTTTTGAAACTATTGCAATAATAGGTTGAAGAC
CATATGAATACATCCTAAATCCAGTGTGATTGATCATAGAAAGATGATGCTCATATAAAATAATCATATTAGCAA
GTTCTCTCATTGAGATTTCCGGATTAAAAGTGGTGTATGTGACTTTAAACTATCCATCATTCTTATTTTGACTTG
TTCCCCAAAGTTTGTTGCCTTATATCCGATTGAATATGCATTGAACACCTTTAGAAATGCTTATGCAAATGTTT
GGTTCCATTTTTTATTCCCACCAAGCTTCTTATTATACTAGCTACAAATTGACTTAACTTGTCATTCAATAGTTTA
AATGATCTAACATAATAAATATGACTCTCTTCTTAAATTGTGCCTTTCAGGGTTGTTAACAAACAATGTCAAATT
GACCAAACTAGCCTGACCTAACCCGATTTGGTTGGTTTTTTATCACCACTAGTCAGTTTTGGGTTGTGAAGA
GGAATAAATTGATAATTTGGTTCGGTTTCGATTTTCATGTGTGAAAATTGAAACTGAACTAAACCAAACCGA
GTTAATTAATTTCTAATTTCTTAGTCTTTCCCNNCTTTCCCTAACTAGGTAATCCCTATCCTACTTTCTTCTTTCT
AGTTAGCTACTCATTAAATAATAACAAAACATTCGCTAAATGGGAATTACTTTTGTCTTTAGAATTGTTTTCCTT
TTTTAAGTAGGAAATTTTACACCAAATAGGAAACATCGAATATGGCTATGGGGAGAAAAGACAATTTAAGTA
AATTTCAAAAACCATTTTGGGATTGGCAAGCATTGCAAGCAAGATCCCGACACCTTTAAACTTGCCAAATTT
AGAAAATTTCTCATTTGATGGGAGTCTTGGAGAGTTGACACATTGGAGTCTTGGCTTCTTGCATTTTTTGAA
AGAGTAAAAGAGAGATTCTACTTTCTAAGGTATACTTTACACCAAATAGAGAAATAGGAGTCTTAGAGTTTT
GAATCCTGATTTTTCATTGGTTCTTTCAAATTTCAAAGCTTTACCATTTCCTACTCCACCTCCTCAAGCACTCTT
AAGGTTTCTTTTAAGTTTCATCCACCACCATTACAACTACAAATCTCCCATCTTCTCAACTCTCATTCTCTCAAC
TTTCAACTTCTCAAGACTCGAGCATTACATTCATAGGTTAGTATTTTGTTTTTTATTTAATATTTTAAATTTTAAT
GCATGTTAAAAGTTGATATGTTTTATTTGTGTTTTGTTTAAGCTTTGAAAAAATCTGGTTATTTATGATATATTT
TCACAAAAAAACTTTAATTTTGTATGAAAAACCTAAACTATTTAAATATATATTTTGATTTTAATTTAAATATGTA
TTTTATATTTAAAACTATTAAATGTAGATATTTCAGTTTTGCATAAAAACTGAAAAGAAATAAACTTAAATATAA
ATAGATATTTAATTATTTAGATATAGATATTTTAGTTTTAAATAATATATATATATGAAAAAATATATATTCCGAAAT
AAATCAGATATATATTTCAGTTTTATAATATGTATATTTCTGCTATATATAGTTGAAATATATATTTTGGAATATATT
TTTCATATATATATATATATATCAGTTTTAAATATATTTTTGACATATAGTAATACATATTTATATTAATTTCATAATTT
GCACAAAAGCTATATATTTTTATATATTCTGAAACATATTTTTATATATTGTAGTTTTATTTATGTTAGATAAAAAA
TAATATTATAGTTTTTTATTAAAATTAATTAGTTACTTTTTCCTTTACTTGTTTCATTAGGGATATTCATCTTAAGA

AACTAGAATACCATGCACATTTTCACTAAAATGGAGGATTAGGATGAACCTATGGTTGCATGTAACTATTGTG
ATAAAGGTTGCACATGTGACACAAAAAAAATTATGGCACTAGTAACATGTGGAGTCATTTGTAGCAATAGTG
CATGCAATATCCTTATAGGGTTGATTAAAGGCAAAATACATTGTCTTTTGAGCCTAAAAATGAAGGAGGAGAT
GATGGTCTCACTACTAGTAAGTTAAAAGCAATGACATTTAGTGTTGAAGAAGATAGAAAGGAGCTTGTTAAA
ATGGTTATAATTGATGAATTACCTCTTATGATTGTAGAGGGGGAAGGATTTAAGAACTACTCTCGAGCTTTGC
AACCTAAGTTTACAGTCCCATCCTGTATCATAGTTTCTAGAGATTGCATGAAAATTTTTCTTGAGGAGAAGAT
GGAACCACAAAAAGTGATGAAAAACCAACGTTTATATCTTACCACTGATACTTGGTCATCAATCCAGAATTTA
AATTATATGTGCTTGATTGCACATTGGTCATCAATCCTGTATCATAGTTTATAATGATGGTGCAATTAGATATTTA
AAAAGAACTAGGGATTGAAAAGGCACCATCTTAGAACATGAATTCATACATTTAAGATGTTCTGCTCATATTG
CGAATTTAATAGTGAAATAAGGATTCGAAGGCTTACATGAGTCTATTCTCAAAATCCAAAATGCAATAAGATA
TGTGAGATCATCTCCATCTAAGCTTCAAACTTTCAAAAAGTGTGTTAAGAATAAGAAAATAGAAAATAAGAAT
CTTGTATGCCTTAATGTTGAGACTATGTGGAATTCTACCTATTTGATGTTGGAAGTTGCTAAAAAAATTGAAA
GAGCTTTTGACCGATTAAGGGAGGATGACCCAAGATATCTCTTATATTTTGAATATGGTGATGAGAATGAAAA
TGAGGAGAGTGGAGGTGGGAATAGGAGAGGAAAAGGGAGTGAAAATAAGCATGTTGGGCCTTCTAATTAT
GATGATTGGAACAATGTCGGATGCTTTGTGAAGTTCCTAAAACTTTTCTATAATGTATCATTGTGGTTTTCAAG
ATCTCTTTATGCCATATTGAATGCATTTTTTCATGAACTCGTGGCATTGAAAACTAGGTTGGTTTGTTTGTCTA
AAAGTGGAGATAGTCTCTTGAGTTCTATGGCAGAGCGCATGAAATGAAATTTGATAAATATTGGGAGAACAT
TGAAAATCTGAATTTCTTGCTATATGTTGCCATTGTTTTGGATCATTGTTACAAATTAAAATATGTGAAGTTTTG
TTTTGGTCAAATATATGATAGCTTAAGGGCTAGAGAGATGAACGAGAGAGTAGAGGCAACTCTTAATTGTTT
ATTTTACCATTATCATGATCATGAATGTAATTTAAATTCTAATGCTAGACATGAAGCATCAAGTTCAAGTAAACT
TCTAATGAAATGGATTTTGATGATAATGAAGATGATACATGGAGATTCTTAGCATCTCAATTTGCAAAACACTT
AAAAAATGTAGAAAACAAATTTGAGGTTACAAGGCATTTGTTAGAGAGTTATGAAAAACCAAGCGATTCATT
TGACATTTTGATTTGGTGGAAAATGAATTCTTAAAAGTATCCTATACTTTATCAAATAGCAAGGGATGTCTTGG
CCATGCCAATTTCTATAGTTTCTTCTAAATTGGCATTCAACACAAGTAGGCATTTACTTGATCCATTTAGGAGT
TCACTATCTCCAAAAATGGTGGAGGCTTTAATATGTGGCCAAGATTGGTTACGGTCCAAATCAATCCCAATTG
ATGTTTAAGAAACTATGCATGAAGTGGAAAAATATGAAAAAAATCACATAAGATAATATTTGTGGTCTTGTGA
ATAAACCTTATTTCATGATATTATTTATAACATATATAATTTTTTAGGTTACATTCTAATTTGTTTCTTTGATTTAG
AATTCTCGGTAATAATGAAACAAGTGTAATTAAGTAATGATGAATGATGTTTTGGTAAGACTTAATCTTTACAA
GTGTAATTATTTTTTCTACAATTTAAAATTTCTTTTTCCAAGTAACGTGACAATTTAAAACAAACTTTTCTTGTT
TGTTTCTTGACAGTTGAGTCATTGGTAGCATTTTTTCTTCAAACATGAAGTTACAAAGTCCATTAGGCCACCT
AGTTTTTGAATGACTACAAGAAATTTTGGATTAGTGATTAATGAACGACTTTTGTGATCATTACTTTTCATTTA
AATTTTATCAGTGGATGGCTGAAATTATGTTCAATTTATGACTATTTTTTTTACACATTACTTGTTTTTAAGTCC
AAAAGTTAATGATGTTCAATTACAGGTTTAAGTTTTATTAGAACTAGAAGCTTTATTAGAAGCCAATATGGCG
TTATTAGTGATGTTCAGTTACAAGTTTAAGTTTTATTAGTGATGTTCAGTTCAGTTTATGGCTGAAATGATGTT
CTGCTACAAGTTTAAGCTTTATTAGAAGCTAAGATGGCTTTATTAGTGATGTTTAGTTACAGGTTTAAGCTTTA
TTAGTGATGTTCAATTCAGTTTATGGCTGAAATGATGTTCAGTTATAAGTTTAAGCTTTATTAGAAGCTAAGAT
GGATTTATTATTTATTAGTGATTTTCAATTACAGGTTTAAGATTTATTAGAAGGTAAGATGACTTTACTATTATT
AATGATGTTCAGTTACAGGTTTAAGCTTTATCAGTGATGTTCAGTTCAATTTATGGATGAAATGATGTTCAACA
ACTTTAGTTACAAGTTTAAGCTTTTTTAGTAACCATTTTTGGAAAGATTGTGCTAGACTACTATTGTTACTGTA
GCTAAATCATTCAGCCCATCACAAAGCTACCATCCATCCAACGTTCTGAATATCAGATTAATTGAACCATCCAA
ATTTAAGATTGCATCACAACTTCACAAGCCCATTGGTCTTTTGAGTATTTATGAATGCAAAATGGAAAATGCA
GCCATAATCATGATGTGTAATGGGCCAAAAATCAAACTTGAACTGAAACTTATAGTTTTGGAATGCATTGGAC
CAAGTCACATTGAATCCATTGATAAACTGACCTATTAAGCTGATAAACTGACCCAAACCGACCATTCATGTTT
GGGTCGGTTTTGTTTAATGGCAGGTCGGTTTCGGTTTGCTTTTTAAGTAACCCAACCAGGTTGGTTCAGTTG

AACAAAACCTATTAAGACGACCCTGAAGGGATGAGAGATCCATCCTATGGACGCCCAAAGATTGCGGAATA
AGGGGCGAATTTCAAAAATTTTTCTCTCCCTTCAACTGGGAATCGTATTCCCTATTTAGAAAATCATATTTTCA
AGGATCAATCTTGAACCATAAACTGAACTAGATCTGAACCAAAAACCTATTTAGAAAATCGTATTCCCTATTTA
GAAAATCGTATTCCTGGGCCAAGCCACCTTGTAACACCCAGGGACTGTGGGCAGATGCAAGATTAGCCCAT
CAGGTATTATCCTCAATGGTCTTACACTTAACCTCTCAGCCCATAATGATCATACACTGCCCGACAAGAGAGG
ACCTCTCCCTCGGCTACAAGGAACAGTGTTGCACTCTCCACTCATATGGATCCCGGGAATTAAAACCTTTTTC
AAAATATTTGAGAAACCATAATATATATATATATATATATATACTTTTATATCTCATATAAAATATACTTTGCAAAAA
AAAAATCTCAACGATTGAAATCAGCTGCCGGCAGTCTTGGTTTCCATCAGTGAGAGTGCATCCCCGATCATT
ACCTTTGTCCGACAAAGGGGCTGATCCTGTCACTATGATCACCCACTCAGATGAATTCTCGGACTGTCCCAAC
ATACCCAGGTTTTGACCCCCGCTAAGGATCTCACTGATTGGTACATCAAAGACAGCCCAATCCGACACCTAG
TGCAGTATCATAATCGAGTCTAGGGGTCGGGTGTAACTTTCCAGTTTTGCCCATCAATCTCCAAGGTCCAGG
GCAATACTGGAAATCTGGTCCAGTTCAAAGATTCAATTAATCCTACACTGTATGTGCCTAGGGTCTCACCCTG
GCAGCTGAGACAGGCCACCCCAACCGACTTGGTGGCACCACAGTGTCAGCCTTGTCAGGATAGGTCTCCCC
AGACCGACCCACTGGCTGTGAACATTCTAAACTAACACGAGTCTCATTAAGTGTCCAGTTTAAGGGTATTAC
ACAGAAACATACCAAGGGAACATGAATCAAATATGTATCTTTATTCATTTACAATAAACTCATAAAGTACATCA
TTCTGATTGAAGCTAAGGGCATACAAAAACTGTCTAACAGACCCAACCCAACTCATGAACAACCCTAATGCT
TTTGAAGTTGTGGTACATGAACCTCTTTCGCCAGTATCATTCCTAGAGCTTGAACATAGAGCTACTATAGTTAT
ATCATTTAGGGTTGAAGCAAGAGATGGAATAACACTTTGGCTAGCTGGTAAAGAGCCCGATTATCCCCTTGT
CCAGACATCAAGACTAACTTTAAAGAAAAAATATTTTAGAAGTAGTTAAAAATGTTAGACAAATTAAAAAATA
TATAAAAAAATTTTAGAAATAGTAATAAGATAACTAGGTTTGCTAAAACTTAATGCCATCAACTATTTGTTGTTA
GCCTCTGATTCTTTGAAATGAAACCCACAAAGAAACCACTAAATCCATCATTATAATTTTTTTAAATACACATT
AAATTTTAACACAAAAAACAACATCTTACTAGTAACTAGGTTATATAGATCAATAATAGCAAATATTCTTCATTA
GAAGGAAAAAAAAAGAAACACTAAACTTTTCTTTTAAAGAAATAATATACCTTAAGACTTCAAAACTTGACA
TCCCAAGACTTGGATACCTATCTTTCCATCAAAATTCAAAAAAAGCAATTTTTTCTAGATACTGTGACAATAAT
CAGTCTACCTATCACTCTGATATTTGAAGGGAATTAGTAATAATGATGTAGTCATCATCTTGTTATTTAGATCAA
TTATCAATACTCTTTTTTCTTAGAGATGGTCTAAGCACTTCAGTGGTAGAGATAGAAAACTTAATTTTTTCATC
TTCCAATCAAACCAAGATTATCCAATTCATTGATAAAGCTATGATTAGATATTCCAAAGAGAATAGAAGAATGC
ATATCTATGGAATTGCTAGTTTATTTTATGTTCTTTAAGGTTTCATGTAAGTGTAAATCTTAAAAAATTTCTCAC
AGATATCTTTTAGATAAGAATTTAGCTTTAAGAAGAAAATCTTCATACTTGTTTACTAGTCTTTCTAATTTCATA
ACATTAAAATTTGGTTTGTCCACGTAGAATATTATACAATTGTACACCAGGAAACCAATAGGAGGCTTTTGTG
AGGAGGTTCCTCCAAATCTTGTTCATCACAAATATCTTGCTTTCTTTTACTTTTTCTTAGAGAGTTTTAAAACA
TGCATTCCTATAGGGTGCAGACAATATTATTATTTGCTTTTTGATTATGAACACAACAATGTTATAAACAAGAA
AAAAAAGGAATGCAAGTTGATATCAAAGATGAGACAATATTAACCAATTTGACCTTGGAAAATGCTACTAATT
GAAACCTTATGTGAAATCTGTGGCCAATACAAGTATGTTACATTAAGCTGCTACAACAAGATATCTAGAAGGA
TGTTAAGTAGCAACTAACATACAAAAGTTTATCTCCAGAATCATGAATGAGAGTTGGGTTTTGTGTAAATGAG
TGTAGGTCAATTGTCACGCAAAAGTTTATAGATACAAAATCCAACTTGTTTAATTTGTTGTAGTGATCGCAAG
TATATCAATAACATAGTAGAAAAGAAAGGCTTGTATAAGACTTGTTGGCCTAAATTAAAATAAACAAATAACAT
CTCAATAACTTAAAAACACAAATATGAAAAACAACTGACTATGAAAAATACACAAATAGCAAAAGAATCCTA
AAGAGGGGATCTAATCCTGGGCCTCACCATCTCAATAACTTAAAAATTAAAACAAGTATGAATATTTAGGGGT
AGACGTGTAATGCCTATCAACTCCTTAAAACCAATATTTGTTATCCCCTATTTTTTGTTAGGAGATCATCATCAC
TACATGAATCTTGTTTTTCCATCCATTTGTGGCTAACTTACTATTAGACATGCTAATAAAGTCACTCACCTCTTC
AAATAACTGATACTTAAAGAGGTAGGGAGAAGCGGATGAGACAACAGTCAAAACACTGGTCCACCACCCA
CTGGGGCTGAACTACTAGCGCTACTGCTTGCAAGTCATGGGGAATTAAGTGAGGGAAGGAGAAATGGAGA
ATGTGAATAGAGGAGAGTCTCGACTCTAGATTTGGAAGTCGGAATTGCTATTGCTACTTGCAATTTCAAATT

CGTCGGACAGTGCTTGCTGAGTTTAGAGGGTTGATTCATATTTCAAATTTGCTGATTTGGCCATTTAGGAGTT
TCATTATCATTGTCAAAGAGGAAGAAAGGGATTATGGGAAGGAAAGGGTTTTGCCACTTTTTGGGTTTTGG
CTTTTGGAATTTAGATTGAATTGGGGGAAATGTCGACGTCTGGAAACTAAAAGACAAAAGTCCAAAATCTA
AGACTAAGAGTCATGTGCCAAGCCATCACTCATCACCTATAACATTTAAAGGGTAATGATGTAATTTACCATTG
GGTGAGGCTGGTTTAGGGATTTATATGTAGACCCCATCCCTAGTTCAAGGTAAAAAAATCCTTGAACCTGAC
CCATCCCCATTTAAGGTCGAGGAAAATCCACCCCGTTCTGGGCACATTGGATCGGGGCCTCAATTTAGTGGG
GAAAAGTGTCATCCCTAGTTTTAAATTGTTGCCATTTATGGAGAAGACAAATAATTATTGTCGTTGTTGGGGA
GGAAAACAAGTTAAAATCAATAATTTTAGGAAATCAATTGAACCATGACCCAAACTTATTTCCAACTCAGTTG
TACCCTATAAATTGGATTGCATAGAGAACTGCTTAATACCAGATGACCCATCAGTTTAACCAAAAAATCGATG
AATTGATCTTAGTTTCTTAAAACAACTCGAGTCTTAAATATATCAAATTTTCAAAATGCAAGTAATAAGATTTG
AACTAGACCTGTTCATAGACCGGGAAGCCCGACAGGCTATCCCAGCTCTACCTGTTATTAAGTCGAGCTTGG
TCATAATTTTAGGCCTGTAGATTGGGCCCAACTTTTTTTTGGGTCGAGCGGCCTAGGCCTGAGCTCGACTTG
ATACTATATACTAAATGATAAAAATAAAATATATATATATATATATATATATATGTATATATATAATATTTTTTTCTTTG
TTCTTCTTCTCTTTTTTCCAAGTAATAGTGAGATGTTTATTATTTTTGAGTTTTGAAGGTTTTAATATAAGTAA
AACACTTTTTTATTGAATAAAAAAGAAAAAAAATTCTATTTTCAAATTTCTGGTTGGGCTCGGGCCCGGGCT
TGGGCCCGAGTGTATTCTTTTGATACATATACAAAGCCCAGCCCGACCTGACCAATGAACAGGTCTAATTTAA
ACCCATGACCTTCATTCATGTATATTGACTTTTTACCATTAATCCCCTTCATTGTTTACGTTCACTTATAAATATTT
AATGCATGTACTACTTTAATAAATATGTATTTTAAAGAAGATGGAAGTTATGAGGTTTGATCCCTAGTCAATTG
CGCTAAAACTTAAAGAACTAACCATCTATCCACCACATTTAGGTGCAATAATACCTTCGCTTTATAGTTAAACC
TCTAATCCATGAACCTCTCCCTTTTTTGGTTATATGTTCAATTCAATTCTTTGAACACTCATTTTGTTTTTAAAAC
AAATGTCGCAATCACACTCACTTTTGTATGTTGTGGTGGTAGAATAGTGGACTACATAGGATAATGATTTGGC
CAAGGAATTGCCTGCATTTAATCCTAATACAACATGCTTGAGATTGTTGGGTCTTGAGAAGCTTAAATGAAAA
TAGGGAAGAACTTTTAAGTTTTAAAAGTTTTAAAGAAAAGAGTAAAATGGTCTTAAAAATAATGAAAAAGAT
AGTTATAAAGTTTTAAGTAGAGGTGTAAATGGATTGAATTTGAATCGAATTAGGCAATATCCGAATTCGAATC
CGATTAGAAAATTCATTATCCAAATTTGGTTTGGATTCAACCAATTAACTATTTTAGGTAATTAATAAATATATA
AAATAAATATTTTATTTATATAATTTAACTATTATATATATATATATGTTATATAAAATATTTAAATATTTTATATATCAT
ATAAAAGAATTAAATAATCGACCGAATTCAGATATTTATCGAGTCGAATCAGGTTAGATCCGAATTCAAATAC
GATTAACTATTCGGTATGGATAAGGTATCCGAATTTGATCGATTATATATTTAAATTATCCAAATTCGATTTGATT
ATGTTGAATCTCGAATTTGAAATTAGATTTTTGAATACCATTGACACCCCTAGTTTTAATATGCAATTTACTAAT
ATACATCTTGCCATGATTTTCCATGCAAAATGACATTCTAAGGGCTTGTTCATTTGCTCTAAAACCTGACATCT
GCAAGTATAATAATTACATTAAGAAGTATATTGGCATTGATGAACTTTTCTTTTCTCTAAACTCTGAATGCAGA
GAATATTGTTCTGAATTTTGAAAATTATCAATTATCTATTTTGACCCCATATATGAGAGTTTTTATTTATCTTTTTT
AGAGTATGGTTGAAAAATAAAAAATTGTATGATTGAACTTAAAAAAATAAGAGATAAAAAATCGGACAACCA
TTTTTCTCTTAAAATTTAAAGATTTCTATTAAGATATAAAATTTAAAAAGGAACGAACAGGCACCAACTTCTAA
CTCAATAATCTCAAACACATGTTTCACTTTCGTCGCTGGTTTTGGCGTTTATTCTTGAAAATGCAATTGTTACC
TACCTATCAAAAACCAAAGCCGCACATAATTAAGATGCTAGACACCCTACACACCTTGCTATTAACTATTACCT
ATTAGCTAGGAAGAGATAGGTAAGCACATGCGAAGCACGAAGTAGTTGGAATATGTGGAGAGGGAGGGG
GAAGGGCTGTTGAACAGTTGGAGTTGGAAGTTGTAAGTGTTGAACGCACGATGACGGCTGCGACAACCAC
CGCTACTCTCACGTGCGAACCCAATCTCCCACTCTCAACGACCAAACCGTGGTGGAAGGTAGAGAACCAAA
CGCTCAGATCTCCCCACGACACACCCTCCCTTCAACCCATCCTCCTTCCTTTCGTGGCATTTCCTCTCACCGCA
CACACACACACACTTTCTCTCATTCTCTATTAAACCCGTCTCTTCTTCTACCTCTTTACCCAGTTCAATCCCTCA
ATTTCCGGTCCCCCGCTCTCTCTCTCTCTGTCTCGAACCTCTTGCACGCGTCACCATGGTTGCAGGGGCCGA
GATAGTGCAGAATGGCTCGCACTCGCAGATCAAAGTCCTTCCATGCAGCATGTGCGAGGAGGATCCTTTGA
ACTGGGCCAAGGTGGCCAAGGAGCTCCAAGGTTCGCACTATGAAGAAGTGAAGTGCATGATCGATCGATTC

AGTCGAACCAACTCAGTCAACTTGCAAGGTCAGAACCTCCAAGTCGCAGACGTCGTCGCCGTCGCTCGTCG
CCAAGCCGACGTCGAAGTCCGACTCGACGCCGACACTGCCAAGTTCCGGGTGCAAGAGAGCGCTGCCTGG
GTCTCGGCTCAGTCCTGCAAAGGTACCGACACTTATGGCGTCACAACCGGTTTCGGCGCCACCTCGCACCG
CCGGACGAACCAAGGGGTTGATCTTCAGCGGGAGCTTATCAGATTCTTAAACGCCGGCGTCATTGCCGGAG
ACGGAAATGAGCTCCCCGGCGATGTTGCCCGAGCAGCCATGCTTGTACGTACCAACACTCTTCTTCAAGGCT
ACTCGGGCATCAGATGGGACATACTTAGTACCGTCAAGGACCTCCTCAACGCTGGCCTGACACCGTTACTCC
CCCTCCGCGGCACAATCACAGCCTCCGGCGACCTCGTCCCGTTGTCCTACATTGCAGGAGTAATCACCGGGC
GTCCCAACTCCAAGGTCCGTACATGCACAGGCGAGCTGATCTCCGGAGCGGAAGCTCTCCGGCGCGTGGG
AGTGGAGAAGCCATTCGAGTTACAACCAAAGGAGGGGTTAGCCATAGTCAACGGAACCGCAGTGGGAGCA
GCGCTGGGGGCTATCGTGTGTTACGACGCTAACGTCCTGGCGGTGGCGTCTGAGATCGCATCGGCGATGTT
CTGCGAAGTGATGCTGGGGAAGCCGGAGTTCACGGATCCGTTGACTCACCGGCTGAAGCACCATCCGGGT
CAGATGGAGGCGGCGGCAATGATGGAGTACGTTCTCGCCGGAAGCGGCTTAGTTAAGAATGCGGCAAAGC
TTCACGAATACAATCCCTTGCAGAAGCCGAAACAAGACCGATATGCTCTTCGTACTTCTCCCCAGTGGCTGG
GCCCACAGGTATGTTCACTCTATACTTAATCTCTCGATCGGACGGTTGAAAGAGGTCGGCTGTCGATCGATAC
ATGCACATGGTGCACGGATGTGTAGGTTGTAGAAACGTTTTCGAGCCGCCGTTGACCCGGACGGTCGGACC
GTTCGGCCCGAGAACCGTCCAGGCAATCGGTTCTATTTACCCAAAACCCGAAATCTGGTCAAAAATCATTG
AAAAAGTCAATAAACCGTTGACTTGGGTGGTCAAACCATTGATCTTTCAACGGTTTTGGACCATTTTTTAAT
TACTTTTAATTTTGCTGTTAGAATAGGTCATCTGTTCGAACTGGACGATCAGACCATTGACCCTGAAATCGGT
GTTCAAAATGGTTCCGTTTCCGAGTCATTTTTAAAAATGTTGGGTTGTAGTTATATCAGATAATCATACGACGA
ACGTGACCATAAATGAAAGAGGGGCCCGCTTAAGATTATGGAAAGCTGGAGAAATCAATATTTTAAAAACTA
ACCCGGAACCAGATTTTGAACAAATACCATGTTTGAGGTAAAATAGAATCAGATGCTTAGACGATTTTCGGT
CCGAATGATCTGACTATCTGGATCAACAGCGGTTCGAAAACATTGGGCGAGATGCTTAAGGTTAATACATATT
TATTACATTAATACATACTATCAAAGTCAATCTATTTAATTAAACTTGAAAAGTTATATAATAAATAGGAGCTAAA
ATTCGGATAATTTGCTCGTGGCTAATCAAATCAATTGCACTTATTATCCATGTGGTGGCAGTTGAATAATTGAC
TTATTATCCATAATTATAATTTCTTAAACAAATTAAAATTCTTTTAAAAAACAAAAATTAGTAAAAGAAATGAAA
ATATTAGATAGAAAATAATAAAAAACGTTTATAAAAGTAAAAATTGTAAAAGATAAAAAAGAATAACAAAAAA
AACTTCACTCCTCTCTAGATTTGTAGCATGATGAAAATTGTTATATTTTTTTAGTATTAATCACTACTTGACACCC
CTTACGGGTCATTACTCTTTTAGCTAAACAATACAACATTACATTAATATCTCTTTTCTTTGATAATAGTGGGGA
TTCAAAGTCCCACCATCGATGCGACAATTTATCTACCGCACCTAATGTTTTTGAACCGTTGTTGATTTAGATGA
TTGGACTATTTAACCTAGGAATCGTTCAAGTAACTAGTTTTGTTTTACTCCAAACCCAGTTCCAGGCCAAAAA
TCGTTGAACTGGTTAATTTATTGAGTTAAGTGGTCGAACCACTGACTCGTTTGACCCATTCAATGGTTTATGG
TCATTTTTTATTTTAATTTATTTAATTTTTTGTTAAAATGGGCTATCTATTCGAACCATTGACCCAAAAACCAATG
GCCAAATCAATTCTAGTTCTAAGTCGATTTCAGCTGATAATGAAAATTGTTATATGAGCTCATGGATTAGAGTT
GTCCAAGAATTTGGAAAGTCCAATATGATTTCAAATCCAATCTCTTTACGAGTTACAACCCTAAATTGAGTGTT
AGATATTGATAATTGAATTGGACTTAGATACTAAGGTGACTCCTATCCAAATCAGCCCTAACTAGGGGTGGAT
TTGAGCCGAGCCAAGCCGAATACTTGGCTATTCGGGCTCGACTCGCTGAAATTGTATTAGGCTCGAGCTCGA
TTCGAGCTAGAGACTTGAGCTCAAGCTCGACTCGGTTCAAAGCTCGCAAGTATGAGCTCGACTACAAGCCA
AGTTTTTTTTTTAAAACTCGTCTCTTGTTTAATTTGTAAGCCTAGACAGGTTAATGATCCGTACTTGGCGAAGC
AGAATTGATGTAGCCGATTACTTTGAGTTGGGATAAGGCTTACAAGATGATGTTTGTTCTGAGCCCAAAACT
GTGCAATCGATTATTACCATTCCAATGGGTTTAATGGCAAATGGGAAAAATGTTTCGTTGTAAAGAAACCGG
GTTCAGTCGGTGCAAAGCCCGTTCTAACACTTATTATAGGCTTGCATTGAAAATGTATATTTATGGGAAGAATA
ATACAATAATTTTATAAATTAAAGAAAAGATAAATTAAAAAAAAAATATTATATATAAGATATTATTATATAAATA
ATAAAAATTATTATTATATATATACATATATATTTATATGTATATGTATGCATATAATAAAAAATATTTATTTATTATAG
TAGGCTCGATTAGGTTCGCGAGCCTCACGAGCCGAGCTTCACTGTGCTCGAGCTCAGCTCGATTAGCAAATA

AGCCTATATATCAGGCTCGAACTCGGCTCGAAGCTTGGTTTAAACCGAGCTAAGCCGAACTCAAATAGCTCG
TGAGAAGCTCAGTTTGTGGCCGCCCCTAGACCAAACCCTAGGTTTAGAGCATGGTTTTGAAAATTAGACCG
AAAATGGAACCACTTTAACCATTGATTCTTGGGTCAAATGGTTCGACCATCTGATCAGAACCTTGGTCGGAT
GGTTTTGGGGTCTAAAGCTGAATCCGGAGCCATGCCCCTTCTCAAGTCAAATGGTCCGACCATCCATTCCGG
TCCGACTTTCCAAAGCATGGTTTAGAGTTTGTAACAAGAGATATAGTGGGTCAGACTCAGAGGTAATCGATG
GGACAAGCCGGGCAAGGAAAGGTCAATCTGTTTGCAACGGGTTGGTGGGTAGGGTAGAGTCAAATATTGT
GAACCAAAGTAACGTTGATTACGTTGGATGCAGATCGAGGTGATTAGAGTGGCCACCCACATGATTCAACGT
GAAATAAACTCAGTGAATGACAACCCGGTTATTGATGTGGCCCGAGACAAAGCCCTCCACGGTGGCAACTT
CCAGGGGACCCCAGTCGGTGTAGCGATGGACAATCTACGTCTAGCCGTGGCGGCGATCGGAAAGCTGATGT
TCGCGCAATTCTCTGAGCTAGTGAACGACTACTACAACGGAGGCCTGCCTTCCAACCTCAGCGGCGGACCC
GACCCCAGCCTGGACTACGGATTCAAGGGTGCTGAGATTGCCATGGCATCATACACGTCAGAGCTTCAGTAT
TTGGCAAACCCAGTCACAACCCATGTACAGAGTGCCGAGCAACACAACCAGGATGTTAACTCTCTCGGCTT
GGTATCCGCCCGGAAGTCGGCGGAGGCTATCCACATCCTCAAGTTGATGACTGCAACCTACCTGGCCGCGCT
CTGCCAAGCCATTGATCTCCGCCATCTTGAGGAGAATCTCCGCCAGACCGTCAAATCCGTTGTTGCACAGGT
GTGTATTCTAGTCAGCCACTGACATAGCTACACTAACAGTTATTTTTTATTTGACTTTTCTTTTTTAAGAGTATAT
ACAATTTAATTCATTAAAATATGGGCCGCTGTTTCTGGGGTTCAACAACCTTGGGCCTAGTTTTTCTTTTTTTG
TTCAGGTCCAGCATAGGCCCACAAATTTATGGCCGCGCCTGGGCCTAATACTCCAGGCCCAACCTGGCCCAT
GTTATAAAATATTTATATATGATTTTTTATGTTGCATTATATAGTCTTATATATATAATCTGTGATATGATATGCATAA
TTCATATTTTATATATTTGATGTATTTATAGATAAAAACACGGGCCCGCAGCTTACTGTTTCAAGGCCTAAAACC
CACACTGGGCTGGTCTGGGGCCCAGGCTATAGCCTTAACCAAAGGTACAAAGCCTGGCCTATGATCACCCCT
GGCTTTTTCTAAATCAATTGGGTTGGTCCAAAGTTCAAAATATTGTTATAGCCGATATGCATTGTATCGAGGCC
TTCTCTATGAGACCAAATCAGTCCCCCCACAACATCCCATAGCACATTTTCACCGTGTATTGAAAACATCTCTC
AATGATAACCTCAGAAATGATGATGTCATCATTAGGAGATGCTCTCAATATACAACAGAAACGTGTTGTGGGG
AGCAATCTTGTGGGGGGCTGATTTGAACTCCTCCTCTATATGCCTCTATAGGGGATGTATCGGGTCAGGCCAT
GGTTTAGAGGATAGTCAGGACTTAAACCAACCCTTTTGAATCGGTTTTGAAAAAAAATAAATAAAACACAAA
AAAAGCACTGATACAAGCTGATACACCTTTAACGGCGTATCGGCCTGTATTGATATTTAGAACCTTGGGTTGG
TCTAATGAGTAAGATATGGAAAAATCTATGTTGTCGAGAAGAGAATTCTAAAAGAGTTTTGGCCAATCAACA
CCACCATACTGCCTGATTAGTTACCAATTCCTGGTTGATTGGTTGCAAGCTTTTCTACTATCATGTCTTTTTGGT
ATCATAGTTGAAATCTTGCAATTTTTCAATTTTTTTTTTCCTTTTTGTGGCAAGGTCCATACCCAGTCTGGATCT
CTTAGTGGCAAATCCTTCCCAAATTCCCTACCCATAAAACACCACCCCTGTGTATTGAGAATAATCTGATATTCC
CAACAATGTCGTGTGTGTAAAATTGTGGGAACAGCTTGTGGGTATCAGATCCAAGCGCGTCCCATGCAAAAC
ACTCACAAAATTGTGGTATGATTTTTTTGAGAAAAAAAGTGCAGCCGCTCAATTCAATTAATTTCTCATGTAC
CATACCAAACGCCAACTAATTGGGCTTATATTATTATATGTATCTTAGCAACTAAAACTCATTGATATTCCATGGC
ACAGGTAGCAAAGAAGACCCTAAGCACAGGACCCAACGGTGAGCCGCTCCCTGGCCGATTTGTTGAGAAA
GACCTGCTCCAAGTAGTGGAGAGCGAACCAGTATTTGCCTATGTGGACGACCCTTGCCGTGTCGACTACCCT
CTCATGCAGAAGCTCCGGCATGTCCTCGTTGAACACTCACTACAGAGCTCGCATACAGAGGCGGAGCTGTC
GCCCAAATCTGGTGTCTTCGGACGGATAAAGATGTTCGAATCCGAGCTCAAAGCGCAGCTCAATGCCCAAG
TTAAAATTGCGCGTGCCAAATATGATAACGGAACCCCACAGGTTCCCAACAGGATCGCCGATTGCCGGTCGT
ATCCGGTCTACAAGTTTGTCCGTACAGAGCTTGGTACTCAGCTGCTTAGTGGCACCAGAAAAGTGTCTCCTG
GGGAACAAATCGAGGCCGTCCATGCGGCTATCTGCGATGGCAAACTGGTTGCTCCATTGATGGAGTGCTTG
AATGGATGGCCCCAGAGGCCTGGGCCATTTTAATTTGCTTTACTTTGGTGTTGCCTTCTTGTGTTTTACTTTTT
CTTTCTCCTCCTCTACGGTTTGTAGGAAGAGGCGATTCCAAATAAATTATAGATGTTGCAAAAAGGAATAGAT
GTGTGAAAATATAAGCCTTTTCAAGTTGCTTTAATGATTTTGCTCATAACTCAAATGGCCAGCTACACATTTTG
TACAGAGAGGAGGAACCTATGTGGAAGACCAGATTGGATTATTCATTCTTGCCACGTGGCAGCATCTTATGT

TTGTTTAATTGTTTAAGAAAATTATTTAATTTGTTGTTGTATACAATATAGCAAGTGTTTTGAGGGTGCAATTC
GTGTCCTGCCTATGGGCTACCCAACTGGGACCAAACCCAATTTAATGCGGTTCAATATTTTCAACCCATTATT
GACCCGACCTGTCGGACCAATGGCCTGTTCACAATAAAAATTTGGGTCAACTTCCAACTTGGGATGGCAATT
TTTACCTACTATTGTACCTATCCAGCTACCCTTATTGATGGAAGGCATTAACCCGTACAGATATGGGTATAGCAC
GGAATTAGGTTCAACTCAATCCTCCCCAACCCGCATATGATGGAAAAAATATAAATTTTATGTATTAGGTGTTG
AATTTAATAATATTTTGAATTTTATATAAAGGATTGATAGTTTATTTGTTTTTATATGTCTTACTTAAATAATTGAA
AATTTTGTTGTTTGTTTGTTTTATTTTACTTATTTATTACTTATATATAACAAAAAATAATAGGAACCCAATCCAC
CCTACCCTTGAATGCAGGGAGGGCAGGGTTGGGGTCTAGAATTTACAAAAACCCACCCCGTTGTCATCCCT
ACTTCCACCCCCCTATTTTACAAAACTAGTTGACCATTGACCACCTAACCCATTCAGTGGGTTACTGGGTTTAT
ATCTTGTGCTTAATGTGTGATCAATGGGCTTATATTTTATTGGGCAAAATTTGAACCCTATGATGTAGGGCAGA
GTGATAGGGACCTGGGGGAGCCATGGCCCCCCTATTTCCCAAAAATCCCCATTAATATTTCCCCTCTCCCACC
CCACCCCAATGGTTGCCCTCCCTACTAATGGCCCATCCATGTCTATTCTCCCCTCTCTCCTCTCCATTTTAGTTT
TTGGCCCCTCCAAAAAAACTTTTGACGCTTCGAACATCCAAAAGTCACCAATGAGTTAGATTGCTTGGCACA
TACCTTGGACATGGATGATCCCTAAAACCACAATGGAGATTTAGAGCCCCCAAGGTGATCTCCTAGGGTGAG
GACTCTGATGATCAAGACAAATGAGTCTGAAGAGAGAAAAGGGAGTGAGTACAAGGTGTGTGAGGTGAGT
ATATTCGAATTGACCTTTGAGAGATGTGACTGAGTCATCAGATTGGATTAGATATATCAGATTGGATTTGGATA
TACCTTGATTCGAATGTGATCCATTTGCATCTCCAGGTTATCTTACAGCTAGAGCATGGGTTAATTTCTTCTAA
AGATGAGTGGTCTTCAGTTGATCCACAAGCATCTTCTTTCCCAAGGCTAATGCACATACTTGGGTGGTAGGA
TTGAGGGTTCTTGATGGTTTGGAGTATCCTACAACTAGAGCATGGGTTAATTCCTCGGCTGGCTACTTTATGG
TATGAGATTGGAAGCTTCAGTTTATTTTTGTGGCATGTTAAGGATAGATTTAGGATAAAAGAAAATTTTGGAT
TATTGGGGTGTTGATTCCCTACAAATGTTGCTTGAATGGCTTGGGTGGTGATTATATTGTTTCTTCTTGATGGC
CTCCCATTCCGCCCTGGATGTAATGGTTGAGGTTGCAGTAGTGGAAGCCTTCTCTATTGTTGGCATGGCGCTT
ACTCCAAGTGTGTTTTCTTCCTTGTAAATATGGAGGGCCAAAACTTGCTTTTTTGTGTACCTTGATCACGGCC
ATCATTAGGATCATGCAAGGTGAATGCTTGTCCTGGATGAATAGACTGTTGATGTGTGGTATAAAGGTCACTT
TTGCTTGTATCAAGAAATATATTCAATGGCTCACACCAAGGCAACCTAAAAATCGTCTAGGGATATAGCAGAC
AAGTTAATCTTCCCCTTCTAGGGCCCGACTTGGAGGTCTACTACATGGGCTGTGCCTTCTATCACAGTGGCAC
TAAAGTTGACAACTTTGAACCACCTCGATTCCTTGGAGATGGTCAATTTAAGTTGCATTGCTTCAACAAGTAA
CATTAAGTTATGTGTGGCCCTTGTGTCTCGTAGGGCCTAAGTAGCTTCTTGTTGAGGGTGATTTTCACATATTT
CAAACTTTTATGAGTTTTGTTGGGCCTTTAGGTGTGGCCTCTTCCTTTACCATAGTGTTGAGAAGTTGCAAGA
TGCCCATTCTTGGTGCCTATGTGGGTTTCTCCTTATTGCTTTCATCACTAAGTTTGCTAGTAAGGGCATTGATT
TTATTCCTTGTGGGACAACCACTTGCCCAATGAATTTCATTATAAACGAAGCAAGGTGAATGAGGAATTGTG
GATCTCCTAGTGTACTTGTAGTTTTGGGAGTTATAGGATCCTTATGGCTTCTTTCCTTCTTAGTGATTGTCAAA
CTTGTTGGGCCAGGGCAGTTTTCTTGAGTGGATTTGGTTGTCAATCACACACACTCAAGAGTGGTATGTGGT
CTGGTTGGCTTGTGATCTTCTACATTAGGATTCCATAGATCAACAAATTGCTCGATGCCACAACCATGGTTGTT
GCGTGGTCCTTTACCCTTCACCATTATAATTCAACCTCAGCCTAGGGATGGAGACCGTTCATGAAGTTGAATA
AGAGAGATCCTTGTTAGACATGTCAGTGATCTCTCGGGGCTAAAAATTTTATGACATACTCATGTAATGAGTC
TATATGTCTAAGGCTCTTCATCTTCCTTCGAGCAAGGCACTCAACATTTTCAAGGTAAAATTGTCACTTAATCT
CATGTTTAAAATCAACCTAGGTGTTGAGGGTGTGTGTACCATGCTCAATGAGAGTACTAATGGTTTGTACTAA
GTGTGCAACTATTTCCATTGAGCTAAAAATGATAAACAAATGCTAGATGAGATAAAGCATGAATGAAAGCATT
GGGTAGCTATTAATTAGAGGTGTAAAGTGGGCTATGCCGAGCTCGCACGGCACGGCCCGTGGGCAGAGTG
CGGGTTGTGCCATGCTGGCCCATAGGACATCACGGGTCGTGCTGCCCTCCCCGATGAAAATTTTGGGGGTC
AACCCAACACGGCCCACGACCCGGGTCCATGTGGGCCCATGTCATGCCACGGGTTGGGCTGGCCCTTGCGC
CGAGCCTTGGGCCAGGTCGGGCCGTGTATTAGGCCAAAGCTATTTATTTAAAAAAATTGACAAAAAACTTAA
AATTTTAATTTTATATGAATTTTGTGTATAAAATTGCTTATATATGAAATAATTAAATAAATTAGTATAATAAAATT

AAAAATTATATAATTATAATATATATAATGTATATAAATATATAGTATATATATATGGGTCGTGCTGGGCTAGGCGG
CATGGCCCGCTCGATCGTGCCATGTCGTGCCGGCCCATGGACCTAAAATTCCAAGCCCAGCACGGCATGTAG
ATCGTGTCAGGCCTAGCACGGCCCATAGACCATCATGCCAGACTGTACCGGGCCGGTCCAAGTTCATATCGG
GCCACGGGCGGCACGGCCCAATTTACACCTCTACTATTAATCATGATGAATCTCTCCAAAATGATATTAACTAC
CATGAGTTAGAAGAAAGAAGAAGAAGAATATCATTCAAATCCAAAGAAGTTCATATTGTGTGGTCAAAGAC
CAATCTCAATTAACATGAACCTTAGAGACTAATAGTTACATTAAGCTTGGTTTTTAGGTGTTCCTAGGCTTTAA
GTTTGTTTAATCTTGCTTTGAAAACCTAAGGCAATAATATCTATTCATTTAGAAAAAAAGCATGCAAGGTTCTA
TAAGGTTCTAAATAGTTAGGAGTTGATTAATTACTATTAATGAGTCACCTACCCTTGACAATCGAGGCATGAA
GTATATATTTCACTATTTATGAGTCCTTATGATGAGTCGACTACCTTTGACATCTGAGGCATAAAATGCATTGTC
ATTGTTCATGAGTTGACTACAACATTCTGATAGTCCATTACCTTTGACGATAACCAAGAGTTGGCATAAGTTG
TTAGTCAATTAATATGAGGGGCTCATCAACTATCAATGAAAATAGATTCCCTCCTGTGAGTAATAGTCAATCAA
TAGAAGTCATCAATCCACTACTTGTAGTCATAAATGATTGTTTGAAGGTTCAAAGACTAGTTTCTTCTTTTAGT
AAGCCTTTTATTCATTGAGAAATTGTTCTGAAGATTAGTGTGATCCTCTCCAATGTCTCTCTAATGGTCACTTA
GTCTCAACATCTATAAAATGATGCTTAAGAACTGAGATTAGACAAGAACTACAAAAGAGAAACATCTACCATA
TTAAGAGCATTTTCTATGAGCAAGAATCTTAACAGAAGTATAGTTCTCTGAGCTTAAAACAATTATGTAAGAT
AAACTGATTAGGAAGTCTGGTTTATAGGTATGGGTGAGCAAACAATTTGGTCAATCCGATTAACCAAATTTCC
AAACCTAAAAATCAGAACCAAAACTAAAATTCTATAAAATCAAAACAACCAAACTAAAGTAACCAAAAACTG
AAATAATTAAACTGGAAACACCCAAACCAAAACTTTTGATTCAGTTTTTTTTTTTATTTTTTTTTATTTTAACC
GAACAATGCTTACCCCTACATATATGTTTCTCATAGAGATTTGAGAACTCTTCATGTGTTAAGCTTGTTATTATG
TTGTATCATCTTGCTATAAATATTAGTTCGATATGGATATGCTTATATCTTCCCAAATAAAGACTTTTACTTGTATT
TGTACTTATAGTGCCTTATCATTTGTATCTGAGGTGTTCCAAGTGTTGAACAACAATGTTGGTAGGAATAACAT
CAAGAAAGTCTATCTAGCTTTAACATATACTTGGAAGTCCTAGGTTGTAAATGTTTGATTGTCACTTGAGAAG
CTCAATTGATTTGAGTTAGTGAAAATCTCAAGAGGGTAACATAGGCCAATTTTGGCCAAACCTCTATAAAAAT
TCTTTGTGTGCTTTACATTTTCAGCAATCTTTATTTTCTTTTTTATTATTATTTGGTTGATTGTATTTAATTCCATT
ACATCCCCTAGAACATACAATCTTCACACTTATTATTATTGCATTCAATTCTTATAACAGGCTTTAGGCAAGAAA
AAGGGAAAAAGCTTTTAAACTCTCAATTCACCCCCAATGGATTTGCTACCTGGGGCAACATTAGCAACACCT
ACACCACCACCACATTGCAATATCAATTAAATACATAGCGGACGTACTTAATATCATCATTGATGCCTATGGCA
GAGAAATATTTTGAGTTACCATAAGAAGTTGTTAAGCTCCCAAGAATCTTAAGCACTCGAGTATAATGTTGGT
TGTTACATCTCAAGCACCCAAGTATAATGTTGGTTGGATTCTAGTGGAAGTTGACATGACTAGTTTCATGGCC
TTCCATTCCCTTTTCATCTCATCCAATTAAGCATGGAATTCATCCATGATGCCAAATGATAGTTTAGGACGATCC
AACATCTAAATGTTACAAGGCTTCATAGATCTACCTAAGAGCTTCATCTAAGCGGCCTTCCAACTCCAAAACT
GAATTTACAACAGTCTTTAAGTTGAGTTAGAGTTCTTCAACCCAATCATGTGTCTCACCCATTGTTACTTTGCA
ATATAGCATTCAGGTGTCAGTGTTTGCAAGCTTGTTGATGGATTTCTCACACTTGCTTGTTTTGAGTGTTCAA
ACCTTTAGGGCTTTGTCTTTGCTAATATCATCTTTGGGTGCATCGCCACTTCGTGGGTTTGGGAGAGGCCTT
GTTGTATCTCTTCCACAATGTCCTAGTTGCTCTTCATTATGTTACTACCATTGAGGGTTAATCAAGTGGATCAA
AGTGTGTTGATGAGCTCCTGTTTTGATACATTCATGGCTCTAGTTCATGAGGCCTTATGCAAAATGTGTCAAG
TAAGCATACAAAAAAAAAAAAAGAAATACACACAAGAAAAAGCAATAATTATGGAACATGAATTAATAAAAA
GACACTTTGTATTGCATTGATATGTTAAGTATAAACACACAATTAATGTGTCAGGAGTTCCTTGTGCTTAAACT
TTACACCTTAATTCCCTTTTGACTACTTACATATTGATCCTATGTGTTTTGGTGATAGTAATGTCATTGAGCGTA
CTAATGAAGGTGTTAAGTGTGCACTGTGTAGTTATTCTAAGTTAATCCTATTGAGATAAAATGTTTAACAAATC
ACTTTAAGGAATCAAAGCTTAATGAATGCATTAGGAAGTTAACTTGTAAATCTCAAAATAAGAATGCTACAAA
GATCTAAAAGGAAAGAGAATTAAGTTCCATTGTTGTATAGTGTATTAGGTTTTTAGGACTTAATGTAAATCATT
TATATGTGATGTACTCTATAGGTTTAAACTTGACCTAACTCGATTGACCTAAGTATAGAGACCAATGGCTACTA
GTAGGTTTGGTTTTTAGGTGTTTTCAAACACATTTCTGGACTTGATAATTGAAAATCTAAGTTTGGAATCCTA

AAAAATGATGACTAGACCTTTGGAATAAATTTAATGTCTTTAAGTTTATGAATAATGAGTATTTAACTATCCATA
AACCCATAGTTAACTACTTGTGACAATCAAGACCAATATTAATGGGATGCTACTCGGTAGTCAACTATAAAAA
ATCAATAGTCAGTTATGTGTCACAACCGAGATTTGACATAAACTAGTAGTTGACTATTAGGAGGAAGTAGTCA
AGTGGTAGTTGACTATTTAGTCATGATAATCAACTACTTAAAGAAAATTTGGCCATTTGAAAGCATAACAACT
ATTTCCTCTTATAGTAAGCATTCATTCTTGTCGGGAACTTACTCTAAACATTTCCAAGTACAGTTTAATCTTTAC
CCAATGGATATATAATGATCATGTGCTCTTAACCACTATAAATTAGTGGTTTTGGATTGAAAAACTCAATGAAC
TCTAATCAATGAAAATTTGCTAAGATAAAGAAAACATTGTGAGCAAGAGTTCTTAGTGAAGAAGAGATCTCT
AAGCTTTATTAGAGCATATAGTGAGAAAAACTAAGTAGTTTTGGGCTTAGTTATTCCCCACTAAGATTTTTTG
AATCTCTTCAAGTGTTTGAGCCTATAGTTCTGTGCATACTTCTACATTTAATATAAACTTGTTTGTATTTGTTATT
ATCATCATGTTAAAAGCAATTTGCTATATTTATTCTTTACATTGCTATATCTATTTCATTTATGTGTTCAAATGTAA
ACATCGTTGTAGGCTTGATTAGCACCTAGAAGTCCTACTGGTTGTTAGATTTTGCTAGCACCTAGAAGTCTAA
CTATTTAGTGGAAATTCTCAAGAAATTGCTTGAAGATAAAATGTAGGGTGGGGTTTGGTCGAACCTCTATAA
ACCCTTGTGTGCTTTACATTTCTATAATTGTTTGCTTTACTTTTTATTTTACTTGTTTGTTTATTCTGTTATTTACA
TCGTATTAAATAACACACACACATACACAATATTCTATTCCATCAAAGTTGCATTCATTCATTTAGATGGTTTTA
GTTTGGGGAGAAAATCTTTCAGTACCTAATTCACCCCCGTCTTGGGTTAGGCTATTTAGGCAAAATCTGGTAT
TAGAGTAGCTTAACCATTAGAATATCTTAATAGCTAGGTGATTAGAATGGCCCCTCAAGATAACACCTCCTACA
CTAAAGGACAATCCATCTCTAGTCCTCCATTTTTCAATAAAGTATATTACAATAATTATTGGAAAGCAAGGAAA
AGAGTACTCTATCTCATGTCTTTAGATTATGACCTTTTGAAAGTAGTAGAGAGTAGACCATACAAGCCTACCA
AAAAGGTAGGAAAAGAGACCTCAAAATGGACTAAGGATGAGAAAAAGAATATTGCTTATATCATGCATTAG
ATAAAAATGAGCTTAATAAGGTCTCTACATGTGAAACATCTCACGATATTTGGAAAATTCTTGATGTTTCCTAT
GTGGAAACCAATTTGAATAAGGAGTTAAGAGCAAATCCACCTATACATACAAGTGAGTCAACCATGATGAAA
TCACAAGTGACAATTATTGATGAGTTCAATTGTTTTGAAAAAAAAAGAAAAAATTGAGTGACCATTGGGAA
CATGGAAAATCAATATGAATTCAAAAGGAGAAAAGATAGAAGCTATCAAGACCAAGTATAAATGTCCCCTTC
AAATGTTAGGGAGACATAAGGTAAGCTTGAATTCAACTCCACCAAGAAATTTTCATTCCAAGTAGATTTCTAA
AAAAAAATGAAACTTAGGAAAAAGATCAAAACCTTGTGATTTAGATTAGTTGTCTAAGGAGTCAAAACATCA
CTCTTAGCTCCTCAAGTTTAATCCTATTGGCCAAAAATGATGAACTAAGAAATGTGAATGAAAAGTTTAATGA
TTTATCCAAAGGTTAATCAATGAATGTATAAATTTAAAAATTGAGAAATGTGACCTCTTAGACACCATAGAGA
AGTTTACATGTGAGACTAATAACCTCATTAAAATGTTGGGAAGCCATAAGCCTACCAATGATAAGGGAGGAA
TCAGATTCAAAAGAGTCAATACTCCATAACTAGATTTGTAGGAGTAGGAAGTTTAGAACAAAACACACAAGT
GTGCTTGCTGTCATGAATAGGTCACTCAATTTATCAGTATTAATATAAAGAGAGTTCAAAGAACTCAAGTAAA
AATGTAATTTAAAAGAAGAAAACAACTAAATCTTGATGACCTAAAGCATATAGGTTATTAAACTCTTTCTTTTA
ATCTATGTAGGTGCTCAATAAGGAGTAAATGCTCAAAGAAGAAAGGATAATTAGTCAAACATAGATTTTAAAT
AAACAACACTTTATTTAGAATGAATGCTTTCTTATAAACTTGTTTGAATATCTTTTTAAGGACATCTTAATAATT
TCTATGATAACTCAATTAATTTGTGAAAAATATTAGTTAGTTCCATTGGGTACACTAGATTTCAAATTGAAGAG
TGTAGGCATTGTGGTGGGAATGGTCTAGGTTCTGAGAGTAAAAAGTATAAGGATGTGACGTATGGAAGAAA
AAGGACAGGGGTTAATTTGGTTGGTAGAGATAAGCTTCTTAGAGAACAAAGTGCTCTATAAGAATAATAGAA
TAAGGAAAACCCATCCCCTCCATTACAAGTCTTGCTATTTATAGACAAAGGAGGGGCTTTGGTTTAAGCAAG
ACTCCAGAATATTCTTTAGGGAGTTATAGGCTTAGGTCCCTAGATTAATTCAAAGGTGTTGACATGCGTCAAC
CTACAAAGTAAAGGGGACCATGGCTCAACCATCACTCTTGCATGGTCTTACTGGGTGTTGCATTAGGGGACA
AGTTCTGGAACCATGCAACCTGCCTTGTTTCTTTTTATAGTGGTTACTTTCTGTACCACCTTTTTCATTCTCATC
TCATTACTAGAGACCGCAAATTGCTTTGTTTTTTTGAAGGAAGTAAACTATTGTGTATTCTACCATAGAATCCT
CCACGGCTTCTCTAAATATGCAGTCACAGTTACTCGGTGGTGACATCATAGTATTATATCAGTCTATCTCCTTGC
AAAAATATACACAATCATGTCAAAAAGAGCTATGAAGATATCAATGTGAAATACAATTTTTTTTCACATGATTA
TTTCTTGTAAGGTTTAACCTCCAAACCTAATGTTGCGATCAATAATAATATTTGAAGTATGAGCATACTTGAGG

AGTTGACTACTTAAATCTAGCAGTTGATTATTAAAAACCATTTGCTTCAAGTAATCAACTATCTAAAATAACCA
GTCCACTACTAACACCCATTAGTTTACTAATTCATACAACCTTCCTCTTAAATACAAATCTTTCTGCAATGGTAA
AGTCTTACTTGCTCAAAACTAGAAAAGATAAGTTTGTGCTCATCATTTATCTCTATCTTACCTCATCAATGACCT
CATTAAGTGGAAGAAAAAGAGGAACGCTCCTACTAGTGCCCAAAGAGGAATGTTGGTGACTAAGGAAAGG
TACCAATAAGACTTTGCTGATAGAGAAATTCTTGAAGAACACATGATTGGTTTATCTTCTCTAAAGGGTGGG
GGATTAGATATTGAAACTCTTATAAACTACCTAGGGTGGGAAACCCTAGTTCAATTTCATAACCCAATTCTCTA
AAGCGCAGCGGTTGTACTTTAATTTTACCTTAAAAATTTTTGGACTTGAGACAAGTGTCAAAGGAGTAAGGA
TACGTGTTACACTAGAAGACTTGGAAACAATTCATGGGATAAGTAGGGAGAGACCCATGGTTTATGAGAAA
AGGAAGTGGCTTGATAATGTAGATGTTGAACATATAGATACACTACAAACTGTATTCAATGACCCCACTCTTG
ATCAAACTGATAGGCCTAAATTAAAGAACCTAAGAGTACCCATGAAGGTTATGCATATAGTAATGGCAAAAG
GGTTCATTCTAAATAAAGGCTCCTTTGAAAGTATTTCCTAGTTAGAATTTTTGCTGCTCCATTGCAATGATATT
GGAGATGACGCTCCTCTATTGCATCAAAACATGTATTCTCTAGTTAGAATTTTTGTCTCAAGTCTATTACCATAT
GGAATGGTGCTCATAGAAATTTTCCAACAAGAAAGAGTAAATCTTAACAATGAGACAAGGTCAAATTTATAA
ACATCCATACTTTGAGGAGCATGAACTTGGCATAAAAATGCTTTGGGGATTTAGAATGATATAGAAACTATTG
ACTTTTTGAATATAGAAGAGCCAAATGTGATGGGTGAACAAATTCCTCACTTTAAAGGACATGGGTAGTATG
GCCAGGATAACATGTCATCAGGGGTAAGACATTATAACCCTATAGATATTTCGTCCTCATTCAACATGAATGAT
CTTGCTAGTTGTCTCAACTCTCTCAACACTAGCATTTTTCTCATATGTGATAGAGTAATAGGGATGGATATATGC
TTTGATATTCTAGAAACTCACATAGATTCCATTTATGAAAATGATTTGATGACCATTCTCTCCATTGAAATGTTT
TATAATGAAATCTCTGAGCTCTAAACCCTCTAAGAAAGGATGAAAAAAAAGGAAAGGCAAGAATCCCATGC
CTTTCAAAGGAAAATCGTAGAGGGTGATTAAGGAACTCGATTGGAGTTTCGACAATTGTTAGGGAACAAGA
TCTTAGGTGTTGTTTAAATCATTACCAGTGTTCTCCATAAATTCTTTCTTTGTTTTAAGAACTAATTTATGAACT
CTTTAATATTTATATATTTATTAAGTTTCTAAATGTCTAAGAACTTATCTTGAGACCTTGTCATTATATCTATGAGC
TTAAACCAATTGATAAATGCTTTTATGTGTTTTTATGAGTTATCTATGTGCTTAGCTACTTATATTTTATTTTCTCC
TATTCCCCTTCTTTTTTGTTGATGCCAAAAGAGGGGGAAATATGTGCTTGATTGAAAAATGAAATACATTTGC
TTTTATCAAAAAGGGAAAATTTTAGAGGAAGTCTCTTCAAAAACTTTGAGAAAATTTGGAACAGACTCATTT
AGTCTACAACAAATCCATCAAAACAAGGGGAGATTTATCAATTATGTAGTAAACCCTTACATGATTCAAGGGG
ACTTTAGTGAAGGTGCTTGAATGTCTATTTTCCTTAGAAGGAGATCAATATATGTGCTTAAATGTTTGTATTTCT
AAATATTCACATGATTGTATGAATCCTATGCATTTATATTTAGAACACTGATAGGGGGAGTTATCATTGTACTCT
CCATTTGTGCTTTATTTGTTATCATTTTTCATCAATATTTTGTCATCATAAAAAAGGGGAAGAATGTTGGCACTA
TGTGTTTTGAAGATAATAAACTCATTGAGAGTACTAATGAATGTGCTAATTATACAACTGTTTTATGTTAATCCA
ATTAAATTAAAATGTTTGACAAATCACATTGAGGAATTAAAGTTTGAATGAATGCTTTAGATAGTTGTCAATTA
ACTTGTGACTCTCCAAAGAAGAAGATTGCTACAAAGAGCTAAAAGGAAAGAGCATCAAGTTCTAATGTTCT
ACAATGTATCATGTTTTTAGGATTTAATTTATATCATTTGCATGAGATGCTACACTATGGGCTTAACTTACTAAA
ATCAATTAACCTAACCTTAGAGACTAATGACTAGCAGCACGTTTGGTTCTTAGGTGTTACCAAACTTATTTTTA
AACTTAACAATTAAATATAAGTTTGGAAGCCTAAGAAAATGATTTCTAGACCTTTAGAATCAATATCTCAACGT
ATGGCTATAAATATCAATTATGCATAAACCTATAATTGACTGCCTCATAGCCAAGACTTGACATAAGCTAGACA
ATCAAGAGGAAGTAGTCGATTACTAGAGAAAAAAGATTCCTCCCTACGAGTGTAATTGAATACTATGTAGTGA
TAGTCAACTACTTGAAGAAAAAATTTGACCATTTAAAGGCATAATGACTATTTCCTCTTCTAATAAACTTTCAAC
TCTGTTGAGAACTTGCTCTAAACATTTCCAAATATAATCTAATCTTTTCTCAATGGATATATATAACGGTCATATG
CTCTCAACCATTATAAATTAGTGATTTGAGTTAAATAACTCAATGAACTTTAACTGGTGAAATTTTGCTAAGAT
AAAGAGAGCATTGTGAGCAAGACTTTTTAGTGAGGAAGAGATCTCTAAGCTTTATTAGATCATATAGTGAAA
AAAACTAAGGATCTTTGGGACTAATTGTTTCTCATTGAGATTTCTTTAATCTCTTCAATGTGTTTGTGCCTATA
GTTTTGTGCATACTCTTGTATCTAATATAGATTTGTTTGTATTTGTTATTATTAGCCTATTAAGAGACATTTGCTT
TGTATTTATGCTTTACATTTTTATATCTATTGTATTTGTATGTTCAAGTGTAAATACTATATTATAGGTTTGGTTGG

CACTGTAAACCCAACTATTCATGTTTGGTTAATACCCAAAAGTTCAACTATTCAGGTCTTATAAGAACCTAAAA
GTCTTACTGATTGTAAGGTTTTGTTGGAACTTGAAAGTCCAACTATTTAGTAGGAATTCTCAAAAGTTTGCAT
AAGGAGAGGACATATGCCTAAGTTTGGATGAACCTTTATAAATCCTTATGTACTTTACAGTTCTACAATTGTTT
ACTTACCTATTTTACTTAATCTAATTATTTTGTTATTTGCATTGCATCAAATAGCACATACTCATAGCCTTCTATTC
CATCAAGTTTGCATTCATTCATAGAAGGCTTTAATTAGGGGAAAATATTTTTGTATTGGGTTAGGTTATCTGTA
AGGGTGTCAATGGGTATGTTTTGGGTTGGACTCCGTCCAAATCAAAATCAATTTGGACAGACTGGATAGACC
AATAAGACATATTGAATTGGACCAGTCTAGACTTAATGATCTAAAGTATTTATTGGATCGGACTGGACCTGATT
CATCCATAAGAATTTATATTGGGTTACATTGTATTGAGCCAATATCTAGTCTATTGATAAAAGATAAGAATACAA
GTGTCAAAGGACTAGGTTCGAGTTGGAACCAACCTAAATTAAAACAAATTTGATCAAATTGGATGGTCCAAA
CATTGAATTGGATTGGTCCAAAATATTCATTGGATTGAATTGGACTAGGTCCATTTGTAATGGACTCACATTG
GACTGGATTGGATTGGACCAAATCCAATCCAATTGACACCCCTAGTTATCTGGGCAACGTTACCAAACCTTAA
AGAGGATGTTAAGCTTTTGGGGTTCTAACATAAAAATACATAAAAATGTTGAGGAGTCCTATTGATTATTACA
ATTATACCATTGACATTGCCTCGTACATAAATATAGACAATGTCCCATACAAGACCACATGGTGACATACTTATG
ACACTCTAATGGTGGAACTGATAAAATCAACCATGACATAAGTGCACAGATGTAATGAAGCTATCTATTTCTTA
ACACCTATGAAGAAGCCTTGTATCACTTTAATAGGTACCATCAAAACCAATCAACAAGCAAGAATCCAAGTCC
TTTTGTGAAGGAAGTTGGCATAGATACCAACATATCAATAGGAAAATCCTAGTCACAATGGATTATTTCCATAA
TTACAATAAACTACACAAACAAGGCACACAAGATTTATAAAGGTTCGGCCAACATTGCCTATGTCCTTTACCT
TAGGCCACCCCTAAGGATTTTCACTAACCAATTGGGATTCATAGGTGTCGGCAAAGCCTTTACAATTAGGAC
TTCTAGGTGCCTACAATATCTTAACCAACAAGACTTGTAGGTGCTTGCAATATCTAACCAATAAAAGTTGCAA
GTGCCTACAATATGTAACCAACAGGGCTTACGAGTGCTTGTAATACCTTGTAACTAAGCTTCACTTTGGTGGC
AATAAGACCCTAATGGTGTTAAAACTTAAAATCCTACCTGCAAACAAGCTATTACAAATATAGCATAAGACAG
GTTTACAACAAGAGACCTCTCAAAGGTAGATATAGCAATACAAGTACAAACAATTCAATTGGAAGAGTTTTC
TTAAATGCTCAACCCAGTGAAGAGATATACAATAAATGAGTGAGACTGTAAAAGCACAAAAGTTTTTCAACT
TTTTAGACCTATAAAACTATTACATCTTAAGAATCTCTCCAACAAAAATGATTCTTACTTTGTTTCTTTGAAAAA
TAGCTATAAACTATGTACAAGGAAGGCATATATTGTTTTTCTCTTGATTCCTCTTGCTTTGAATTAAACCAAACT
CCTCTATTTTTAGAAGATTTGAGAGCATATAACCATTTTCTAAATGTTAGAGGGATTTTGATTTAAATCCCCTC
CAATCTGATAAAAGGCTTTCGAAAGATGGCAATAGCAGTTTGAGCCTTCAAATGATCAAGTTCTTTGAAGTA
GTCGGTTGTCAAATTTTTGTTAGTCGGTTATTTCTCTTTTAGTCAATTGTTTCCTTTAATGAATTGATTGATATC
ACTTTAAACTTAGATTTTCTTGATTAGTCGACCATCTATCTCAAATAGGGCATTTTCATACATTGGCTATTACAA
TTAGTCAACTATGCCTTCTTGGGGTAGTCAATTATGTCCCATAGTTTTTGCAAGACATGCCTTGATTATTTAGTT
AGTCAATTATGCCTTTTGGCGTAGTTGACTATGTCTTATAGTCCTGGCAAGGCATGTCAGTCAGAGAGCCCAA
CACTTAGAAACATTGGAAATGATCATCCAAAGGTTTGGAACTTCTTCCTTTGGTTTTCAAATGGAATTTTAAG
ATAAACTCTTGTTTAAGTCTAGGAAAGCATAAAAACCAAACATATTATAATCATTGGTCTCTAAGGTTGCTTTA
CTTGAGTTTAGTCTTGTTGAGCATCAAAGAGAGTGCAAGTTAAAAAACTGATTATATAAAGATCCTTAAAGAT
AGACACATTTGAGAACAACTTAATGTAGTCCTCCTCCTTGATGAGTTGTTGTAGTAAATGGTCATTTAAGTAG
CTCCAAGACTTTCAACTCTTATAGTAGCCTTATCCTGCTTGAATGATGTTAGAACAACCTCATGTGGAGGATTT
AGTGATAGTGAAAATATAAAGTTTAGGCAACTAATCAAGTAGAACTTCCACTTCATCATCTTTGGTAGCATTCT
TTCTTTTACACAATTTAAGCTCAAAGAACCTTACTTAAAGAAGATGGGTAAACCGAACCGAGTTATCTATAAA
TATATTCTCACTGAAATATTATATATATAATATGTATATTTCAGCTTTAAATATATATATATATATGGGTCGGTTGAA
AAAAAACTAGTAAACCGACCCAACCCAACCCATGAACAGCCCTAGTGCCTATGTGGCAAAAAATGCCACCT
GTCATTGGATCTTGCTAGGTGCATATTTCTAAGACAATCCAAATTAGCACATTTACATGTTATATGGAATAAGT
CAATCATGAATTATTTTATGAATTTCTCAAATAATAACCTAACCAAGTCAGGTCGGTTGAAAAAAAACTAGTAA
ACCGACCCAACCCAACCCATGAACAGCCCTAGTGCCTATGTGGCAAAAAATGCCACCTGTCATTGGATCTTG
CTAGGTGCATATTTCTAAGACAATCCAAATTAGCACATTTACATGTTATATGGAATAAGTCAATCATGAATTATT

TTATGAATTTCTCAAATAATAACCTAACCAAGTCAGGTCGGTTGAAAAAAAACTAGTAAACCGACCCAACCC
AACCCATGAACAGCCCTAGTGCCTATGTGGCAAAAAATGCCACCTGTCATTGGATCTTGCTAGGTGCATATTT
CTAAGACAATCCAAATTAGCACATTTACATGTTATATGGAATAAGTCAATCATGAATTATTTTATGAATTTCTCA
AAATTTTCTTGGTAAATAAGCATCAAAAAATATATTTTCCAACATATATATTTAGCAAATGAACCAAACACATG
ACACCTAGTGGAGAAAATAAATTTCTCTTTCCCCTTACATCTATACTACTATTAAAAGTGGAAATGAAGTCTAG
ATTCCATAATTTTTCTTTACTAAAATACCCATAATTTGAATACTACTTATATTAAATTCACTCCATTCCCTCTTATT
AAGTCCTATATTTAAGGTATTAACTCCCATCCTTTCTCATTTATTTACTATATATTGAATTAACAAGTTTTGTGGA
CTTTTTCTTAATTAAAACACCCCTACTTTTGATTTTTGTCATTCCCTAACTGTGAAGGCAAAAATAATAATAATA
ATAATAATAATAATAATAATAATAATAATAATAATAATAATAATAAGAACTATTACTTTTCATTTCATTCAATTCCCT
CTCATTAAGGAGTTAACTCCCATTCTTTCTCCTCCATCCTTTATTTGAAGTATTTTTGTTTCTAACCAATAATTCT
ATTAATTAAATCTATACCCAATAATTTTAAAAATACAAAACCTATGTGTTTAAGCACAACATGAAAACTAAACTA
TACTTTTTGTTCGTGTGGTGGGTACGTCAACAAATGGTCTCATAGTTAAATAATAAATGTTTAAAAATTGATTA
AAATATAAAAGTTACAAAAACAACTTAGGCTCGTCTCTAAAATTATATATTTATCATATTAAACAAAAATATGTAT
AATAAAAAAGAAATATTTTTATTAATGATGAAAATGGAATATAAATTTTAAAACTGGCCCAAAAATGAAACTA
GTTTCCTATTCAATGATTTAAACTTTTAGGTCAAATTGGTCAATCTACATTGTCATAATTAAATTAAAAAAAAAT
TCAAAAAGATAGAGACATCTTTTTTTTTAAAATACAAAAAATTTGTAAAAACATTTGGGTACATCTCCAATAT
TATGTATTTCATCATATTAAACAACAATAAGTGTAATAAATAAGAATTATTTTTATTAATGATGGAAATGCAATAA
AAAATTAAACTAACTCAACAAGAACTATTTTTCCATCTAATTGGTGGTATGTGACCCCTAGCCCCTAGGGGTG
TAAATTGGGTGGGTTCAATCTAATCCTAGACCAACCCAACTTGCAATTTTAGATGAATTGGCTTGGATTTTTG
CTTTTCGGGATGGGCTTAGGCCTAAGCTCAAAACTTTGGGTTTTTCTATTTTTAGAGTTGTGCCCAAGCCTA
GCTTTAGGTAGTTCAAGCCTAGCCACATATATACATACATACATAGGAATGGCAATGAGGTAGGGCAAGGCT
GAGTTTTGTATGCTCCAGCCTTGACCCCAAATTTAAATACCCTGTGCCTCGTACTCCAACGTGACTAAAAATT
CCAACATCATCCCCATCCCTATAAGGAACGGGGATCCTCAAATTTTCCCTACCCCACCTTGGTCATTCCTATTT
GCAAATCTTTTTTCTTTTATTTTTAAAACCGACCAATCCAAACGCAAGCATAACCTGAGTGCATCAAATTTTAG
TGCGGGCTCGACCCTAATTGCATAGATCAAGATTGACCTTAGGCTCAAGCTTTAGGTCCAATGCATAGGGTT
GGACCTAAATTAAGCACAAACTTGGCCCATCCTGCCATGACTTGCACCCTAATGGTCCAATCAGTTAAGTAAA
ATGGGCCAATTAATGATGTCATAATTAAATAAAAAAAATAGATACAATTACGAAAAACAAAATATAGAAAATTTA
CAAAAAAAACTTAACATATCTCAGATAACATAAATTTGTTATACTAAACAAAGATAAATGTGATAAACAAGAAT
TGTTTTTACAATTAATTATGGGATGAATAAAAATTTTAATAATTCTCGAAATAGATAGAAATAAACAATTATAGA
AAATACATAATTTTTGTCAAAAAAATTTGTGCACATCTCTAATATTATATATTTATCATATTAAACAAAAACAAAT
ATGATAAATAAGAAGTATTTTTATTAACAATAGAAATAAAATACAAAATTTAAATCCACCAACACACCATACTTT
AATTAGGGTCTGTTTGGTTTGCGAGAATAGGGGTTTGGAATTGAAATCAGATTGGCCCATTCTGATTCCAAT
TGTTGCTTGGGTGGAATTGGAATCCCATTTCCATTCCTGATGGAATGATATTCCCCCATTTGAGGGAATATCC
GTTCCATCCCTTCCCTTGGGAATGAAATCCCATTCCAACTAATTTCAATTCACATACTGATTCTTGTCCAAACTA
AACAAACTATGAAAATAGATCATTCCAATTATGATTTTGAGGGTTTTTTATTCCATTCTCACTGTTAGGGGTGT
CAACGGTATTCGAAAATCTGATTTCGAATTCGAGATTCGACATAATCGAATCGAATTCGGATAATTCGAATAG
TTAATCAGTCGAATTTGGATATCCTATTCATACCGAATAGCTAATCGGGTTCGAATTCGGATATGACCTGATTC
GATTCGATAAATATCCAAATTCGATCGATTATTTAATTTTTATATATAATATATAAAATTTTTAAGTGTTTTATATAA
ATTTATATACTTTATATATATAATATTTATTTTATATATTTATTAATTATTTAAAATAATTAATTAGTTGAATTTGAATC
TAATCGAATATTCGAATATTTAGTCGAATTCGAATTCGAATATTAATTTGATATCGAATCGAATTCGAATATCAA
ATTTTTTAATCGGATTCGAATTCGAATATTGTCTGATCCAATTCGAATTTGATCCGTTTACATCCCTACTCACTAT
GAACCAAATGGATTCTTAATTTTGTATGGATTGTGATAAAGATATTGAAATTATCACGTGCATTGGACGTGCAT
GTGTACTAGTCCAAATAAATTTTCTCTTTCCCCTTGCCCTTTCTCATTCCATCCTTTTTCCAGCCGAATTTTCTC
CGGAACCAAACGGACTACTACTGAAAAAAAAAAATATGTTCTTCCTTGATCAGTTAATCGCCTATGATCTTCCT

TCTTCCCTCCCCACCTCCTCTCTTTCATCACGTGACTGGATCTGCGTCTATCGGATCGGTTCCATCCATCTCTTC
ACCCTTTTCCTGCCCTATCAGCCTCGTCCCCTCAAGTTGCATCTGTCTGTAATCGTTTCGGGATCAAAATCGG
GGAATCCAGTGGGCTTGTCGCCGGGACCCGCCGCCATGGAAGTGGCAGCACGAGATTCGACCCAGCCATC
GGCCACTGCAGAGGAAGAGGCCCTCAAGAAGAACACGGATTGTGTTTATTTCCTTGCATCTCCTTTGACCT
GTAAAAAGGTACAATTTTGTTTTCAACGCTGCTTTGCTTCTATTTTTGGGGTTTCTGTGGTTGTTTTGATTGA
ATATTAGGGAGAGTTTAAAATTAGCATGATGGACTTGTTCTTTTTTAATCTTGATCGACAAAAAAAGGTAGTA
TTTTCTTTGAATTTGTTTTTTCTCATCGAATTCGAAGAATAACGTTTTCAGTTGCGTCTTTCTCACAGTAAAGT
TTGGATGGGGTGTTTAAAGATGTTGTTTTGATGGTTAATCTAATAGGAAGAGCATACTAAGAATTGAAGGAA
TTCGGTTTGGAGCTTCGTCTTGGAAGCTTGGAAGTTTCAGAGAGTGTGCTGGTCTACGGTTTTCTTTTTAAT
TTTGACATACTATGGTTTCTCTGTTTGTCTGAGTGAGGAAGAACATAGCGTATGATGAAGAAATAACCGGAA
GGCTTCTGCCTTTTATGTTCTTTGGTCACGAGAGCATACTTACTAGTTTTCCAAGATGATTGTTGGACGGAAT
TGAAATGTCATCCATATGACCGTCCTAACAGCTTAGCCAACTAACTTCTGAAGTAATATATGTTGGTTTCTTGC
TTAATGTGTTCAGACTGAAGGAGGCACCTTTGTGTTCTTTAAAATGGAGTGGGGAATTTGTCCTTCCTGACT
CCTGAGTTATATTTGATATACTGATGTTCCTTGCTCATTCCTTCTAAAGGGTATCTTCTCAGAGACATGATTGGA
ATGAAATAATAAAATAATTGGTGTGATTTCCTGGTTCAGCTTCCAATTTATAGTTAGAATTGCATTTATTACTTA
CTAGTGGGTTCTGACATTACAAGGTTGGTTAGGGGTATTAGTTCCCATAGGTAAACTCCACAGAAGTACCTC
CCAGGACATAATTTTACATTTTCTTTCCAGAAATACCTGGTTAGAAGCCAATGAAAGGGGGGGGGGGGTAA
AAAGAATGGCCTTTTTCTGACCAAAACCCTTTAAAACTCATCCTTTATGAGGATGGGCTGGGTTGGAGTGAA
TAAGAAGAATGGATTATGGCCCAGCCAGAATTATTCCATTATTGGTGTGGAGTGAGAGGGCATCATCTCCCT
TTTTTCTCCACCCTCTTCCCTGCCCACCCCCACCCCCAAAAGAAGAAAAGGAAAAAATGGTAGCTGTTTTTC
TTAATCAATTTTATGTGCCATTTTAGTCTTTGTTAGTTTGATATGATATTGTTAATTTCTGTGGAATGAGATCCA
AGGGGCTAAATATCATCATCATCATCATCTAGCCATATCCCAACTGTTGCATTGAATATATTAGCAGCATAAATC
TGATGTCTAATGTAGTGTAGGTCAGTTGTGTTTTTCATTTCCTTTGCCTTTCTCTTTCAGTGTATCAGACCTTTT
TGGCCCTATACTGATCCAATAAATTCCTCTGTATGACTGTATCCTATAAGAAAAGTGGGGCAGAGCGTAGAAG
CTTACTTCTGCTATTATATTTAGTATTAGAAGTATGATAAAAAGTAAACAAAGATGTCTTATTTTAAACTTACTA
GTAGTGCCCTGTGCAATGCATGGGAAAGCCAAGTTGAACCATAATGAAAGTGGCCAGTTTAACATAATATAT
TCTAACTTCGTAAAAAATTAACTAAGGTGGCATTTGGCAAGTCTTTTGGAGCGGTCTTCTGGGCTTCGACAA
ACTCAGAAGACCAAAAACTGGTCTTTTTATGTTTGGTAACCAAAAGGTGGTAAGACTGGAAGACGCAAGC
GCGGAAAGACCCGAAAAGACTGAATACCCTGAATTTTAGGAAGTTTCAAATGAAGCTTTCTGGTCTTTCTGT
GCGATAAATAGCCTCTTAACGTGTTTTTAAGAGCCCGGTAGACCAAGCCACTTGCCAAACGATTTTTTTTCTC
TTTAAAGACAAGACCCCAAAAGCTCAATACGTCTTTTGGCCATCTGGGCTGCTGGAAGATCTTTCTAAAAAG
ATTTGTCAAACAACACCTAAGTAATCTTAGTAAGCTTCTCATTCTAAGGATAAGAATCATGATTATGTCTTATAT
TGCTCTTTTTATTGTCTATTTTGTATCTTAGCAACTAACAAAACAATTTCTCGTGTATATCTGTGGTATCTTGAG
CAAGTAATGTGCCTTTGTTGACGCTTTAGCATTTTATAACATTTTGTAAATTGCCACCTTTTTTCATGGCATCTC
AAACACTTCTACCATAAACATATTCATTATTTCTTGGCTAAATTCCTGCAATATTAATGTTTTTATTTAAAAGAA
AAGTTTTTGCATACTTGTCTTTTCCTCAGAATTGCAAGTACAAACCCATCCTTAGAGAAGGAACAAGTCCTTC
AACACTGCATCCTCGGTGCTTGAAACCTCCTTCAATCTATACACATCTTTTATTAAGCTCTATTGGTAGTTGGA
ATGTATGAAACTTCTAGCTTAGTATATGTTTGGTTCCGTGGGTGTTTCCTGAGGGGCGTTTTCTCAGATAAAT
GTTTACCAAAAAATGCTGCTTTAAACCCTTTTTTTTTTGCCATTTCTATTTGGTTGGGCATTTCAAGTAACACC
CCATAAAATTGCTTTCTACTTCAGCTTAAAGTGCACCATATCAGCATTTCATTTCAAGACCCAGGCATTGGGG
GAAACGAGAATTTGATGTTTCCCCAAATGCCCAATGCACCTAAGCCAAAGCGTTTCAGATTTTGTGTTTTTTT
TTGATAAATGGTGAAACCAAACAGTGGTTTCATATGCAATAAACACCAACGCCTATGTGGCACTGATGTGGC
AGCATTTTTTTAATAAACACCCTCAGAACCAAACATAGCCTTAGTGTTGATCTATATCCTGACTTTCCAGAGTA
TTAAGACAATCCTTTTTCACATTAATCAGATGTGGGATATCTCTGACCAACTTTTGAAGAGATTGGAACTGGA

AAAAGATGGAGAAATTGTCTTTGTACTATATGTACTTAAATATTATGCAGTTGTACCTTTAGCCAGTGATTATAT
CAGGAGACCTGGCTATTATTTATTTATTTACTTCTGTAGGATTAATGTTTCTTCTTCATTTCTATCACTCATTATA
CTATCAATACTGCTTGTTTATTATCCTTCTCATTAGTTGTATCCCTAAGAATCTTGTTCTTATGTTTTTTTCTCCAT
CAAACAGGGAAGCGAATGTGAATATCGCCATAGTGAACCTGCTAGGATTAACCCCAGAGATTGCTGGTTCTG
GATGAATGGCAATTGCCTAAATCCAAAATGTTCATTCCGACATCCGGTGAGTCTTTTTTGTTTACTTTTCATGA
TGGATGTTGTAATGATTGTTCTTGTCTTTCCATTAGCTTTTTTATTTTGTTTTGAACTGACAACATGGATGGGT
CCTTTTTATTTCATAAATCACCACAATCTTGTAATGTGAATGATTTTCAATGCCTAATTTATGTCTATTATTTCTTT
TCCAGCCTTTGGATGGCTTGTTAGGAACCTCAGGGGCATCTTATGTGGGATCATATATGCCTGCACATACTGG
GGCATCAACAAAAATTCTTCCATTGCATGCTCCCGCTTATAGTTCAGCTAAACAATCTATCCCCTGTATTTTCTT
CCAAAAGGGACTTTGCTTAAAAGGTGACAGATGCCCCTTCACACATGGACCACAACCAACTAGTAATCTGCC
CTCACAGCCACCATCACCACCACCAAAAGTTGTTCTTACTACTGAGCCTCAGGATTCAAATAAGGCTTCTTTT
TGGGGGCTTGAGAAATTCACCAAACAACAGCAGATCCCAAGACAAAATTTTGTAAGCCAAGTTGAAGTACC
TTCTTCAGCAAAATCAGTTACAAAGCCTGAGATTGCATTGTCGAAAAATGGAGTGCCAGTTAAGAAGAGTG
TACCACCAATAACTGTATCAGATGATGAGTGTCTTAGATATAAACCAGCAAATGGTTCTGGTATCAGTGGAAG
TTCCATAAGTAGGCCATCAAATGGCCCTGGCATCATTGGAAATTCTTTAAGTAGGTCTCACCGTAGTCGTCAA
GCTCAGGTGGATGAGCAGAGTCTACAGAATGGCAAAGAGGGTGATGATTACTTGGGTGAATCCTCACCTGG
CTTTGATGTACTCGTAGATAATGAGCTCAGTGATTATGATTATTACCAAAAGGAAGATGATGTTGGAAGGACA
ACAAGTCATAATGGAAGGCATTTGACCTCTGTGAATGAATTTGATTACGACAACTGCTCTGTTAATCACAGTT
CAATGAATAAATTTGACAGGGAGCCATACAATAATGCACGTGAGTATGACCCATATGGACGGTCTCACGATCA
TTATGCATGGCAACAGAGTAGAGCTTCATCTGAGAGGGTTACAGAGAGGCTATCTCTACCAGAACAAAGGG
GGTTCACAAGAAGTGAGAACCCTGATCAGATTAGTGAGTCAGATTTGCGCCACCAGCTATCGAAGCAAAGA
AGGGTTGGTGGATCAAGATCTGCCATTAGTTCAGATCGTCATGGTGATCACCATTGGAGGGTTGATCATGAC
CACCACATTGAGGATCAAAGAAACCTGAGTCAATCTTGGAGAGAGTCACGTCATTTGCCTCAAGCGGGTTC
CATAAGCAATCGGCTGCGAGGTAGAGTTACGTTTCCTGGTAGATCTTCACCAGACAATAACAATACCAATGG
CATGCATCCAGAGCGGGAAATGGACAGGGGAAGAAAAAGGGGCAGGTTGTCACCAGGCAGAGCACCAGT
ATTGTCCTACCAGGGAAGGCACCAAAGCAGATTAAAACGCAAAAGTGAGGACTTAAACATGGAGGAGAGA
AATTGCAGGGCTCCTCTTGTCAAAAGAGATGAGGTAGGGAACAGCTTGGATTTTGCTGGTCCAAAAAGCCT
TGCAGAACTGAAAGGTGCTAAGGTTACAGAGAGCAAAGAAGACCACCAGCAAGGCAAAGATTGTCAAGTA
TTTTCTGTTGGAGAGCAGAGAAACTCTAAGATTGGAAAATGTGTTGCTCAAGAATACGAGGACTCACTTTC
CTTTGAGAGTCCCAAGCCGCTAAGCATGATTTTGAAGAGGAAAAGAGTAGTAGAAACCGCAGCTTCTGGTA
ATAATGAGGTTTCTGGAATGGGAGATAGTGACATTCAGAGGGAAGGGAGTGAAAACCATGTGGGCAACTC
CAGCATCACAACAGTTGCAGAGATACAGACTGTTCTTTCTTCTGATCTCAAAAAAGAAGCCAATGAAAGCAC
AGTCAGCAATCAGGAAAATCCTATAAATTCAGCTGCCCTAATTGACGATGAAGAAGGTGAGATCTTTCCAGA
GGATGAAGAAGTGGCTCATGAAGGGGAGTCATATGCACAAGATGGGTCTGAGGTCGAGATTGATGATACTA
TGGAAGATCAAGAACTTGAAACCTTTGATAAGAGAGATGGTGAATCTGATTATGAGCAAGCTGATGATGAC
CAAGATTATAGGACAGAGGAAGTTGAAAATATGGACCCTGAAGAGGAATACTTGGACGATGAAGATGGGG
ATGACTTTGCAAAGAGGATTAGCATCATGTTCTCTGGAGAGAGAGAGAGATGAAACTGATTGCTCATTCCCA
ATGGTAAAAGACCATGTTAGCATTATTTTTTTATTTAAATATAGAATGGCTCTTTTTAAATGAGCTAATGTAGCT
CATTGTTTTCATTTAAATATAAAATGGCTCGTACAACTGAGCTGATAAAGCTTGCATTCTGGGTTATGCTCAAA
AGTGGGAACCTTGTTATTGTATTAATGGAGTCTTGTTTCAATGTATTTAAGTTGATCTAATGGAAATTTCTTCG
TTCTCGTTTTCACTTTGCAAACTGTGTCCAGTAAGCTTTTAAACTTCAAAACTTCTAAATTCCTCCAAGGCAAT
TCTTCTCAACGAAATCCTATTAGTCTCTAACTTCATGGCCTCGGTGAGGAAAGGCGCCACTGTGAGAAGTTA
AATGGTTGTGGGTTCATGTATTTTATTACGGGCGGTGGAACATTATCATTGATTCTACAGCCCACTGAACAGC
TAGCTATTGCACCCAGCTAATCCTCCTCCCCATTGAGAACTGGTGTCATAATGGGGAACACATGCCAACCCAA

GTGGGGTTCTAACTAAGGACCTGGGCTCCACATGTTAGCATTTGCAATTTTGTTCTGCTTTATAAAGACTAAA
AATGCGTAGAGCTTTTATCTATCCAAATTTAGTAATCTCTTTTTTCTCGACCCTTTCAGATACCAACAAATGGA
AGAAAGATTCAATTGTGGGAACACTCTTT
