# Supplementary material for: Molecular evolution and functional characterisation of an ancient phenylalanine ammonia-lyase gene (NnPAL1) from Nelumbo nucifera: novel insight into the evolution of the PAL family in angiosperms
Source: BMC Evol Biol. 2014 May 9;14:100. doi: 10.1186/1471-2148-14-100 (PMC4102242; doi:10.1186/1471-2148-14-100)
Supplement: Additional file 7 — Homology search for the upstream sequence (31,942 bps) and downstream sequence (26,288 bps) against the nucleotide collection database in NCBI. [file 1471-2148-14-100-S7.pdf]

Additional file 7. Homology search for upstream sequence (31,942 bps) (a) and downstream sequence (26,288 bps) (b) to nucleotide collection database in NCBI, respectively.

(a) upstream sequence of *NnPAL1* **(b) downstream sequence of *NnPAL1***

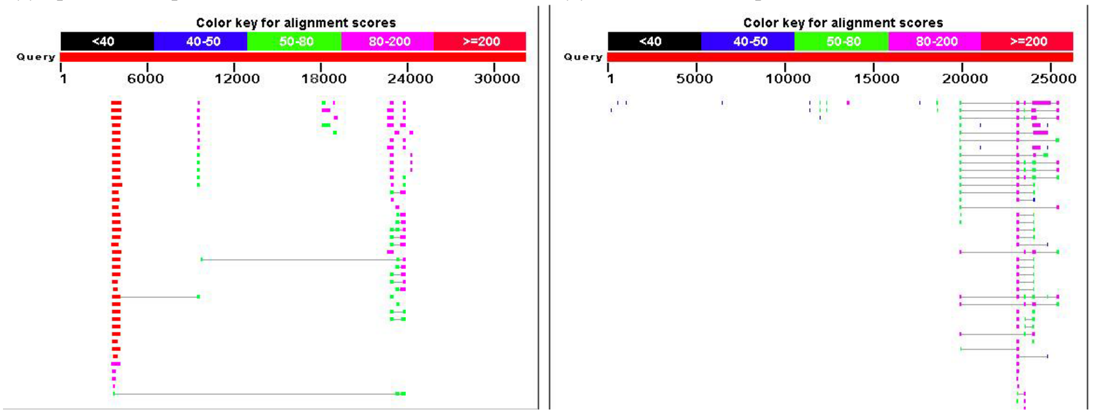

Alignment I. Homology search for upstream sequence of *NnPAL1*.

Homologous regions of dicots with the first three highest scores upstream 1-5000 bps are *Vitis vinifera* [uncharacterized LOC100852583 (LOC100852583), mRNA], *Populus trichocarpa* [tetratricopeptide repeat-containing family protein (POPTR_0010s10880g) mRNA, complete coding sequence] and *Ricinus communis* conserved hypothetical protein, mRNA (XM_002518755.1|), respectively.

(1) PREDICTED: *Vitis vinifera* uncharacterized LOC100852583 (LOC100852583), mRNA. Sequence ID: ref|XM_003631297.1| (e-value, 9e-105)

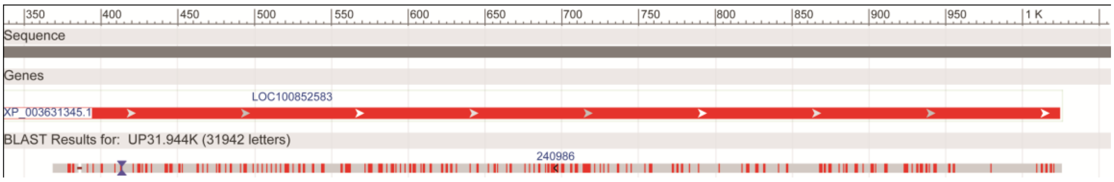


```
Query  3602  TCAATTATCTGAGTCTGTCCCAAACAGCTTCATCCTCGACAATGGAGTCTGCAAGAACCC  3661
             |||| | || || || ||||||||||||||||||||||||||||| ||||||||||||||
Sbjct  1024  TCAACTCTCCGAATCAGTCCCAAACAGCTTCATCCTCGACAATGGGGTCTGCAAGAACCC  965

Query  3662  CTCCACCTCGAATTTCTTGGGACAAAGTTCGTGGTATTTCTTAAACTGCTCTCTCGCCTC  3721
             ||||||||| || ||||||||  || | ||  | || ||   |||||||||||  |||||
Sbjct  964   CTCCACCTCAAACTTCTTGGGCGAACGCTCCCGATACTTAGCAAACTGCTCTCGAGCCTC  905

Query  3722  CTTGTTCCGATCAAGCAAGCTATAAATCACCCCTTGGCAAAAGTAAGGCCTGAAATCCTT  3781
              |  |||| |||  ||||| | || ||||  || |  ||||||||||||||||||||  |
Sbjct  904   TTCATTCCTATCCTGCAAGGTGTATATCATTCCCTTACAAAAGTAAGGCCTGAAATCACT  845

Query  3782  GGGGTCCTCCTTGGCCAATTCCTGATAACTCCTCAATGCCTCATCCACATTCTTCTGAAG  3841
             ||| ||||||||  |||  ||||  || |||||||||||||| |||||||||||| ||||
```

```
Sbjct  844   GGGATCCTCCTTCTCCAGCTCCTCGTAGCTCCTCAATGCCTCGTCCACATTCTTCCGAAG  785

Query  3842  ATACTGAATCTGTGCCATTATCAACCTTACGTCGCGAGCTTCTTTCACCTTGTTTTCCTC  3901
             | |||| || || |||||||||||||  || |||||||| || ||||  ||||| || | 
Sbjct  784   AAACTGCATTTGGGCCATTATCAACCGCACATCGCGAGCCTCCTTCAGTTTGTTCTCATT  725

Query  3902  CTCTGCAAGTTTCAGGGCTTCTTGAAGCCGGTTAAGCACTGCTTCACCTTCTCCACAGCG  3961
             ||| ||     |||  || ||||  || |   | | ||| || || || ||||||||  |
Sbjct  724   CTCAGCCGTCCTCAAAGCGTCTTCCAGTCTCCTGATCACCGCGTCGCCCTCTCCACATTG  665

Query  3962  GTCCATAAGCAAGGCATTCTCGAATAGAGCTTCGAAAGAGAGAGGATTCACAGCCAAAAT  4021
              ||||| | ||| |||||| |||| |||||||| || || || ||||||  ||| | |||
Sbjct  664   ATCCATTAACAATGCATTCCCGAACAGAGCTTCAAAGGACAGCGGATTCGAAGCAAGAAT  605

Query  4022  AGCTTCAAAAACCTGCCGAGCTTCATCAACTTTCCCCATTTCATTCAATAATCTCGCCAC  4081
               | || || || |  || ||   ||||   | |||||||||    |  |||||||||| 
Sbjct  604   TTCCTCGAATACGTCGCGTGCCCTATCAGTCTCCCCCATTTCCCCTAGCAATCTCGCCAT  545

Query  4082  GAGAAATTTCCATTCAACCTCCGATGGTTGAGCAGAAACCAAGCGTTCCAATATCTTCAA  4141
              ||||| ||||| || ||| ||   || || || || || || || ||||  | ||||| 
Sbjct  544   CAGAAACTTCCACTCCACCACCTCCGGCTGCGCCGACACTAACCGCTCCAGGACCTTCAG  485

Query  4142  AGCTTCCTCATCCTCTCCATCTTCCAGCTTCCGTTGCAAGAGAGATTTCAAGGCGTCAAT  4201
              || ||| | ||||| || || ||||||||| | ||||  |  |||||||| ||  | | 
Sbjct  484   TGCCTCCCCGTCCTCGCCGTCCTCCAGCTTCTGCTGCAGCACCGATTTCAACGCCCCCAA  425

Query  4202  GGCGTCAGAATGCGATTCTAGAAACTGAGACAGCGGAGAT---TCCTGGGATTTCTCCT  4257
              || |||||||    ||| |||||||  |||| ||| |||   || |  ||||||||||
Sbjct  424   AGCCTCAGAAT---TTTCAAGAAACTCGGACAACGGCGATGACTCTTCAGATTTCTCCT  369
```

(2) *Populus trichocarpa* tetratricopeptide repeat-containing family protein (POPTR_0010s10880g) mRNA, complete coding sequence. Sequence ID: ref|XM_002314704.2| (e-value, 7e-94)

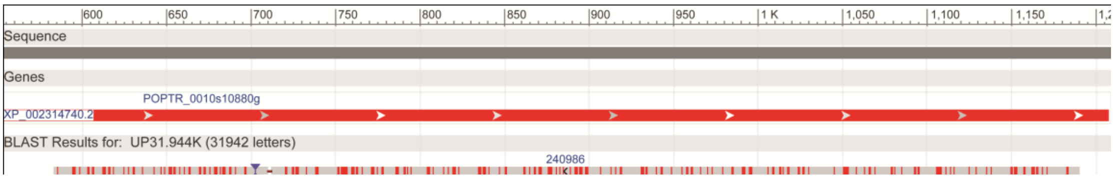


```
Query  3620  CCCAAACAGCTTCATCCTCGACAATGGAGTCTGCAAGAACCCCTCCACCTCGAATTTCTT  3679
             |||||| ||||||||||| || || |  |||  |||  | ||||||||||| || |||||
```

```
Sbjct  1189  CCCAAAAAGCTTCATCCTTGATAACGAGGTCCTCAAATATCCCTCCACCTCAAACTTCTT  1130

Query  3680  GGGACAAAGTTCGTGGTATTTCTTAAACTGCTCTCTCGCCTCCTTGTTCCGATCAAGCAA  3739
              ||| |||||||| | || ||   |||||||||| | ||||||| ||| | |||||||||
Sbjct  1129  TGGAGAAAGTTCGCGATACTTGGCAAACTGCTCTTTTGCCTCCTCGTTTCTATCAAGCAA  1070

Query  3740  GCTATAAATCACCCCTTGGCAAAAGTAAGGCCTGAAATCCTTGGGGTCCTCCTTGGCCAA  3799
              || || |||| ||||   ||||| |||||||||||||| || || ||||| || | |||
Sbjct  1069  ACTGTATATCATCCCTCTACAAAAATAAGGCCTGAAATCTTTAGGATCCTCATTCGACAA  1010

Query  3800  TTCCTGATAACTCCTCAATGCCTCATCCACATTCTTCTGAAGATACTGAATCTGTGCCAT  3859
              || |||||||||  ||  ||||| ||||| ||||| || ||| |||| || || |||||
Sbjct  1009  CTCTTGATAACTCTCCAGCGCCTCCTCCACGTTCTTTTGCAGAAACTGTATTTGAGCCAT  950

Query  3860  TATCAACCTTACGTCGCGAGCTTCTTTCACCTTGTTTTCCTCCTCTGCAAGTTTCAGGGC  3919
              ||||| || ||||| |  ||||||||||| || || ||||| |||||||  |  |  ||
Sbjct  949   AATCAATCTCACGTCCCTCGCTTCTTTCACTTTATTCTCCTCTTCTGCAATATCTAAAGC  890

Query  3920  TTCTTGAAGCCGGTTAAGCACTGCTTCACCTTCTCCACAGCGGTCCATAAGCAAGGCATT  3979
              ||| | | ||   |||  || || || |||||||||| ||| ||||| |  |  |||||
Sbjct  889   CTCTCGTAACCTCCTAATAACCGCGTCTCCTTCTCCACTGCGCTCCATTAATAGTGCATT  830

Query  3980  CTCGAATAGAGCTTCGAAAGAGAGAGGATTCACAGCCAAAATAGCTTCAAAAACCTGCCG  4039
             |||||| |  || || ||||| |  ||||||||| | | |||  ||||||| || |  ||
Sbjct  829   CTCGAACAATGCCTCAAAAGACAAGGGATTCACAACGAGAATTTCTTCAAACACATTGCG  770

Query  4040  AGCTTCATCAACTTTCCCCATTTCATTCAATAATCTCGCCACGAGAAATTTCCATTC---  4096
             ||| || |  ||    | |||||||||||  ||||| ||||  | ||| ||||||||
Sbjct  769   AGCGTCTTGGACCCGGCGCATTTCATTCAGCAATCTTGCCATCAAAAACTTCCATTCAGT  710

Query  4097  AACCTCCGATGGTTGAGCAGAAACCAAGCGTTCCAATATCTTCAAAGCTTCCTCATCCTC  4156
             |||||||   ||||| ||||| || ||  | |  || || || ||||| || || || |
Sbjct  709   AACCTCC---GGTTGCGCAGATACTAACTGCTTTAAAATATTTAAAGCCTCGTCGTCTTG  653

Query  4157  TCCATCTTCCAGCTTCCGTTGCAAGAGAGATTTCAAGGCGTCAATGGCGTCAGAATGCGA  4216
              || | ||| |||||| |||| || || ||||| |  |  ||||| || |||| ||  ||
Sbjct  652   GCCCTTTTCAAGCTTCTGTTGTAAAAGTGATTTTAGAGACTCAATTGCTTCAGCATTAGA  593

Query  4217  TTCTAGAAA  4225
             |||||| ||
Sbjct  592   TTCTAGGAA  584
```

(3) *Ricinus communis* conserved hypothetical protein, mRNA. Sequence ID: ref|XM_002518755.1|, (1e-90)

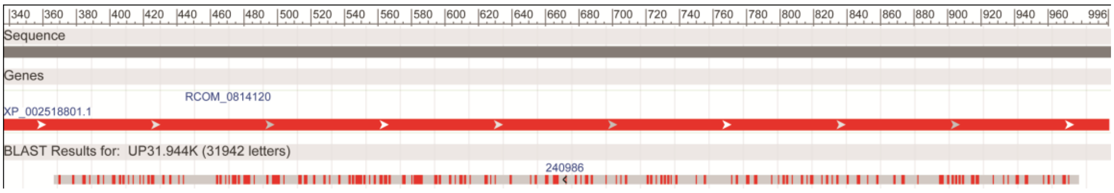


```
Query  3621  CCAAACAGCTTCATCCTCGACAATGGAGTCTGCAAGAACCCCTCCACCTCGAATTTCTTG  3680
             ||||| |  ||||| || || |||||||||  |||  |||| || ||||| |||||||| 
Sbjct  977   CCAAAAACTTTCATTCTTGATAATGGAGTCCTCAAATACCCTTCTACCTCAAATTTCTTT  918

Query  3681  GGACAAAGTTCGTGGTATTTCTTAAACTGCTCTCTCGCCTCCTTGTTCCGATCAAGCAAG  3740
              |  |||| | | | || ||   |||||||||||| |||||||  |||| ||||||||| 
Sbjct  917   CGGGAAAGCTGGCGATACTTAGCAAACTGCTCTCTTGCCTCCTCATTCCTATCAAGCAAA  858

Query  3741  CTATAAATCACCCCTTGGCAAAAGTAAGGCCTGAAATCCTTGGGGTCCTCCTTGGCCAAT  3800
             ||||| ||||  ||||| ||||| || || || |||||  | || ||||| || | || |
Sbjct  857   CTATATATCATTCCTTGACAAAAATATGGTCTAAAATCACTTGGATCCTCTTTTGACAGT  798

Query  3801  TCCTGATAACTCCTCAATGCCTCATCCACATTCTTCTGAAGATACTGAATCTGTGCCATT  3860
             || ||||||||  |||  ||||| || ||||||||||||||| |||| ||||||||||| 
Sbjct  797   TCTTGATAACTTTTCAGGGCCTCCTCTACATTCTTCTGAAGAAACTGTATCTGTGCCATA  738

Query  3861  ATCAACCTTACGTCGCGAGCTTCTTTCACCTTGTTTTCCTCCTCTGCAAGTTTCAGGGCT  3920
             || | | | || || | |||||||||||| || || ||||| |||||||  |  || || 
Sbjct  737   ATAAGCTTCACATCTCTAGCTTCTTTCACTTTATTCTCCTCTTCTGCAATGTCTAGAGCC  678

Query  3921  TCTTGAAGCCGGTTAAGCACTGCTTCACCTTCTCCACAGCGGTCCATAAGCAAGGCATTC  3980
             ||||| ||||   |||  ||||| || ||||||||||| |||||||| || |  ||||||
Sbjct  677   TCTTGTAGCCTCCTAATAACTGCATCCCCTTCTCCACATCGGTCCATTAGAAGTGCATTC  618

Query  3981  TCGAATAGAGCTTCGAAAGAGAGAGGATTCACAGCCAAAATAGCTTCAAAAACCTGCCGA  4040
             || || |  || || ||||| |  |||||||     | |||  ||||||| |  |  || 
Sbjct  617   TCAAACAATGCCTCAAAAGATAACGGATTCATTTGGAGAATTTCTTCAAACAAGTTACGT  558

Query  4041  GCTTCATCAACTTTCCCCATTTCATTCAATAATCTCGCCACGAGAAATTTCCATTCAACC  4100
             || || |    | | ||||| ||||| || ||||| ||||  |  |||||||| ||    
Sbjct  557   GCATCCTGTGTTCTACCCATCTCATTTAACAATCTTGCCATCAAGAATTTCCACTCCGTA  498

Query  4101  TCCGATGGTTGAGCAGAAACCAAGCGTTCCAATATCTTCAAAGCTTCCTCATCCTCTCCA  4160
              || ||||||| ||    || |   | | || | ||||||||||||||||||| || |||
Sbjct  497   ACCAATGGTTGTGCCTCGACAAGATGGTTCAGTGTCTTCAAAGCTTCCTCATCTTCCCCA  438

Query  4161  TCTTCCAGCTTCCGTTGCAAGAGAGATTTCAAGGCGTCAATGGCGTCAGAATGCGATTCT  4220
```

```
             | ||| |||||  | || | ||| || || | ||  ||||| || |||| ||  |||||
Sbjct  437   TTTTCAAGCTTTTGCTGTAGGAGTGACTTGAGGGATTCAATTGCTTCAGCATTGGATTCA  378

Query  4221  AGAAACTGAGA  4231
             ||||||| |||
Sbjct  377   AGAAACTCAGA  367
```

Alignment II. Homology search for downstream sequences of *NnPAL1*.

Homologous regions of dicots with the first three highest scores downstream 20,000-25,000 bps are *Vitis vinifera* (contig VV78X242760.3, whole genome shotgun sequence), *Citrus sinensis* (PREDICTED: zinc finger CCCH domain-containing protein 17-like (LOC102617828), mRNA) and *Populus trichocarpa* [zinc finger family protein (POPTR_0008s14320g) mRNA, complete coding sequence], respectively. Also, aligned regions in the section of 10-15K are homologous to partial coding sequence of *GWD* gene for alpha-glucan water dikinase of *Nelumbo nucifera* itself.

(1) *Vitis vinifera* contig VV78X242760.3, whole genome shotgun sequence, Sequence ID: emb|AM427456.2| (e-value, 3e-34)

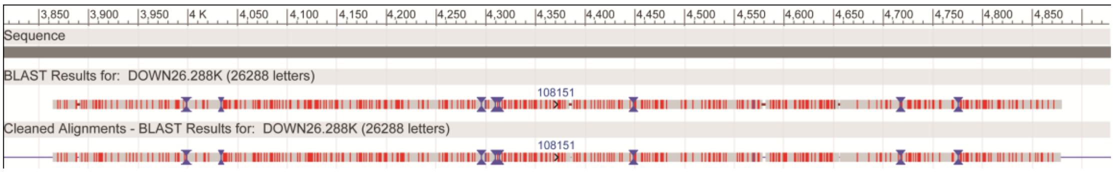


```
Query24040   GATGAGCAGAGTCTACAGAATGGC---AAAGAGGGTGATGATTACTTGGGTGAATCCTCA  24096
               ||||| || ||| | ||||||||    || || | |||||| |  |   | || |||||
Sbjct3865    GATGATCACAGTTTTCAGAATGGTGGTAAGGATGYTGATGAGTTTTATAGAGAGTCCTCT  3924

Query24097   CCTGGCTTTGATGTACTCGTAGATAATGAGCTCAGTGATTATGATTATTACCAAAAGGAA  24156
             ||||| |||||||| || || ||| |||| ||||| |||| |||||| || ||  | |||
Sbjct3925    CCTGGTTTTGATGTCCTTGTTGATGATGATCTCAGAGATTCTGATTACTATCATGATGAA  3984

Query24157   GATGATGTTGGAAGGACAACAAGTCATAATGGAAGGCATTTGACCTCTGTGAATGAATTT  24216
             ||| |  ||||| |            | |||||||| ||||||  ||| |||||||||||
Sbjct3985    GATCAGTTTGGAGG------------TGATGGAAGGAATTTGAATTCTATGAATGAATTT  4032

Query24217   GATTACGACAACTGCTCTGTTAATCACAGTTCAATGAATAAATTTGACAGGGAGCCATAC 24276
             |   ||     | | |||  | || ||| |||||||  | |  | ||| | ||    | |
Sbjct4033    G---ACATTGGCCGATCTTCTGATTACAATTCAATGGCTGATATGGACCGAGAAATGTTC  4089

Query24277   AATAATGCACGTGAGTATGACCCATATGGACGGTCTCACGATCATTATGCATGGCAACAG  24336
             | || ||| ||  |||||| | ||||  |     || |  || ||| | ||| | ||
Sbjct4090    CGTGATCCACATGGCTATGACTCCTATGAGCACATGCATGGGCAGTATCCTTGGGATCAT  4149

Query24337   AGTAGAGCTTCATCTGAGAGGGTTACAGAGAGGCTATCTCTACCAGAACAAAGGGGGTTC  24396
```

```
            | ||  ||||||| |||||| |   || | ||  | |||  |  |||   ||||| |||
Sbjct4150   CGCAGGTCTTCATCAGAGAGGATGCTAGTGGGGGCAGCTCCTCTGGAAAGGAGGGGTTTC  4209

Query24397  ACAAGAAGTGAGAACCCTGATCAGATTAGTGAGTCAGATTTGCGCCACCAGCTATCGAAG  24456
               | |  ||||| ||||||||| || | ||| ||||||||||| || |   |||| |||
Sbjct4210   CGTAAATCTGAGAGCCCTGATCAAATCAATGAATCAGATTTGCGACATCGCTTATCAAAG  4269

Query24457  CAAAGAAGGGTTGGTGGATCAAGATCTGCCATTAGTTCAGATCGTCATGGTGATCACCAT  24516
            || || || |||  ||| | ||||||       ||||  | | ||||||      || |
Sbjct4270   CACAGGAGAGTTAATGGTTTAAGATC-------AGTT--GTTAGTCATG------ACTA-  4313

Query24517  TGGAGGGTTGATCATGACCACCACATTGAGGATCAAAGAAACCTGAGTCAATCTTGGAGA  24576
                    ||| |  ||| | ||   ||| ||    ||  | |   |||  ||| | ||
Sbjct4314   --------TGACCTAGACAATCATGCTGAAGAGAGGAGTTATCGTGGTCCGTCTCGAAGG  4365

Query24577  GAGTCACGTCATTTGCCT---CAAGCGGGTTCCATAAGCAATCGGCTGCGAGGTAGAGTT  24633
            || ||||  | | | |||   || | | | ||| | |||| ||| || || || ||| |
Sbjct4366   GATTCACACCCTGTACCTACACATGAGAGCTCCCTTAGCAGTCGCCTTCGGGGCAGAATA  4425

Query24634  ACGTTTCCTGGTAGATCTTCACCAGACAATAACAATACCAATGGCATGCATCCAGAGCGG  24693
            | | ||||  | |||||||| ||      |  ||||   | || | ||| |   ||   |
Sbjct4426   AAGCTTCCCAGGAGATCTTCTCC------TGTCAATGGTAGTGACTTGCGTGTTGAAAAG  4479

Query24694  GAAATGGACAGGGGAAGAAAAAGGGGCAGGTTGTCACCAGGCAGAGCACCAGTATTGTCC  24753
            || |||||||||||||| ||  ||||||| |||||||| || ||   || | | |  |||
Sbjct4480   GATATGGACAGGGGAAGGAACTGGGGCAGATTGTCACCTGGAAGGCAACAAATTTCCTCC  4539

Query24754  TACCAGGGAAGGCACCAAAGCAGATTAAAACGCAAAAGT----GAGGACTTAAACATGGA  24809
             | ||||||||||  | |  |||| ||| | | ||  ||    ||||| |  |  |  ||
Sbjct4540   CAGCAGGGAAGGCTTCGAGACAGAATAAGA-GGAAGGGTTCAAGAGGATTCCATTACTGA  4598

Query24810  GGAGAGAAATTGCAGGGCTCCTCTTGTCAAAAGAGATGAGGTAGGGAACAGC-TTG--GA  24866
             |  |||||||   || | |  |   ||| ||| |||   || |   |  || |||  ||
Sbjct4599   TGCAAGAAATTCTTGGACCCAGCGCATCAGAAGGGATATAGTGGATGATGGCGTTGCTGA  4658

Query24867  TTTTGCTGGTCCAAAAAGCCTTGCAGAACTGAAAGGTGCTAAGGTTACAGAGAGCAAAGA  24926
            ||||||||||||||| ||||||||||| ||||||| ||  |||  ||| |||||||| |
Sbjct4659   TTTTGCTGGTCCAAAGAGCCTTGCAGAGCTGAAAGTTGTGAAGAATACGGAGAGCAAGG-  4717

Query24927  AGACCACCAGCAAGGCAAAGATTGTCAAGTATTTTCTGTTGGAGAGCAGAGAAACTCTAA  24986
                 | |||| |   || | |   |||  ||| ||  |||||  |||||||||||  ||
Sbjct4718   -----AGCAGCGAATGAAGGTTCAACAATCATTGTCCCTTGGAAGGCAGAGAAACTTAAA  4772

Query24987  GATTGGAAAATGTGTTGCTCAAGAATACGAGGACTCACTTTCCTTTGAGAGTCCCAAGCC  25046
```

```
              |||      |   | |   ||| |||  || ||    || || |||||  | || |||||
Sbjct4773     GAT------AGAGGGTCAGCAACAATCTGAAGATGACCTATCGTTTGAAGGGCCGAAGCC   4826

Query25047    GCTAAGCATGATTTTGAAGAGGAAAAGAGTAGTAGAAACCGCAGCTTCTGG   25097
               || ||  ||||| ||||||| ||||| | ||  ||||| |||| ||||||
Sbjct4827     TCTTAGTGTGATTCTGAAGAGAAAAAGGGGAGCTGAAACAGCAGTTTCTGG   4877
```

(2) PREDICTED: *Citrus sinensis* zinc finger CCCH domain-containing protein 17-like (LOC102617828), mRNA. Sequence ID: ref|XM_006470838.1| (1e-31)

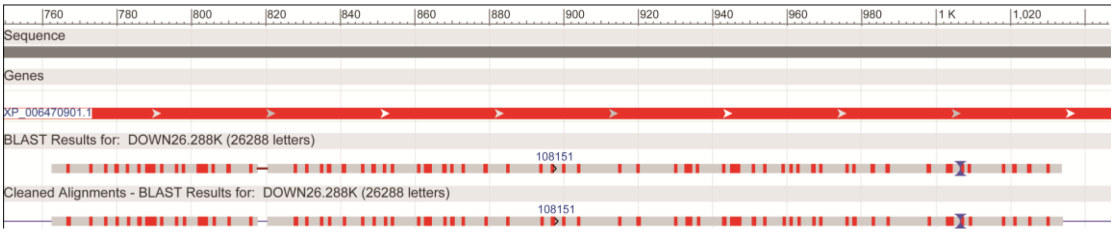


```
Query23983    CCTGGCATCATTGGAAATTCTTTAAGTAGGTCTCACCGTAGTCGTCAAGCTCAGG---TG   24039
              |||| ||||| ||| || || || |   | ||| | |||   | ||| ||||| |   ||
Sbjct763      CCTGTCATCAATGGTAACTCCTTGATCCGCTCTAATCGTTTGCATCAGGCTCATGCACTG   822

Query24040    GATGAGCAGAGTCTACAGAATGGCAAAGAGGGTGATGATTACTTGGGTGAATCCTCACCT   24099
              ||||| || ||| | ||| |||| || || | |||||| |  ||| | || ||||| |||
Sbjct823      GATGATCAAAGTTTCCAGCATGGTAAGGATGCTGATGAGTTTTTGAGGGAGTCCTCTCCT   882

Query24100    GGCTTTGATGTACTCGTAGATAATGAGCTCAGTGATTATGATTATTACCAAAAGGAAGAT   24159
              || ||||||||| || ||| |||||||||| |||| |||||||||| ||  | ||||||
Sbjct883      GGATTTGATGTTCTTGTTGATGATGAGCTCAGAGATTCTGATTATTATCATGATGAAGAT   942

Query24160    GATGTTGGAAGGACAACAAGTCATAATGGAAGGCATTTGACCTCTGTGAATGAATTTGAT   24219
               |   ||| || |||| | | ||| | |||||| | |||| ||| ||||||||| |||||
Sbjct943      CAATATGGCAGAACAAGAGGGCATGAAGGAAGGGACTTGAACTCAGTGAATGAATATGAT   1002

Query24220    TACGACAACTGCTCTGTTAATCACAGTTCAATGA   24253
                ||    | |||||||| || ||| |||| |||
Sbjct1003     ATCG---GCCGCTCTGTTGATTACACTTCATTGA   1033
```

(3) *Populus trichocarpa* zinc finger family protein (POPTR_0008s14320g) mRNA, complete cds.Sequence ID: ref|XM_002312469.2|

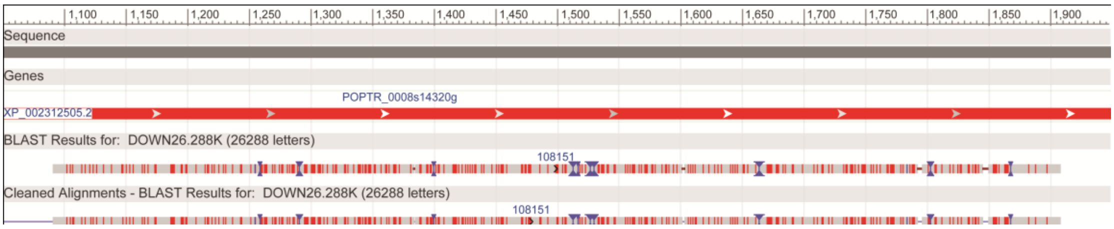


```
Query24055    CAGAATGGCAAAGAGGGTGATGATTACTTGGGTGAATCCTCACCTGGCTTTGATGTACTC  24114
              ||||||||||| || | |||||| || ||| | || ||||| ||||| |||||||| ||
Sbjct1091     CAGAATGGCAAGGATGCTGATGAGTATTTGAGGGAGTCCTCTCCTGGTTTTGATGTTCTT  1150

Query24115    GTAGATAATGAGCTCAGTGATTATGATTATTACCAAAAGGAAGATGATGTTGGAAGGACA  24174
              || || |||||| | || ||||  |||||||||||    |||||  |  ||||||| |||
Sbjct1151     GTGGACAATGAGTTGAGGGATTCAGATTATTACCACGGTGAAGAGCAGCTTGGAAGAACA  1210

Query24175    ACAAGTCATAATGGAAGGCATTTGACCTCTGTGAATGAATTTGATTACGACAACTGCTCT  24234
              ||| |  || | || ||| | |||| ||||||  |||||| |||  |||    |   |||
Sbjct1211     ACAGGAAATGAAGGGAGGAACTTGAACTCTGTTGATGAATATGA-CACG--GGCCATTCT  1267

Query24235    GTTAATCACAGTTCAATGAATAAATTTGACAGGGAGCCATACAATAATGCACGTGAGTAT  24294
              | | || |  ||| | |  || |   ||||   ||    ||   | || |||| |  |||
Sbjct1268     GCTGATTATGGTTTATTACATGA---TGACCAAGAAATGTATCGTGATCCACGGGGCTAT  1324

Query24295    GACCCATATGGACGGTCTCACGATCATTATGCATGGCAACAGAGTAGAGCTTCATCTG--  24352
              ||  | |||| ||   | |  |  ||  |||| ||| | |||   || |||||||| |
Sbjct1325     GATTCCTATGAACTCACGCCAGGGCAGCATGCTTGGGAGCAGCACAGGGCTTCATCAGAA  1384

Query24353    AGAGGGTTACAGAGAGGCTATCTCTACCAGAACAAAGGGGGTTCACAAGAAGTGAGAACC  24412
              |||  ||||  |  |  | |  ||| ||||||    | || | | | | |  ||| | ||
Sbjct1385     AGAATGTTAATGGCA--CCAGTTCTTCCAGAAAGGCGAGGTTACGCGAAAGCTGATAGCC  1442

Query24413    CTGATCAGATTAGTGAGTCAGATTTGCGCCACCAGCTATCGAAGCAAAGAAGGGTTGGTG  24472
              |||| || | |   || |||||||||||  | |   ||||||| ||||| ||||||  ||
Sbjct1443     CTGAGCACACTGAGGAATCAGATTTGCGTTATCGTTTATCGAAACAAAGGAGGGTTAATG  1502

Query24473    GATCAAGATCTGCCATTAGTTCAGATCGTCATGGTGATCACCATTGGAGGGTTGATCATG  24532
              | |  |||||       ||||  | | || |||         |||      |||  |  |
Sbjct1503     GCTTGAGATC-------AGTT--GTTAGTAATG---------ATT------TTGTCCCAG  1538

Query24533    ACCACCACATTGAGGATCAAAGAAACCTGAGTCAATCTTGGAGAGAGTCACGTCATTTGC  24592
              |  ||||  ||||||| | | |  ||| | ||   ||  | ||||| || | ||| || |
Sbjct1539     AGAACCATGTTGAGGAGCGAGGTTACCGGGGTTCTTCACGAAGAGACTCTCATCACTTAC  1598

Query24593    CT---CAAGCGGGTTCCATAAGCAATCGGCTGCGAGGTAGAGTTACGTTTCCTGGTAGAT  24649
              ||   || | | | ||||| |||| ||| || || |||||| | | | |||| || ||||
```

```
Sbjct1659   CTCCA---------AACAATGGTGGTGATGTGCGTGCAGAAAGGGAAATTGACAGGGGCA   1709
```

```
Sbjct1599   CTTCACATGAGAGCTCCATTAGCAGTCGTCTTCGTGGTAGAATCAAGCTTCCAGGAAGAT   1658

Query24650  CTTCACCAGACAATAACAATACCAATGGCATGCATCCAGAGCGGGAAATGGACAGGGGAA   24709
            || ||         ||||||     ||   ||| | ||||  ||||||| |||||||| |
Sbjct1659   CTCCA---------AACAATGGTGGTGATGTGCGTGCAGAAAGGGAAATTGACAGGGGCA   1709

Query24710  GAAAAAGGGGCAGGTTGTCACCAGGCAGAGCACCAGTATTGTCCTACCAGGGAAGGCACC   24769
            | ||  |||| || |||||||| |  ||  | || ||    ||| | || || ||||  |
Sbjct1710   GGAATCGGGGAAGATTGTCACCGGTGAGGTCCCCGGTCACCTCCCAACAAGGGAGGCTAC   1769

Query24770  AAAGCAGATTAAAACGCAAAAGT----GAGGACTTAAACATGGAGGAGAGAAATTGCAGG   24825
                |||| | ||| || |  ||    ||||| |   |||  |||| ||||| || |||
Sbjct1770   GGGACAGAATGAAA-GCTAGGGTGGAAGAGGATT---ACAATGAGGGGAGAAGTTTCAGA   1825

Query24826  GCTCCTCTTGTCAAAAGAG-----ATGAGGTAGGGAACAGCTTGGATTTTGCTGGTCCAA   24880
            | |||||  || | | |||     |||    | ||||   ||  |||||||||||||| |
Sbjct1826   GGTCCTCAAGTGAGAGGAGAATTAATGGATGATGGAAGTACT--GATTTTGCTGGTCCTA   1883

Query24881  AAAGCCTTGCAGAACTGAAAGGTG   24904
            |||| |||||||| ||||||||||
Sbjct1884   AAAGTCTTGCAGAGCTGAAAGGTG   1907
```

(4) *Nelumbo nucifera* itself *GWD* gene for alpha-glucan water dikinase, partial cds. Sequence ID: dbj|AB548406.1| (e-value,5e-18)

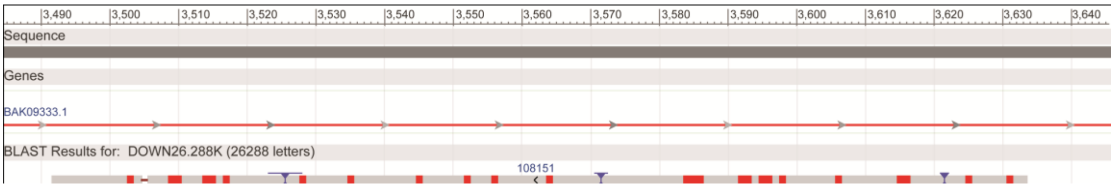


```
Query13585  TTTGGGTTGGACTCCGTCCAAATCAAAATCAATTTGGACAGACTGGATAGACCAATAAGA   13644
            || ||||| ||| |||||  |||||||| ||||||| |  |  |||||   |||||||||
Sbjct3633   TTAGGGTTTGAC-CCGTCTGAATCAAAACCAATTTGAATGGGTTGGATGATCCAATAAGA   3575

Query13645  CATATTGAATTGGACCAGTCTAGACTTAATGATCTAAAGTATTTATTGGATCGGACTGGA   13704
            |||  ||||||| ||||||| ||| |||||| ||||||||| |||||| ||     ||||
Sbjct3574   CAT--TGAATTGAACCAGTCCAGATTTAATGGTCTAAAGTACTTATTGTAT-----TGGA   3522

Query13705  CCTGATTCATCCATAA-GAATTTATATTGG   13733
            |||| |  |||  ||| | |||||||||||
Sbjct3521   CCTGGTCTATCTTTAACGGATTTATATTGG   3492
```
